# Supplementary material for: The rising burden of female cancer in Ethiopia (2000–2021) and projections to 2040: Insights from the global burden of disease study
Source: PLoS One. 2025 Oct 7;20(10):e0333787. doi: 10.1371/journal.pone.0333787 (PMC12503343; doi:10.1371/journal.pone.0333787)
Supplement: S1 File — Supplemental fig 1. Sub-national prevalence, incidence and mortality rate of breast, cervical, ovarian and uterine cancer in Ethiopia (2000–2021). Supplemental fig 2. the Sub-national DALYs, YLD, and YLLs trend of Female specific cancer in Ethiopia (2000–2021). (DOCX) [file pone.0333787.s001.docx]

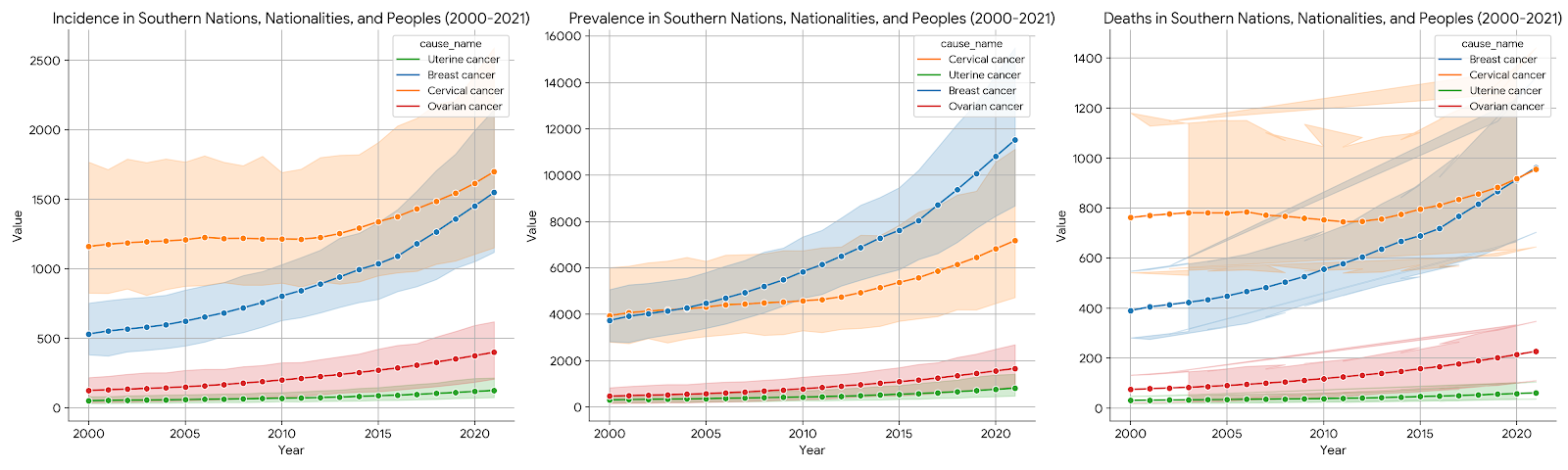


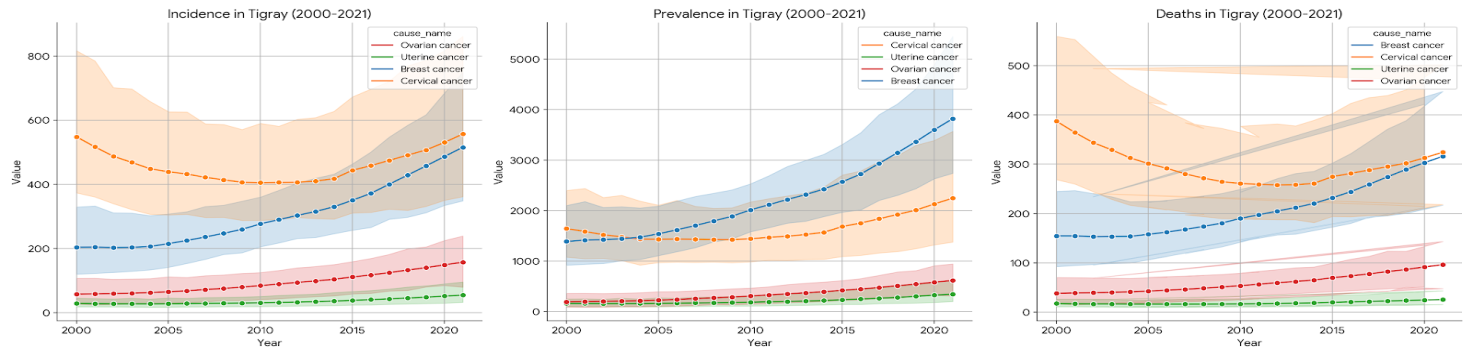


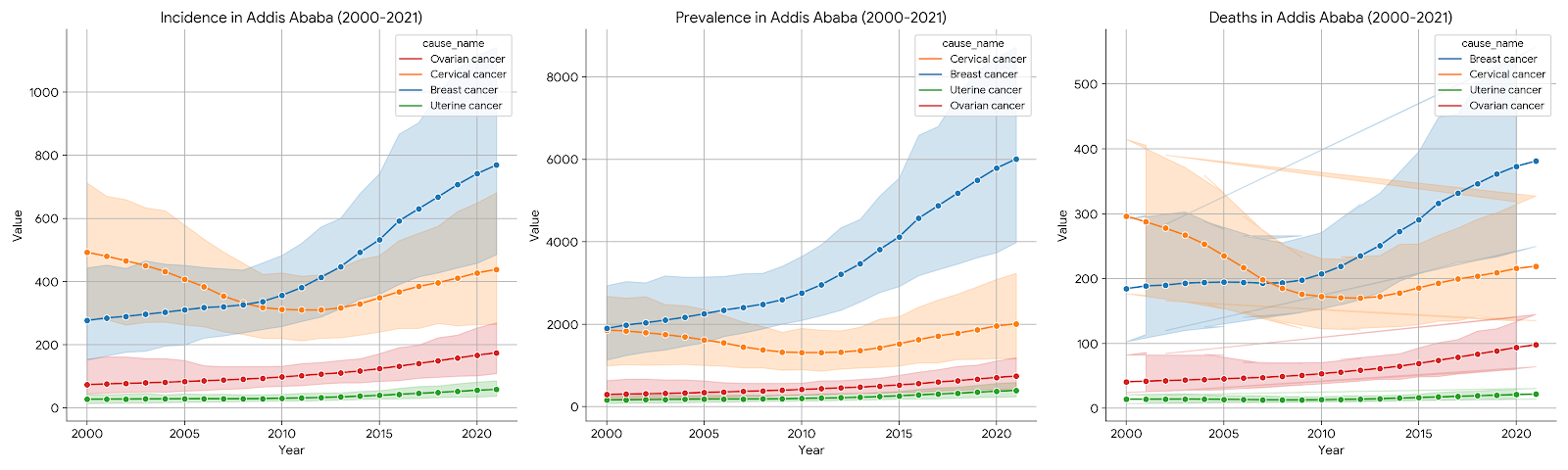


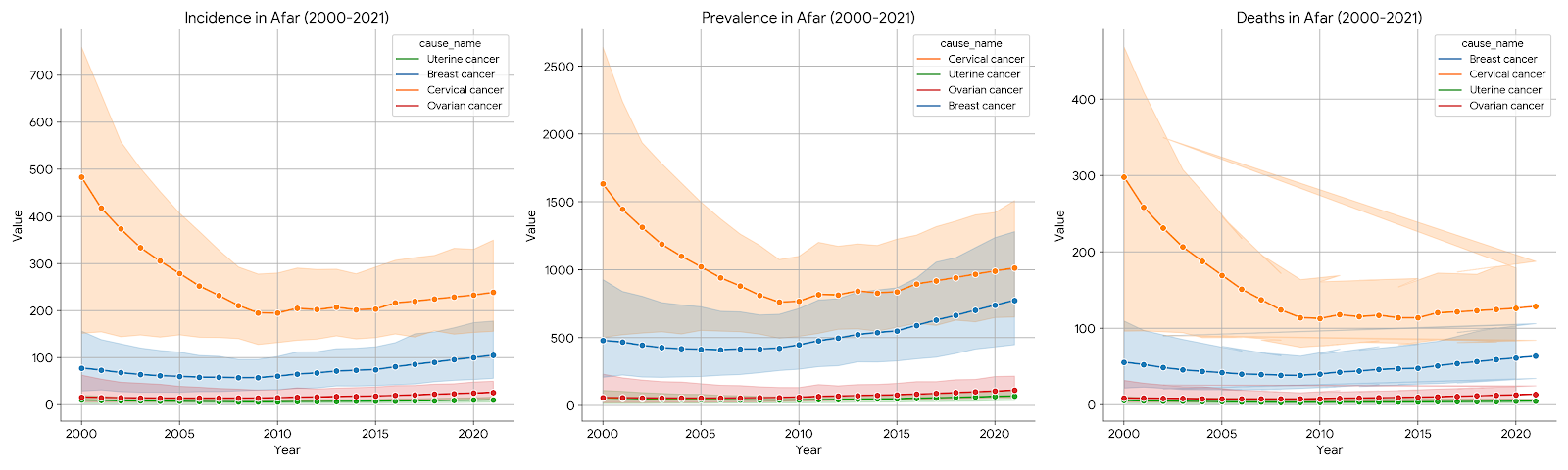


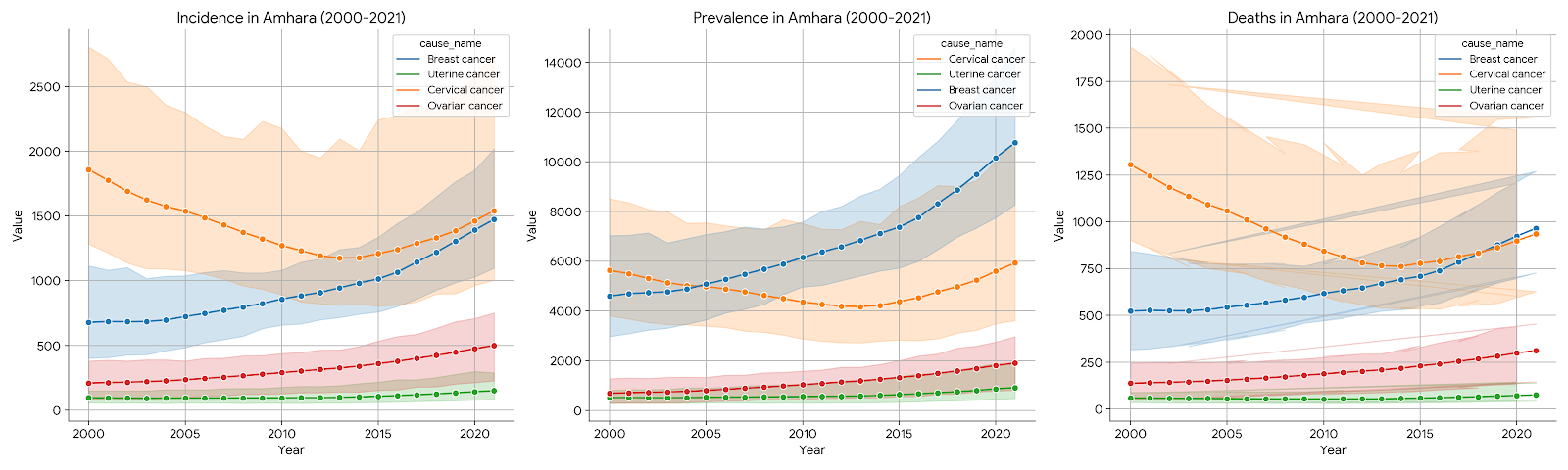


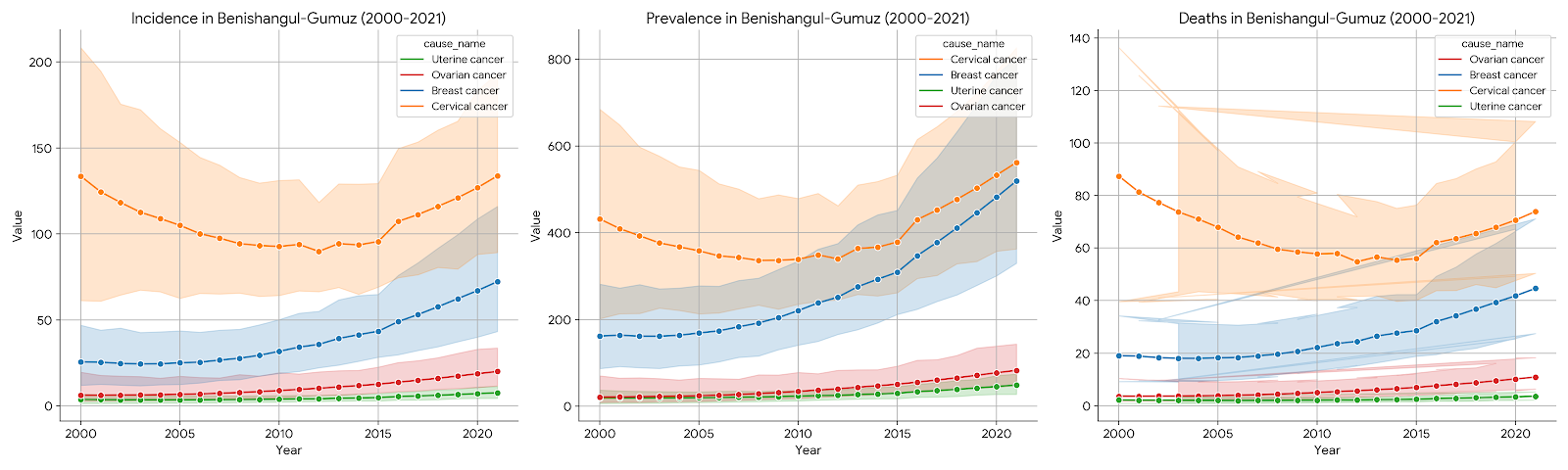


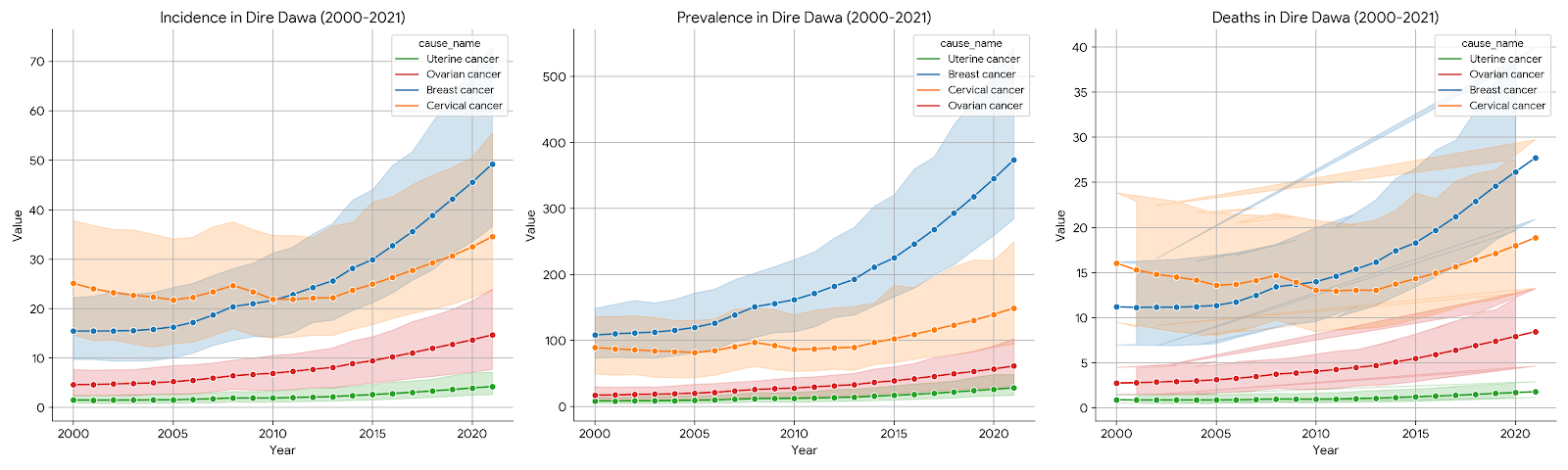


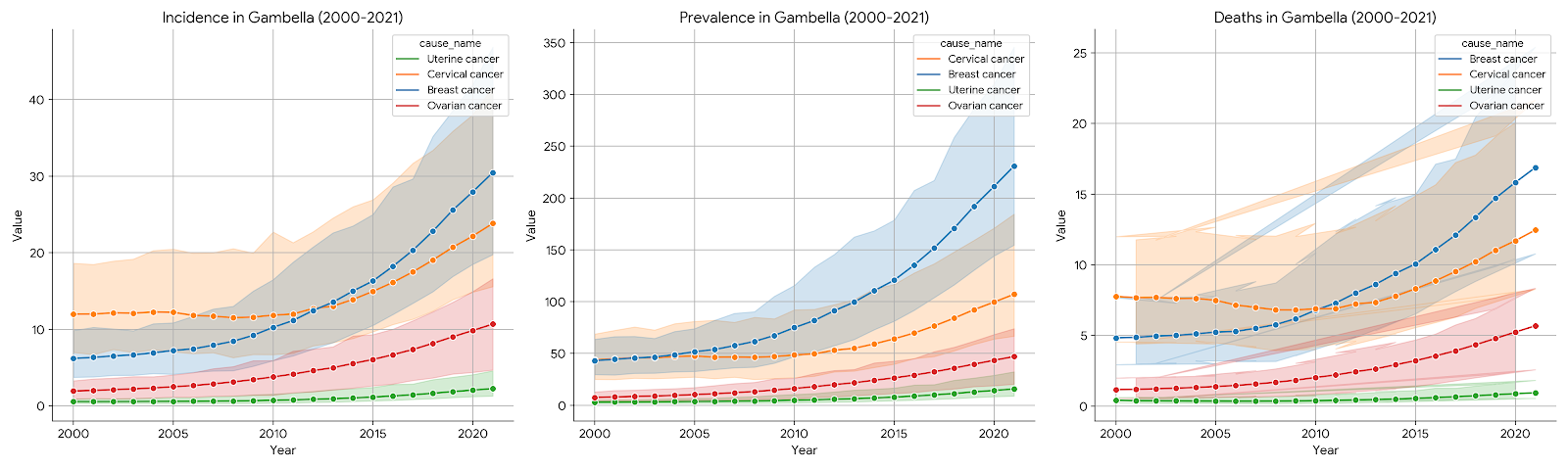


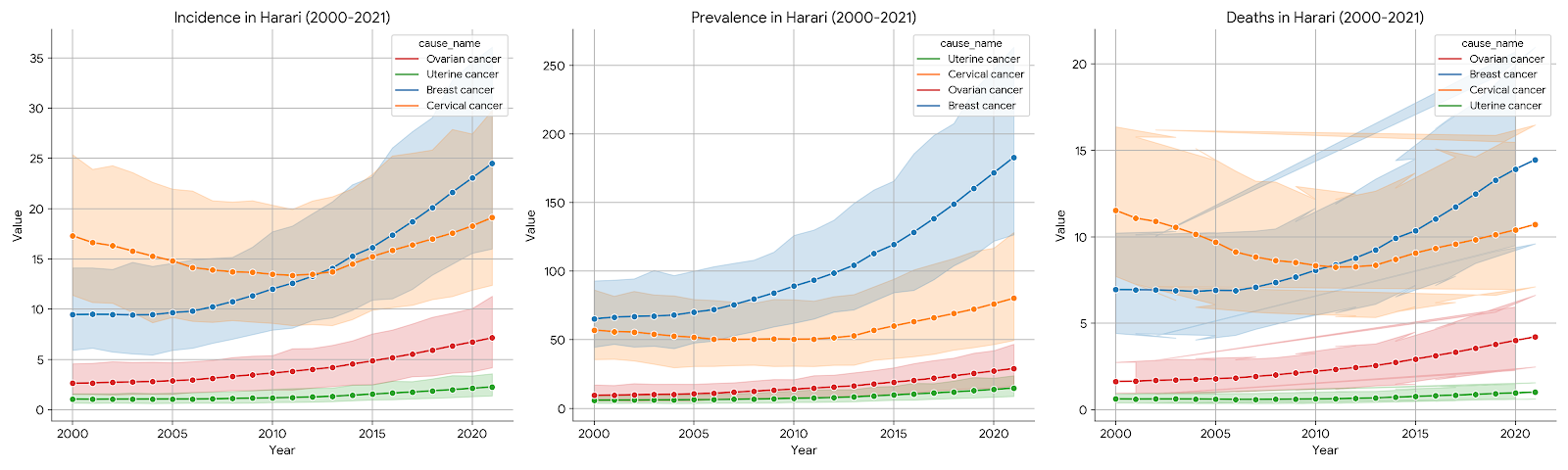


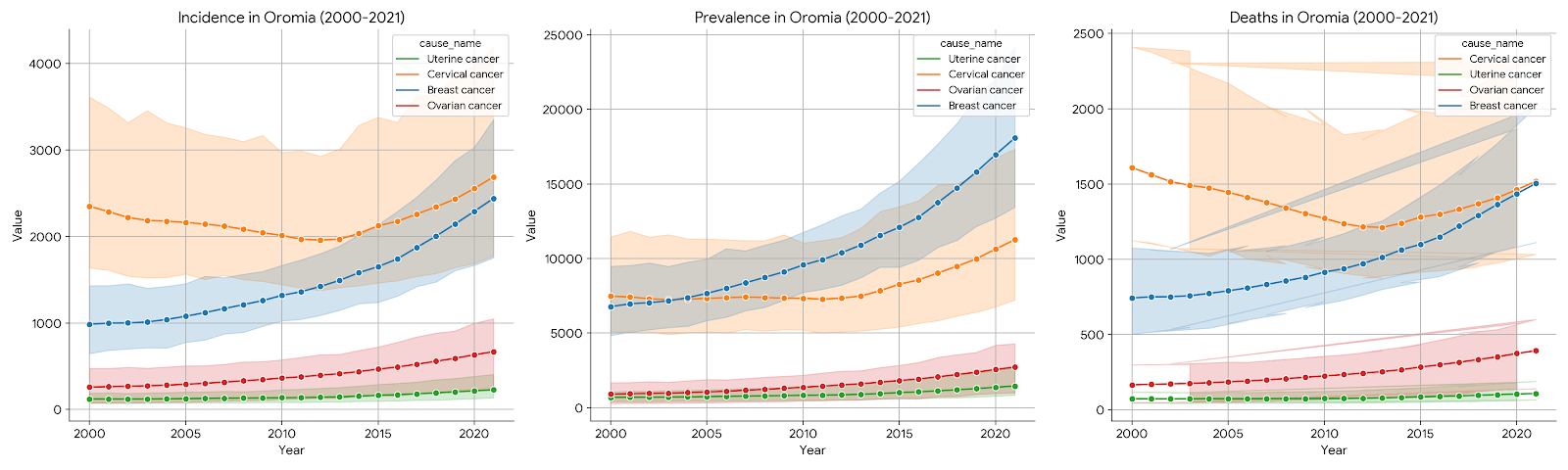


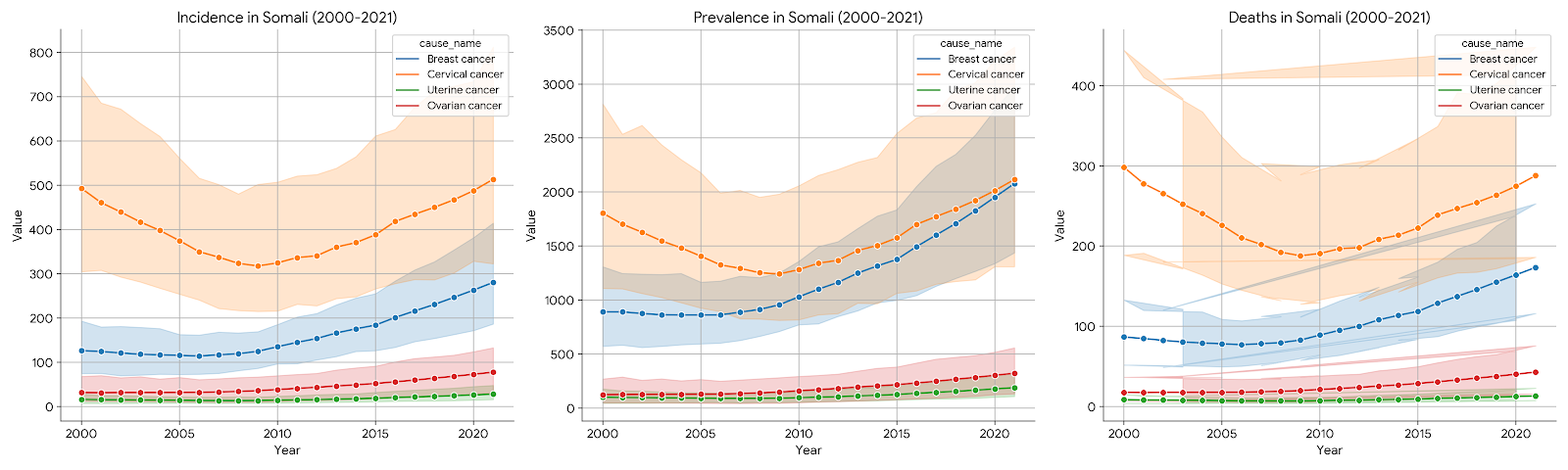


Supplemental fig 1: Sub-national prevalence, incidence and mortality rate of breast, cervical, ovarian and uterine cancer in Ethiopia (2000-2021)


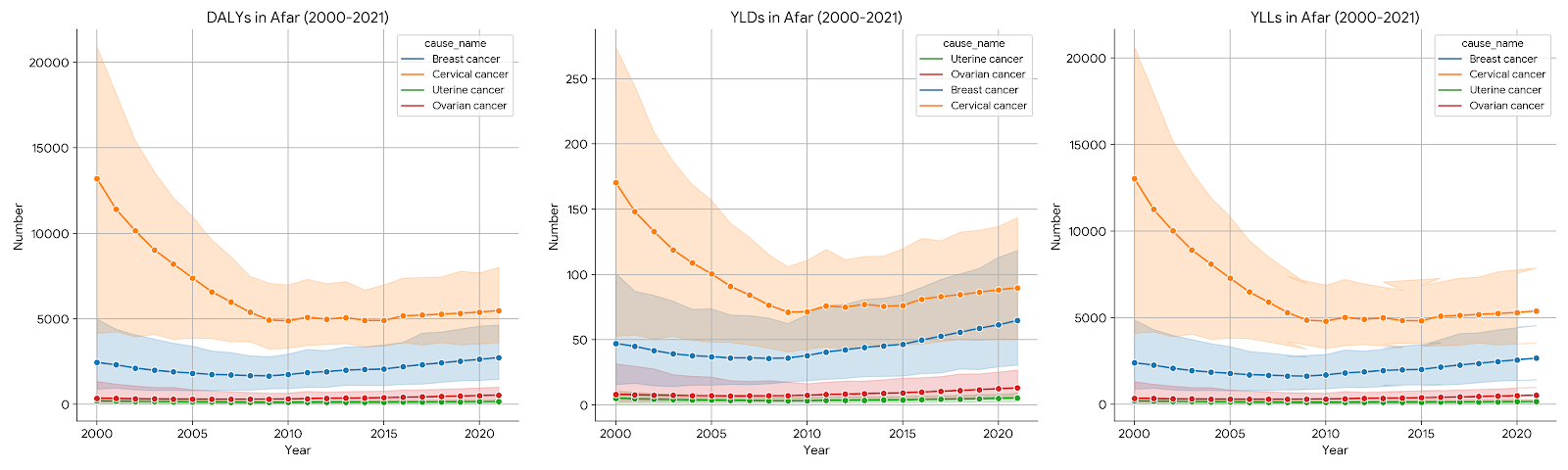


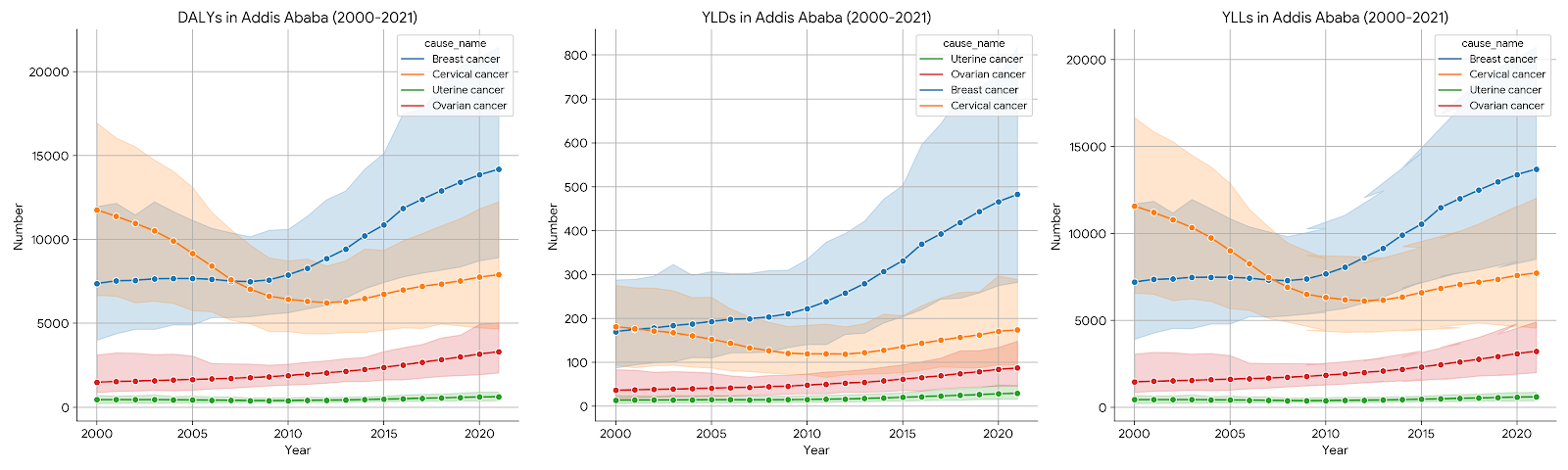


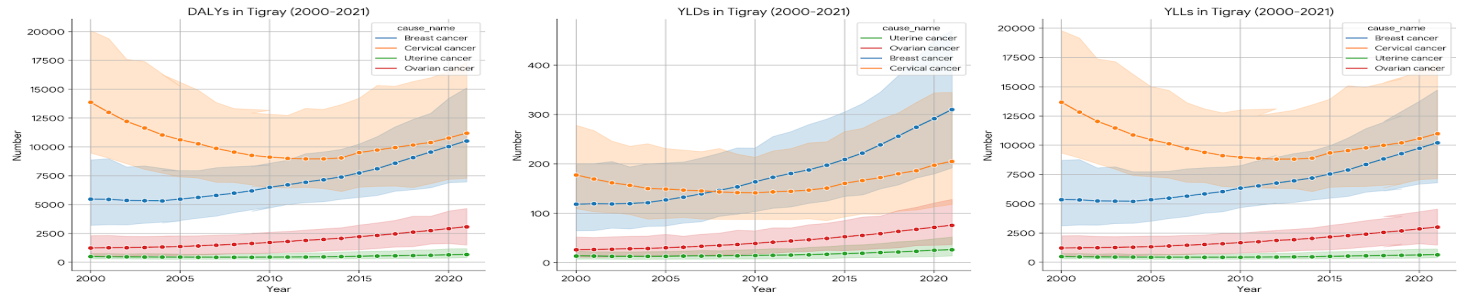


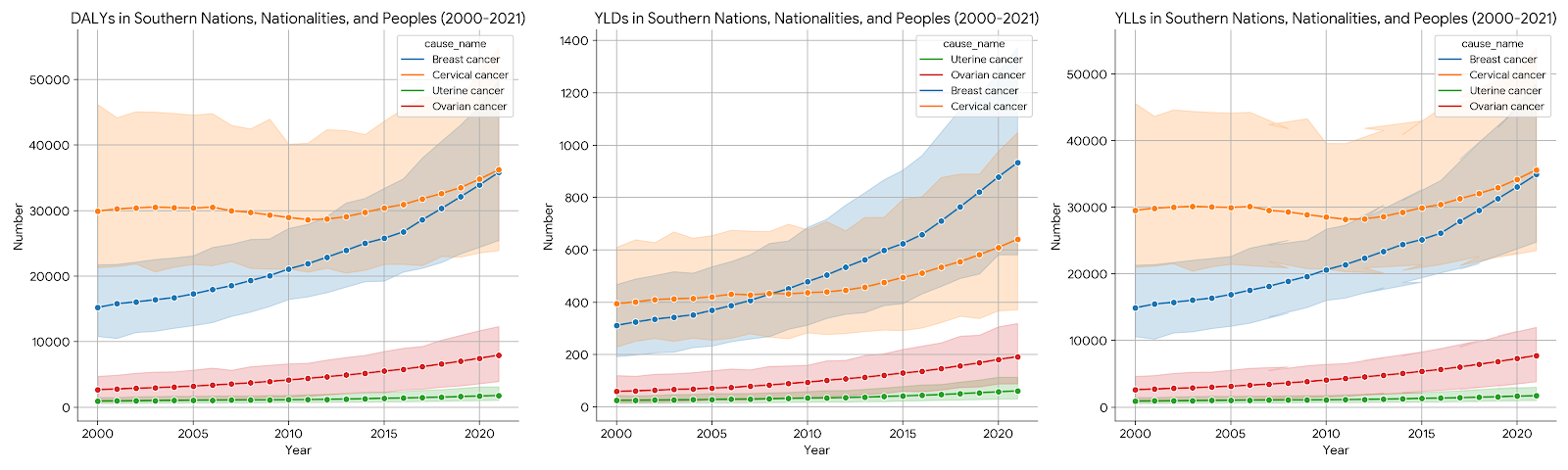


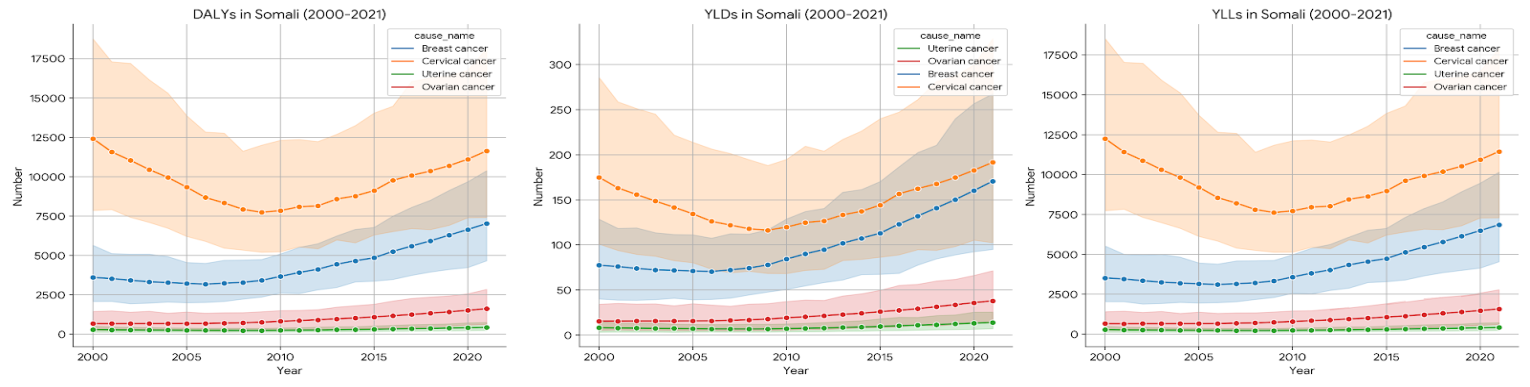


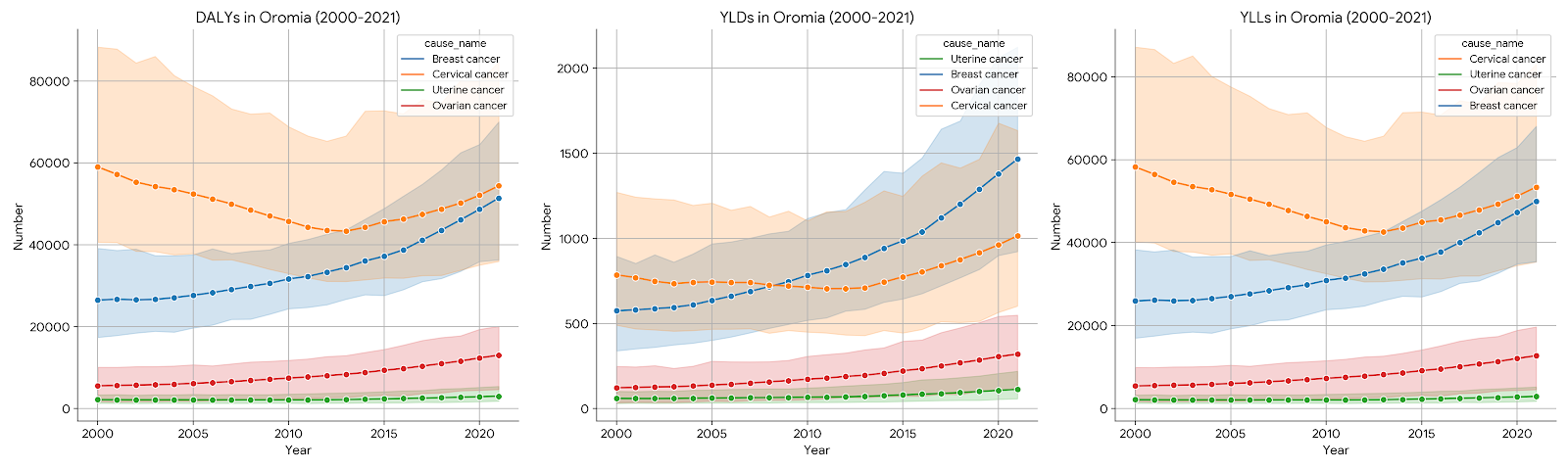


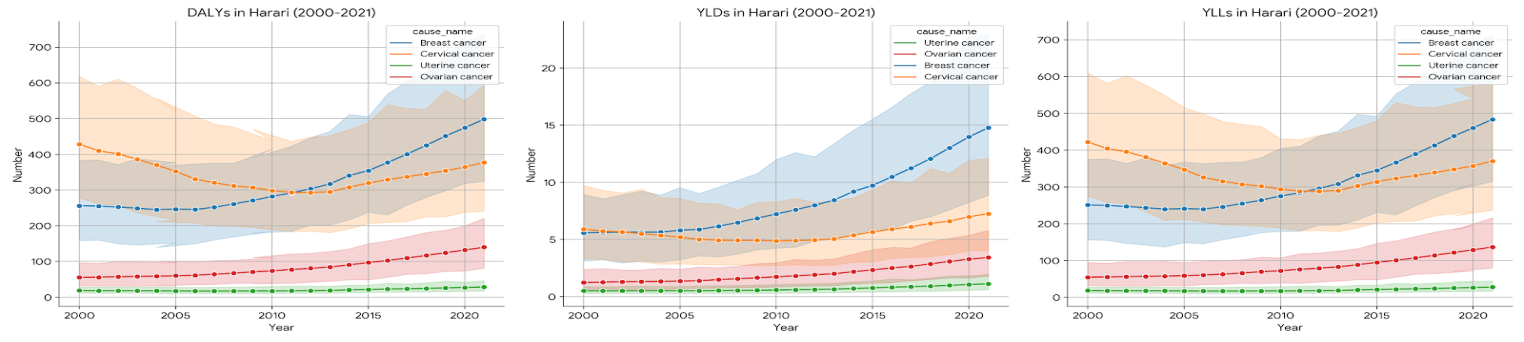


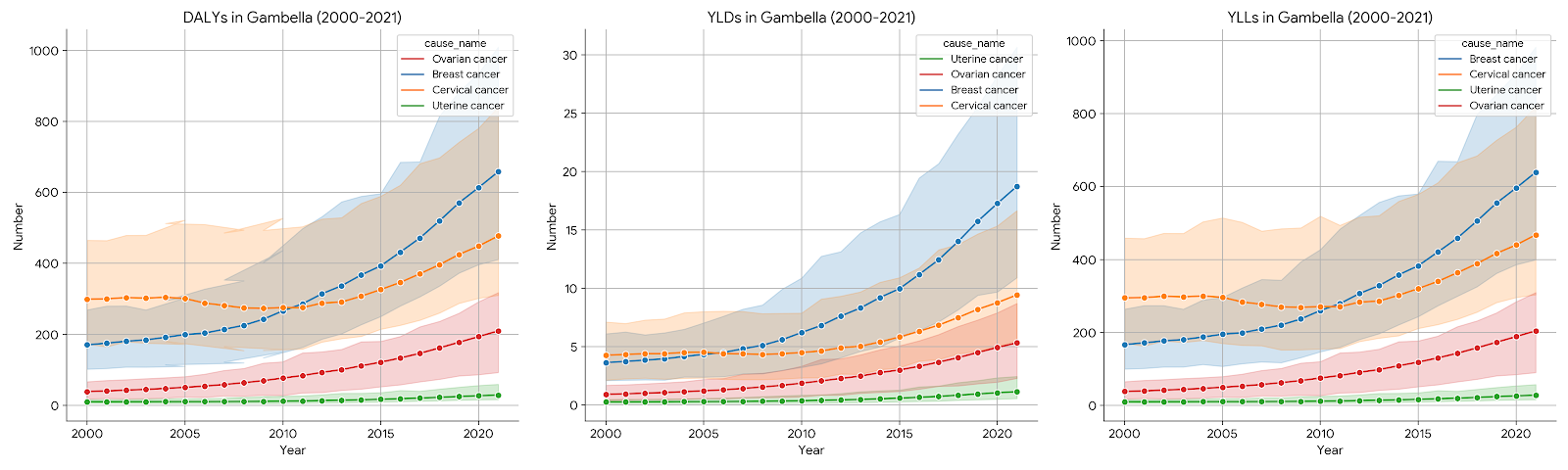


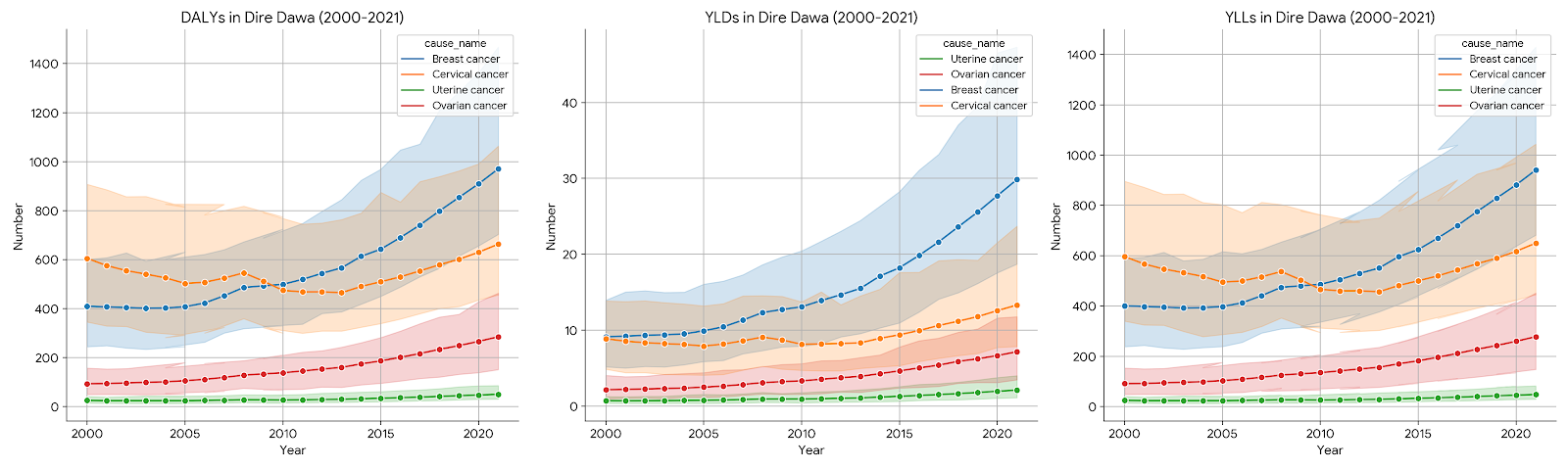


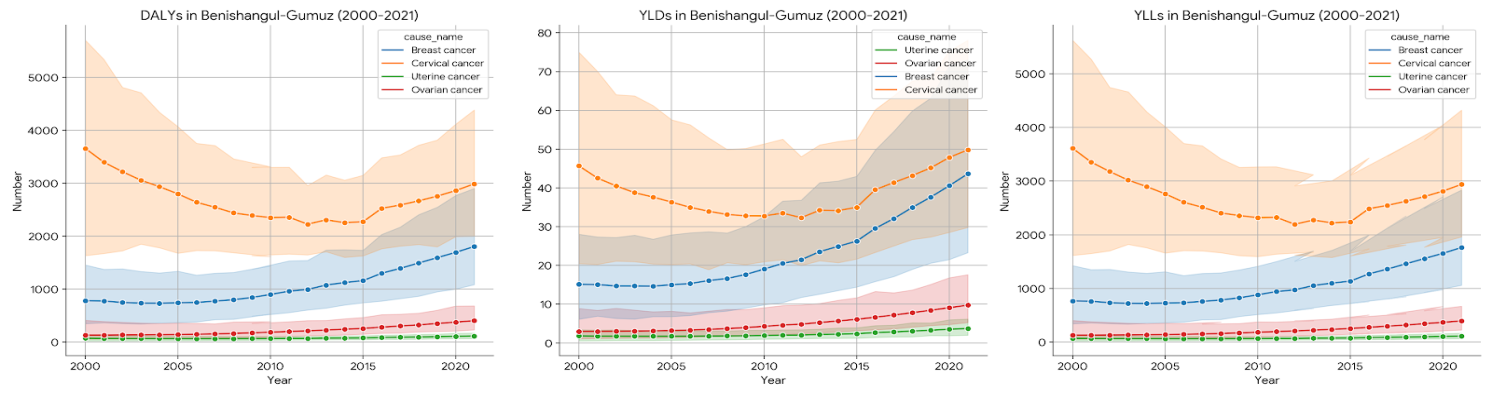


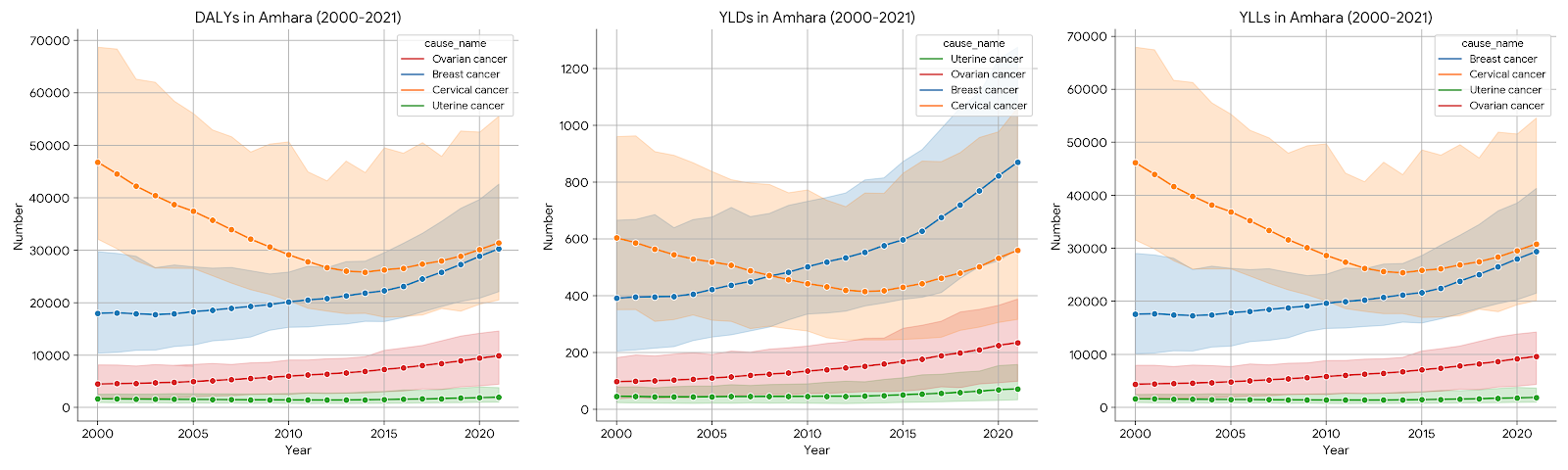


Supplemental fig 2: the Sub-national DALYs, YLD, and YLLs trend of Female specific cancer in Ethiopia (2000-2021)
