# Supplementary material for: The rising burden of female cancer in Ethiopia (2000–2021) and projections to 2040: Insights from the global burden of disease study
Source: PLoS One. 2025 Oct 7;20(10):e0333787. doi: 10.1371/journal.pone.0333787 (PMC12503343; doi:10.1371/journal.pone.0333787)
Supplement: Attachment — Submitted filename: PONE-D-25-35361 - reviewed.pdf [file pone.0333787.s002.pdf]

PLOS ONE

The Rising Burden of Female Cancers in Ethiopia: A 21-years National and Sub-national Analysis of Breast, Cervical, Ovarian and Uterine Cancers (2000–2021) with Projections to 2040 – Findings from the Global Burden of Disease Study

--Manuscript Draft--

**Manuscript Number:**	PONE-D-25-35361
**Article Type:**	Research Article
**Full Title:**	The Rising Burden of Female Cancers in Ethiopia: A 21-years National and Sub-national Analysis of Breast, Cervical, Ovarian and Uterine Cancers (2000–2021) with Projections to 2040 – Findings from the Global Burden of Disease Study
**Short Title:**	The Rising Burden of Female Cancers in Ethiopia(2000-2021): Findings from the Global Burden of Disease studies
**Corresponding Author:**	Molalign Adisu Aligaz<br>Woldia University<br>Woldia, Ethiopia, Woldia ETHIOPIA
**Keywords:**	Female breast cancer; cervical cancer; ovarian cancer; uterine cancer; cancer burden; trend; projection; Ethiopia
**Abstract:**	Introduction<br><br>Cancer poses a significant global health challenge, particularly in low- and middle-income countries like Ethiopia, where it contributes to a growing double burden of disease alongside infectious diseases. Female cancers, including breast, cervical, ovarian, and uterine cancers, represent a severe public health crisis, imposing substantial socio-economic burdens. Despite this escalating threat, detailed and geographically disaggregated data on these cancers remain scarce, hindering effective policy and intervention. Addressing this critical evidence gap, our study utilizes the Global Burden of Disease methodology to provide a comprehensive 21-year review (2000-2021) of national and sub-national trends and associated risk factors for these cancers in Ethiopia, with projections to 2040, aiming to inform targeted cancer control efforts and strengthen health infrastructure.<br><br>Methods<br><br>A secondary data analysis approach was applied on the Global burden of disease 2021 study. National and sub-national prevalence, incidence, mortality, disability-adjusted life years, year of life lost, years lived with disability and risk factors for breast, cervical ovarian and uterine cancers in Ethiopia from 2000 to 2021. An Autoregressive Integrated Moving Average (ARIMA) model was employed for projecting epidemiological trajectories through 2040.All statistical analyses and data visualization were performed using python.<br><br>Results<br><br>Between 2000 and 2021, Ethiopia experienced a substantial increase in the national prevalence and incidence of breast cancer (169.82% prevalence, 148.98% incidence) and a spectacular rise in ovarian cancer (197.18% prevalence, 160.97% incidence). While cervical cancer DALYs decreased (-15.22%), its YLDs increased, suggesting improved survival but rising long-term disability. Ovarian and breast cancer experienced increasing DALYs/YLDs at all ages, most notably in reproductive age (15-49) and elderly (75+) women for breast and young women for ovarian cancer. At the sub-national level, densely populated regions like Oromia and Amhara, as well as urban centers like Addis Ababa, experienced significant increases. Projections to 2040 indicate a continuation of increasing rates of incidence for all cancers in females, most notably ovarian and breast cancer, with an increasing burden in the future.<br><br>Conclusions<br><br>Female breast and ovarian cancer were escalating public health crisis in Ethiopia, as

	showed by significant increments in prevalence incidence, disability-adjusted life years, and especially years lived with disability in all age groups and regions with reproductive age women disproportionately affected by ovarian cancer burden. Although the burden of cervical cancer is steadily decreasing, the growing number of YLDs indicates an increasing need for long-term care. The increasing projected incidence for all female cancers suggests an urgent need for preventive and tailored interventions such as improving early diagnosis of the cases, age-appropriate screening, and cancer care services especially among high-burden urban and rural areas to minimize the potential devastating effects on Ethiopian women's health.
**Order of Authors:**	Molalign Adisu Aligaz
	Tesfaye Engdaw Habtie
	Tegene Atamenta Kitaw
	Abraham Dessie Gessesse
	Bogale Molla Woreta
	Yabibal Asfaw Derso
	Alemu Birara Zemariam
**Additional Information:**	
**Question**	**Response**
**Financial Disclosure**<br><br>Enter a financial disclosure statement that describes the sources of funding for the work included in this submission. Review the submission guidelines for detailed requirements. View published research articles from *PLOS ONE* for specific examples.<br><br>This statement is required for submission and **will appear in the published article** if the submission is accepted. Please make sure it is accurate.<br><br>**Funded studies**<br>Enter a statement with the following details:<br>• Initials of the authors who received each award<br>• Grant numbers awarded to each author<br>• The full name of each funder<br>• URL of each funder website<br>• Did the sponsors or funders play any role in the study design, data collection and analysis, decision to publish, or preparation of the manuscript?<br><br>Did you receive funding for this work?	The author(s) received no specific funding for this work.
**Competing Interests**	The authors have declared that no competing interests exist.

Use the instructions below to enter a competing interest statement for this submission. On behalf of all authors, disclose any competing interests that could be perceived to bias this work—acknowledging all financial support and any other relevant financial or non-financial competing interests.<br><br>This statement is required for submission and **will appear in the published article** if the submission is accepted. Please make sure it is accurate and that any funding sources listed in your Funding Information later in the submission form are also declared in your Financial Disclosure statement.<br><br>View published research articles from *PLOS ONE* for specific examples.<br><br>**NO authors have competing interests**<br><br>Enter: *The authors have declared that no competing interests exist.*<br><br>**Authors with competing interests**<br><br>Enter competing interest details beginning with this statement:<br><br>*I have read the journal's policy and the authors of this manuscript have the following competing interests: [insert competing interests here]*<br><br>* typeset	
**Ethics Statement**<br><br>Enter an ethics statement for this submission. This statement is required if the study involved:<br><br>• Human participants<br>• Human specimens or tissue<br>• Vertebrate animals or cephalopods<br>• Vertebrate embryos or tissues	N/A

• Field research<br><br>Write "N/A" if the submission does not require an ethics statement.<br><br>General guidance is provided below. Consult the submission guidelines for detailed instructions. **Make sure that all information entered here is included in the Methods section of the manuscript.**	

Powered by Editorial Manager® and ProduXion Manager® from Aries Systems Corporation

**Format for specific study types**<br><br>**Human Subject Research (involving human participants and/or tissue)**<br>• Give the name of the institutional review board or ethics committee that approved the study<br>• Include the approval number and/or a statement indicating approval of this research<br>• Indicate the form of consent obtained (written/oral) or the reason that consent was not obtained (e.g. the data were analyzed anonymously)<br><br>**Animal Research (involving vertebrate animals, embryos or tissues)**<br>• Provide the name of the Institutional Animal Care and Use Committee (IACUC) or other relevant ethics board that reviewed the study protocol, and indicate whether they approved this research or granted a formal waiver of ethical approval<br>• Include an approval number if one was obtained<br>• If the study involved *non-human primates*, add *additional details* about animal welfare and steps taken to ameliorate suffering<br>• If anesthesia, euthanasia, or any kind of animal sacrifice is part of the study, include briefly which substances and/or methods were applied<br><br>**Field Research**<br><br>Include the following details if this study involves the collection of plant, animal, or other materials from a natural setting:<br>• Field permit number<br>• Name of the institution or relevant body that granted permission	
**Data Availability**<br><br>Authors are required to make all data underlying the findings described fully available, without restriction, and from the time of publication. PLOS allows rare exceptions to address legal and ethical concerns. See the PLOS Data Policy and FAQ for detailed information.	Yes - all data are fully available without restriction

A Data Availability Statement describing where the data can be found is required at submission. Your answers to this question constitute the Data Availability Statement and **will be published in the article**, if accepted.<br><br>**Important:** Stating 'data available on request from the author' is not sufficient. If your data are only available upon request, select 'No' for the first question and explain your exceptional situation in the text box.<br><br>Do the authors confirm that all data underlying the findings described in their manuscript are fully available without restriction?	
**Describe where the data may be found in full sentences. If you are copying our sample text, replace any instances of XXX with the appropriate details.**<br><br>• If the data are **held or will be held in a public repository**, include URLs, accession numbers or DOIs. If this information will only be available after acceptance, indicate this by ticking the box below. For example: *All XXX files are available from the XXX database (accession number(s) XXX, XXX.).*<br>• If the data are all contained **within the manuscript and/or Supporting Information files**, enter the following: *All relevant data are within the manuscript and its Supporting Information files.*<br>• If neither of these applies but you are able to provide **details of access elsewhere**, with or without limitations, please do so. For example:<br><br>*Data cannot be shared publicly because of [XXX]. Data are available from the XXX Institutional Data Access / Ethics Committee (contact via XXX) for researchers who meet the criteria for access to confidential data.*<br><br>*The data underlying the results presented in the study are available from (include the name of the third party*	The data used in this study accessed from to the GBD 2021 official website is available at https://ghdx.healthdata.org/gbd-2021

and contact information or URL).<br>• This text is appropriate if the data are owned by a third party and authors do not have permission to share the data.<br><br>* typeset	
Additional data availability information:	

Powered by Editorial Manager® and ProduXion Manager® from Aries Systems Corporation

Manuscript

Click here to access/download;Manuscript;Manuscript FBCOUC.docx

The Rising Burden of Female Cancers in Ethiopia: A 21-years National and Sub-national Analysis of Breast, Cervical, Ovarian and Uterine Cancers (2000–2021) with Projections to 2040 – Findings from the Global Burden of Disease Study

Molalign Aligaz Adisu[*1], Tesfaye Engdaw Habtie[2], Tegene Atamenta Kitaw2, Abraham Dessie Gessesse[1], Bogale Molla Woreta[2], Yabibal Asfaw Derso[2], Alemu Birara Zemariam[1]

[1]Department of Pediatrics and child health Nursing, College of Health science, Woldia University, Woldia, Ethiopia

[2]Department of Nursing, College of Health science, Woldia University, Woldia, Ethiopia

[*]Corresponding Author: Molalign Aligaz Adisu

Email: molalignaligaz@gmail.com

molalign.a@wldu.edu.et

Abstract

Introduction: Cancer poses a significant global health challenge, particularly in low- and middle-income countries like Ethiopia, where it contributes to a growing double burden of disease alongside infectious diseases. Female cancers, including breast, cervical, ovarian, and uterine cancers, represent a severe public health crisis, imposing substantial socio-economic burdens. Despite this escalating threat, detailed and geographically disaggregated data on these cancers remain scarce, hindering effective policy and intervention. Addressing this critical evidence gap, our study utilizes the Global Burden of Disease methodology to provide a comprehensive 21-year review (2000-2021) of national and sub-national trends and associated risk factors for these cancers in Ethiopia, with projections to 2040, aiming to inform targeted cancer control efforts and strengthen health infrastructure.

Methods: A secondary data analysis approach was applied on the Global burden of disease 2021 study. National and sub-national prevalence, incidence, mortality, disability-adjusted life years, year of life lost, years lived with disability and risk factors for breast, cervical ovarian and uterine cancers in Ethiopia from 2000 to 2021. An Autoregressive Integrated Moving Average (ARIMA) model was employed for projecting epidemiological trajectories through 2040.All statistical analyses and data visualization were performed using python.

Results: Between 2000 and 2021, Ethiopia experienced a substantial increase in the national prevalence and incidence of breast cancer (169.82% prevalence, 148.98% incidence) and a spectacular rise in ovarian cancer (197.18% prevalence, 160.97% incidence). While cervical cancer DALYs decreased (-15.22%), its YLDs increased, suggesting improved survival but rising long-term disability. Ovarian and breast cancer experienced increasing DALYs/YLDs at all ages, most notably in reproductive age (15-49) and elderly (75+) women for breast and young women for ovarian cancer. At the sub-national level, densely populated regions like Oromia and Amhara, as well as urban centers like Addis Ababa, experienced significant increases. Projections to 2040 indicate a continuation of increasing rates of incidence for all cancers in females, most notably ovarian and breast cancer, with an increasing burden in the future.

Conclusions: Female breast and ovarian cancer were escalating public health crisis in Ethiopia, as showed by significant increments in prevalence incidence, disability-adjusted life years, and especially years lived with disability in all age groups and regions with reproductive age women disproportionately affected by ovarian cancer burden. Although the burden of cervical cancer is

steadily decreasing, the growing number of YLDs indicates an increasing need for long-term care. The increasing projected incidence for all female cancers suggests an urgent need for preventive and tailored interventions such as improving early diagnosis of the cases, age-appropriate screening, and cancer care services especially among high-burden urban and rural areas to minimize the potential devastating effects on Ethiopian women's health.

Keywords: Female breast cancer; cervical cancer; ovarian cancer; uterine cancer; cancer burden; trend; projection; Ethiopia.

Introduction

Cancer remains a significant global health problem, the second leading cause of death worldwide, disproportionately impacting low- and middle-income countries where over 70% of cancer deaths occur (1, 2). Increasingly, the burden is most attributable to demographic change, epidemiologic transition, and continued inequities in access and quality of care (2, 3). In Sub-Saharan African countries, the convergence of a growing and aging population, rapid urbanization, and lifestyle changes is driving a doubling of new cancer cases further straining already compromised health systems (1, 4).

Ethiopia, the second-largest population in Africa, is in a health transition crossroads. While tremendous progress has been made against infectious diseases, the country now grapples with a mounting double burden of disease, marked by a rapid increase in non-communicable diseases (NCDs), like cancers (5). Female cancers, such as breast, cervical, ovarian, and uterine cancers, are a severe public health crisis in this context (6, 7). These cancers collectively impose significant socio-economic burdens, resulting in premature mortality, impaired quality of life, and catastrophic healthcare expenditures on individuals and families, driving existing inequalities(8, 9).

Despite the evident and growing threat, comprehensive, long-term, and granular data on the specific trajectory and geographic distribution of these critical female cancers in Ethiopia remain notably scarce. Existing national cancer registries are nascent or incomplete, and population-based incidence and mortality statistics are often limited, fragmented, or derived from disparate methodologies. This is a key data gap that considerably constrains policymakers, public health experts, and physicians' ability to accurately estimate the real size of the issue, identify high-

burden areas, project future needs, and implement evidence-based national and sub-national cancer control policies. A robust understanding of disease trends over time and by administrative level is essential to effective resource planning, targeted intervention (e.g., screening, immunization, early treatment, and diagnosis programs), and the creation of responsive health policy(5, 10).

Based on the extensive and standardize methodology of the Global Burden of Disease (GBD) Study, this article presents a comprehensive 21-year national and sub-national incidence, mortality, and disability-adjusted life years (DALYs), year of life lost (YLL), years lived with disability (YLD), and risk factor review of breast, cervical, ovarian, and uterine cancers in Ethiopia from 2000 to 2021. It also provides important projections of these burdens through 2040. Quantifying systematically the shifting epidemiology of these cancers and projecting their future trend, this study aims to fill an important evidence gap. The findings will provide invaluable information to the Ethiopian Ministry of Health, regional health bureaus, and global partners, allowing cancer control efforts to be targeted, health infrastructure to be strengthened, and ultimately the devastating impact of female cancers on the health status and quality of life of women of Ethiopia to be minimized. This research is a significant contribution to global health literature by offering a model to understand and address the rising cancer burden in poor resource settings.

Methods

Data Source and Scope

This study employed estimates of the Global Burden of Disease (GBD) Study from 2000 to 2021 to compare the burden, incidence, prevalence, mortality, years of life lost (YLLs), years lived with disability (YLDs), and disability-adjusted life years (DALYs) for breast, cervical, ovarian, and uterine cancers in Ethiopia. The findings are expressed in terms of rates per 100,000 populations with 95% uncertainty intervals (UIs). Data are accessed via Global Health Data Exchange (GHDx) at Institute for Health Metrics and Evaluation (IHME), University of Washington (http://ghdx.healthdata.org/gbd-results-tool)

The GBD represents a systematic effort to quantify health loss due to diseases and injuries across various demographics and geographic locations. The data was gathered from a variety of

sources, including national cancer registries, death records, and health care utilization data. Particular mortality rates and disease burdens were estimated with the Cause of Death Ensemble model and spatiotemporal Gaussian process regression.

A comprehensive analysis was conducted to assess national and sub-national patterns of cancer incidence and burden between 2000 and 2021. Specific search criteria included cancer type (breast, cervical, ovarian, and uterine), measure (incidence and mortality), metric (count and rate), place (Ethiopia), and sub-nations of the nine regions and two city administrations, and age group (all ages, age-standardized, and specific age groups). This approach facilitated the detection of high-burden sites and informed targeted public health interventions.

Statistical analysis

Burden of female cancer was quantified as prevalence, incidence, mortality, YLLs, YLDs, and DALYs. Age-standardized rates (ASRs) for specific age groups, along with estimated values and 95% uncertainty intervals (UIs), were sourced from GBD 2021. ASR was determined according to the following formula:

$$ASR = \frac{\sum_{i=1}^{N} a_i w_i}{\sum_{i=1}^{N} w_i} \times 100,000$$

= Where a_i represents the age-specific rate for the *ith* age group and w_i denotes the number of individuals (or the weight) in the same age group within the GBD 2021 standard population. *N* is the number of age groups.

To analyze trends in cancer incidence and mortality, percentage changes were calculated by comparing counts from 2000 to 2021 using the formula:

$$Percentage\ Change = \frac{(Y_{2021} - Y_{2000})}{Y_{2000}} \times 100\%$$

= where Y refers to total incident cases or total deaths.

Statistical analyses for trend and Autoregressive Integrated Moving Average (ARIMA) projection to 2040 were conducted using Python (version 3.12), using libraries *pandas* for data manipulation and *statsmodels* for ARIMA modeling. Data visualization, including generation of

plots illustrating trends and uncertainty intervals were done using the *matplotlib* and *seaborn* libraries.

This method aims to lay the foundation of a comprehensive framework of knowledge of the trends and projections of Ethiopian female cancers so as to eventually inform public health policy and resource allocation interventions appropriately.

Ethics Statement

For GBD studies, the Institutional Review Board of the University of Washington reviewed and approved a waiver of informed consent (https://www.healthdata.org/research-analysis/gbd).

Results

The national prevalence, incidence and death Trends of Female breast, cervical, ovarian and uterine cancer in Ethiopia (2000-2021)

In Ethiopia, the number of breast cancer increased significantly from 20,149 (95% UI: 15,063–27,411) cases in 2000 to 54,367 (95% UI: 44,141–66,730) cases in 2021, an increase of 169.82%. Similarly, the incidence of breast cancer increased from 2,935 (95% UI: 2,064–4,154) to 7,308 (95% UI: 5,794–9,199) new cases, an increase of 148.98%. For cervical cancer, prevalence rose from 24,610 (95% UI: 18,981–35,190) to 32,665 (95% UI: 23,296–49,268) cases (32.73% rise), while incidence rose more modestly from 7,573 (95% UI: 5,846–10,736) to 7,884 (95% UI: 5,759–11,765) new cases (4.10% rise). Cancer of the ovary had a spectacular increase in prevalence, from 2,793 (95% UI: 1,467–4,926) to 8,300 (95% UI: 4,214–11,948) cases (197.18% increase), and incidence, from 787 (95% UI: 394–1,391) to 2,054 (95% UI: 1,034–2,929) new cases (160.97% increase). Lastly, uterine cancer incidence nearly doubled, from 2,037 (95% UI: 1,289–2,881) to 4,290 (95% UI: 2,660–7,363) cases (110.61% increase), with incidence increasing from 357 (95% UI: 230–511) to 669 (95% UI: 422–1,126) new cases (87.11% increase) **(Figure 1).** These national estimates point to a widespread and increasing cancer burden on the Ethiopian health system.

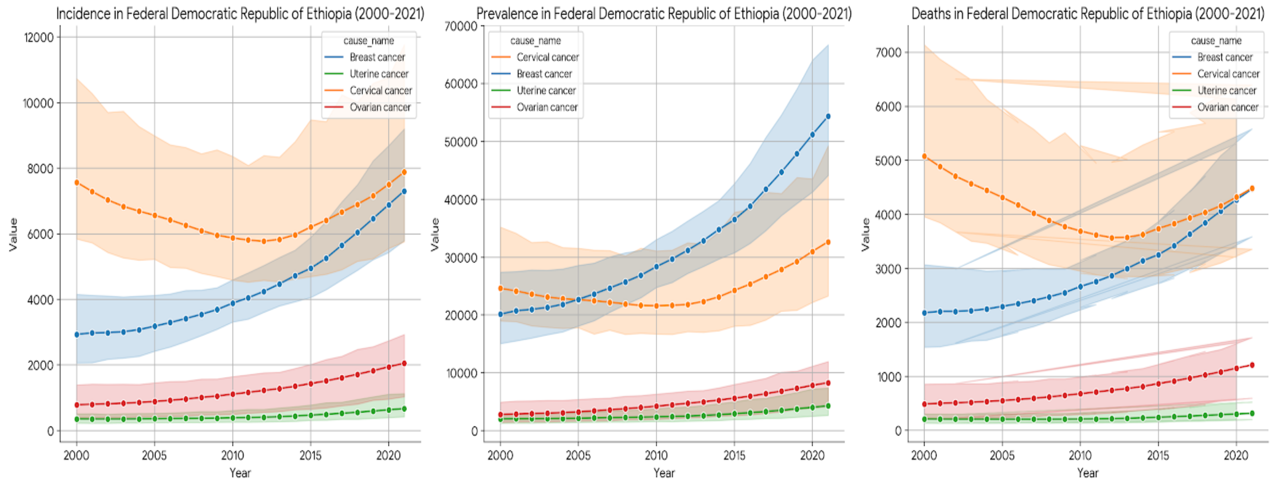

Figure 1: prevalence, incidence and mortality rate of beast, cervical, ovarian and uterine cancer in Ethiopia (2000-2021)

Sub-National prevalence, Incidence and mortality trends of beast, cervical, ovarian and uterine cancer in Ethiopia (2000-2021)

Breast Cancer

The prevalence of breast cancer increased significantly in areas such as Oromia, rising from 8,903 (95% UI: 6,654–12,056) cases in 2000 to 22,810 (95% UI: 18,527–28,145) cases in 2021. Similarly, the number of new cases increased from 1,323 (95% UI: 928–1,865) to 3,243 (95% UI: 2,569–4,074). Amhara also demonstrated a significant increase, with incidence rising from 622 (95% UI: 435–886) to 1,570 (95% UI: 1,244–1,973) new cases and prevalence rising from 4,374 (95% UI: 3,275–5,947) to 11,855 (95% UI: 9,678–14,463) cases. Despite having a smaller geographic area, Addis Ababa showed a considerable burden, with incidence rising from 272 (95% UI: 191–386) to 665 (95% UI: 529–834) new cases and prevalence rising from 1,847 (95% UI: 1,380–2,504) to 4,964 (95% UI: 4,008–6,128) cases **(Figure 2).** This suggests that urban centers like Addis Ababa face a concentrated challenge, while rural areas, with their larger populations, contribute significantly to the overall burden.

Cervical Cancer

Cervical cancer trends also differed by region. In Oromia, incidence showed a slight increase from 3,514 (95% UI: 2,698–5,005) to 3,576 (95% UI: 2,586–5,321) new cases, while prevalence rose from 10,753 (95% UI: 8,290–15,361) to 12,027 (95% UI: 8,506–17,998) cases. Prevalence increased from 5,237 (95% UI: 4,028–7,515) to 6,108 (95% UI: 4,360–9,150) cases in Amhara,

while incidence increased from 1,540 (95% UI: 1,180–2,185) to 1,575 (95% UI: 1,146–2,357) new cases. Prevalence increased from 4,394 (95% UI: 3,425–6,165) to 5,532 (95% UI: 3,923–8,429) cases in SNNPR (Southern Nations, Nationalities, and Peoples' Region), while incidence increased from 1,249 (95% UI: 981–1,732) to 1,304 (95% UI: 955–1,911) new cases **(Figure 2).** The continuous burden suggests that screening and prevention efforts need to be improved. These regional patterns, especially the slower growth in incidence compared to prevalence in some places, may imply advances in diagnostic capacities or longer survival among prevalent patients.

Ovarian Cancer

Oromia once again had the highest burden of ovarian cancer, with incidence rising from 330 (95% UI: 164–581) to 828 (95% UI: 416–1,179) new cases and prevalence rising from 1,173 (95% UI: 615–2,060) to 3,363 (95% UI: 1,689–4,821). Amhara came next, with incidence increasing from 156 (95% UI: 78–275) to 433 (95% UI: 218–617) new cases and prevalence increasing from 552 (95% UI: 289–975) to 1,770 (95% UI: 897–2,537) cases. The percentage gains were significant even in smaller localities. The prevalence of ovarian cancer increased sharply throughout the country, for example, from 163 (95% UI: 86–287) to 488 (95% UI: 247–699) instances in Addis Ababa **(Figure 2).**

Uterine Cancer

Regional increases were also seen in uterine cancer. In Oromia, incidence went from 153 (95% UI: 99–219) to 266 (95% UI: 168–447) new cases, while prevalence went from 866 (95% UI: 549–1,223) to 1,707 (95% UI: 1,059–2,925) instances. Incidence increased from 72 (95% UI: 47–104) to 132 (95% UI: 83–222) new cases, while prevalence increased from 409 (95% UI: 258–579) to 842 (95% UI: 522–1,440) cases, according to Amhara **(Figure 2).** The substantial percentage increases for uterine cancer across regions suggest a growing health issue that requires attention, even though these absolute numbers are smaller than those for breast or cervical cancers.

The sub-national data shows that cancer cases are on the rise in Ethiopia, especially in regions with highest population like Oromia and Amhara. However, cities like Addis Ababa are struggling with a high number of cases too. This is probably because of population density and better access to tests. Understanding these details is really important for creating effective

strategies to raise awareness, improve early detection, and set up treatment centers throughout Ethiopia.

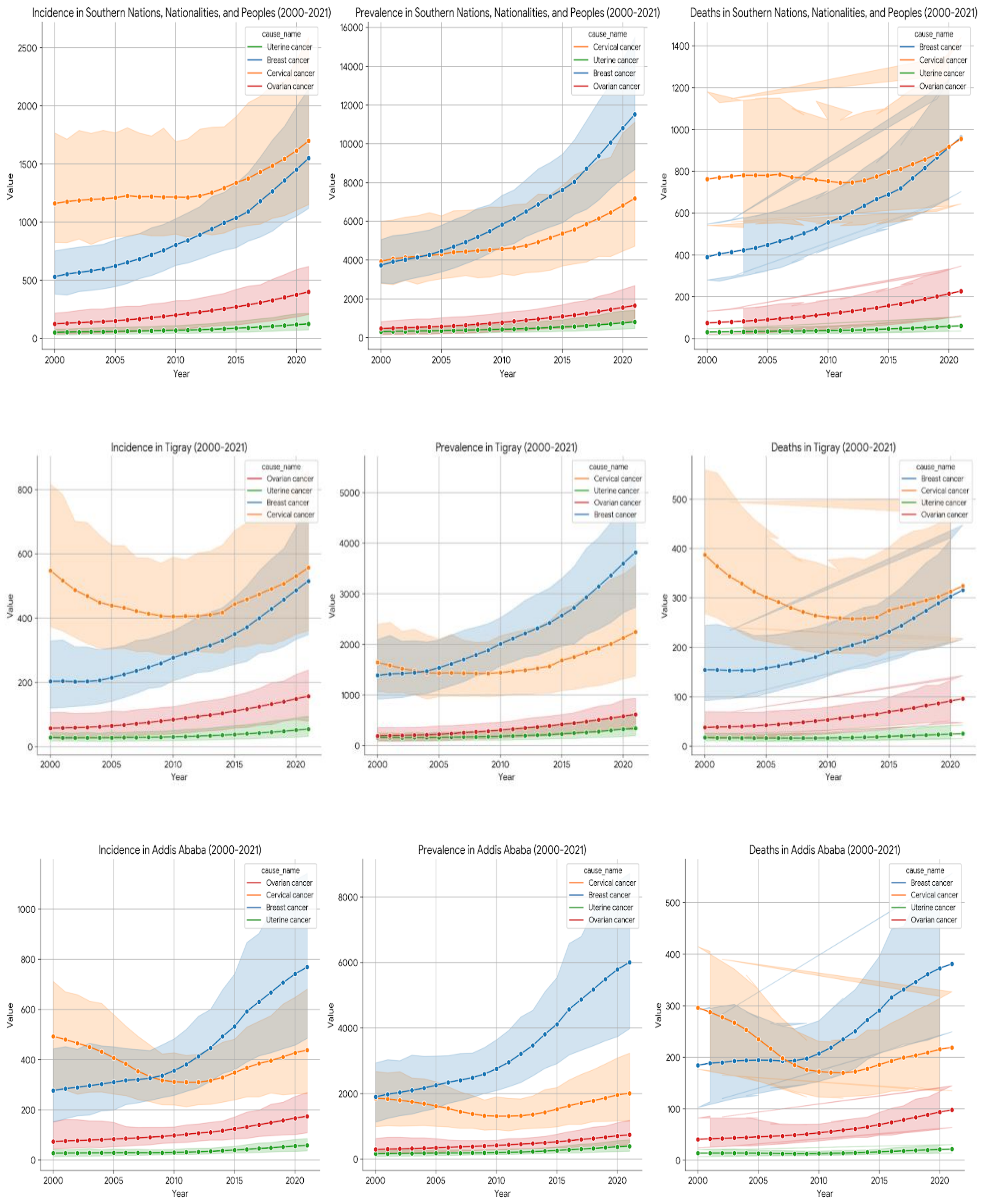

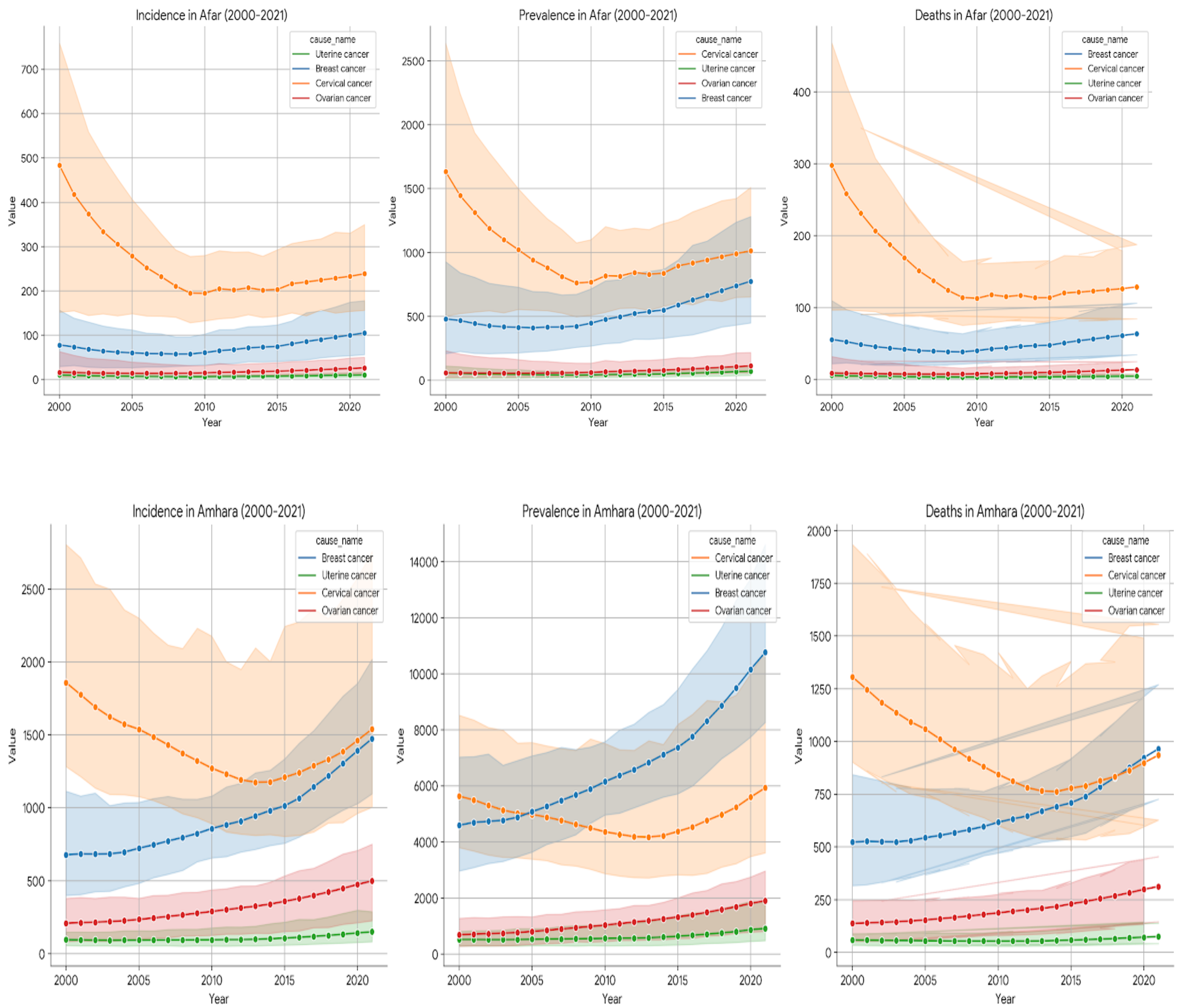

Incidence in Afar (2000-2021)
cause_name
Uterine cancer
Breast cancer
Cervical cancer
Ovarian cancer
Value
Year
Prevalence in Afar (2000-2021)
cause_name
Cervical cancer
Uterine cancer
Ovarian cancer
Breast cancer
Value
Year
Deaths in Afar (2000-2021)
cause_name
Breast cancer
Cervical cancer
Uterine cancer
Ovarian cancer
Value
Year
Incidence in Amhara (2000-2021)
cause_name
Breast cancer
Uterine cancer
Cervical cancer
Ovarian cancer
Value
Year
Prevalence in Amhara (2000-2021)
cause_name
Cervical cancer
Uterine cancer
Breast cancer
Ovarian cancer
Value
Year
Deaths in Amhara (2000-2021)
cause_name
Breast cancer
Cervical cancer
Uterine cancer
Ovarian cancer
Value
Year

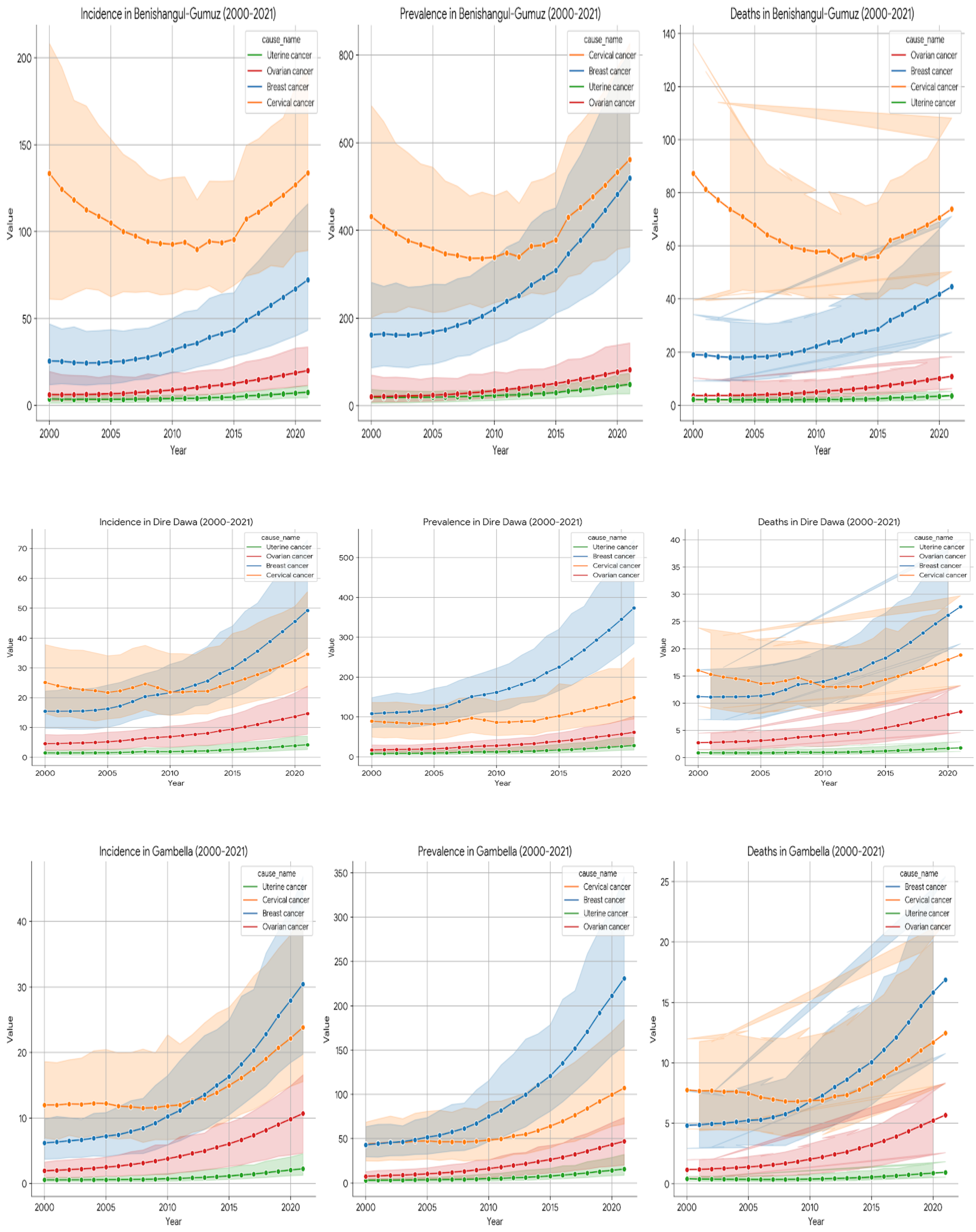

Incidence in Benishangul-Gumuz (2000-2021)
cause_name
Uterine cancer
Ovarian cancer
Breast cancer
Cervical cancer
Value
Year
Prevalence in Benishangul-Gumuz (2000-2021)
cause_name
Cervical cancer
Breast cancer
Uterine cancer
Ovarian cancer
Value
Year
Deaths in Benishangul-Gumuz (2000-2021)
cause_name
Ovarian cancer
Breast cancer
Cervical cancer
Uterine cancer
Value
Year
Incidence in Dire Dawa (2000-2021)
cause_name
Uterine cancer
Ovarian cancer
Breast cancer
Cervical cancer
Value
Year
Prevalence in Dire Dawa (2000-2021)
cause_name
Uterine cancer
Breast cancer
Cervical cancer
Ovarian cancer
Value
Year
Deaths in Dire Dawa (2000-2021)
cause_name
Uterine cancer
Ovarian cancer
Breast cancer
Cervical cancer
Value
Year
Incidence in Gambella (2000-2021)
cause_name
Uterine cancer
Cervical cancer
Breast cancer
Ovarian cancer
Value
Year
Prevalence in Gambella (2000-2021)
cause_name
Cervical cancer
Breast cancer
Uterine cancer
Ovarian cancer
Value
Year
Deaths in Gambella (2000-2021)
cause_name
Breast cancer
Cervical cancer
Uterine cancer
Ovarian cancer
Value
Year

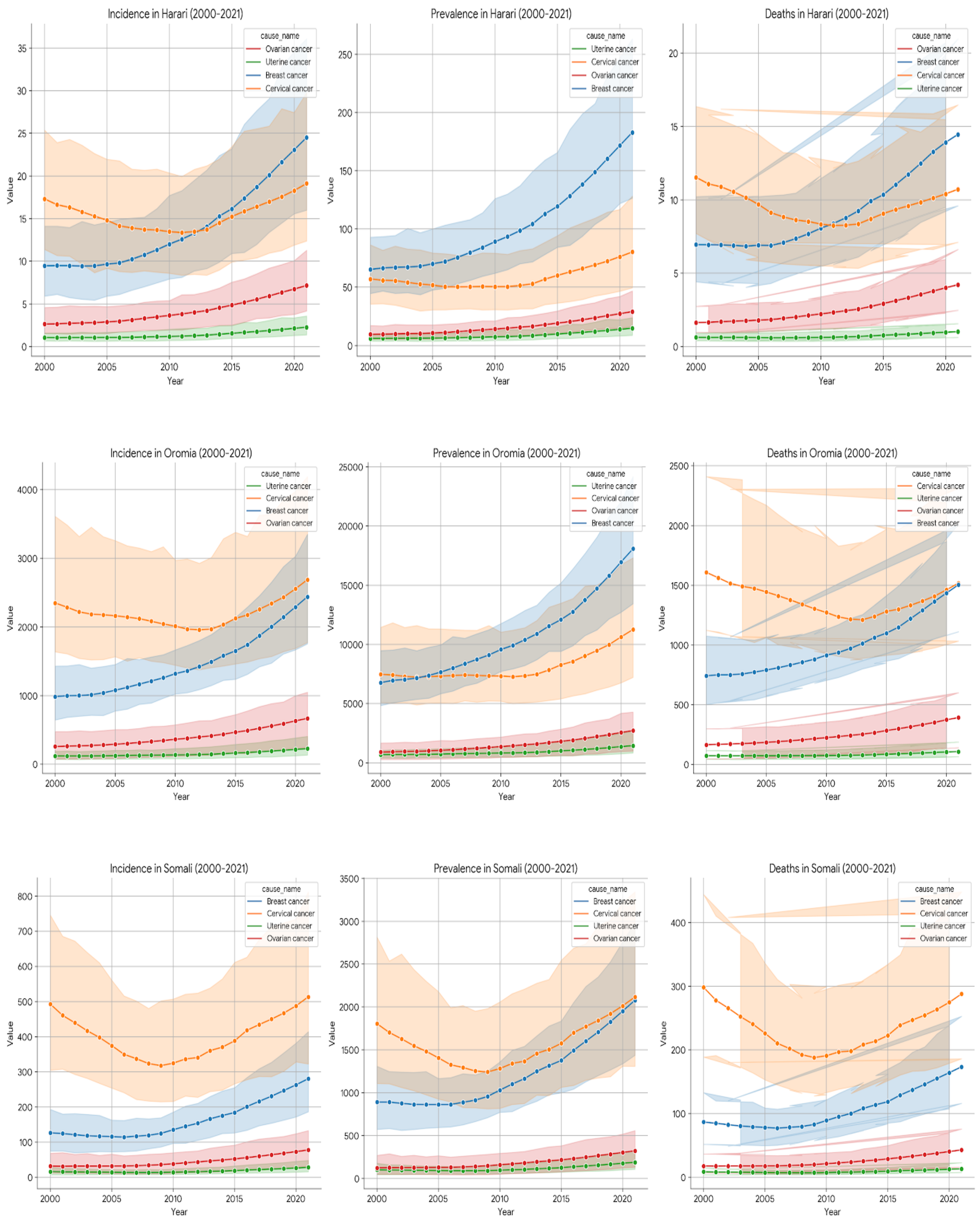

Incidence in Harari (2000-2021)
cause_name
Ovarian cancer
Uterine cancer
Breast cancer
Cervical cancer
Value
Year
Prevalence in Harari (2000-2021)
cause_name
Uterine cancer
Cervical cancer
Ovarian cancer
Breast cancer
Value
Year
Deaths in Harari (2000-2021)
cause_name
Ovarian cancer
Breast cancer
Cervical cancer
Uterine cancer
Value
Year
Incidence in Oromia (2000-2021)
cause_name
Uterine cancer
Cervical cancer
Breast cancer
Ovarian cancer
Value
Year
Prevalence in Oromia (2000-2021)
cause_name
Uterine cancer
Cervical cancer
Ovarian cancer
Breast cancer
Value
Year
Deaths in Oromia (2000-2021)
cause_name
Cervical cancer
Uterine cancer
Ovarian cancer
Breast cancer
Value
Year
Incidence in Somali (2000-2021)
cause_name
Breast cancer
Cervical cancer
Uterine cancer
Ovarian cancer
Value
Year
Prevalence in Somali (2000-2021)
cause_name
Breast cancer
Cervical cancer
Uterine cancer
Ovarian cancer
Value
Year
Deaths in Somali (2000-2021)
cause_name
Breast cancer
Cervical cancer
Uterine cancer
Ovarian cancer
Value
Year

Figure 2: Sub-national prevalence, incidence and mortality rate of beast, cervical, ovarian and uterine cancer in Ethiopia (2000-2021)

Deconstructing the total health loss: Disability-Adjusted Life Years, Years Lived with Disability, and Years of Life Lost for female breast, cervical, ovarian and uterine Cancers in Ethiopia (2000-2021)

Breast Cancer: a steadily increasing total burden with significant disability

The disability-adjusted life years (DALYs) attributable to breast cancer nationally increased from 80,211 (95% UI: 55,682–114,272) in 2000 to 155,931 (95% UI: 123,015–196,249) in 2021. This represents an absolute change of 75,719 and a significant 94.40% rise, highlighting the escalating comprehensive impact of breast cancer. Similarly, years lived with disability (YLDs) for breast cancer surged by an astounding 155.57%**,** from 1,723 (95% UI: 1,035–2,625) to 4,403 (95% UI: 2,897–6,261)**.** This indicates that a significant and disproportionately growing share of the breast cancer burden is attributed to living with the disease and its sequel, suggesting increased long-term care and supportive needs. Although Years of life lost (YLLs) increased by 93.06%**,** from 78,488 (95% UI: 54,439–111,774) to 151,527 (95% UI: 119,191–190,520). The faster rise in YLDs compared to YLLs suggests that while mortality increased, there's also a growing population of breast cancer patients living longer with some form of disability **(Figure 3**).

Cervical cancer: a complex picture of decreasing mortality but rising disability

The National cervical cancer DALYs decreased by -15.22%**,** from 191,988 (95% UI: 147,614–271,797) in 2000 to 162,776 (95% UI: 119,900–239,116) in 2021. Conversely, YLDs for cervical cancer increased by 15.78%**,** from 2,552 (95% UI: 1,736–3,661) to 2,955 (95% UI: 1,845–4,692)**.** On the other hand, The YLLs for cervical cancer showed a significant -15.63% decline, from 189,435 (95% UI: 145,754–268,405) to 159,820 (95% UI: 117,577–234,935) **(Figure 3**)**.** This divergent trend implies improved survival and reduced premature mortality (fewer YLLs), likely due to better screening or treatment, but a growing number of women are living with the long-term consequences of the disease (more YLDs).

Ovarian cancer: rapidly escalating disability burden

The ovarian cancer DALYs soared by 140.68%**,** from 16,798 (95% UI: 8,359–29,466) in 2000 to 40,430 (95% UI: 19,885–57,414) in 2021, indicating a very significant and growing overall burden. The increase in YLDs was even more pronounced, with a 165.57% rise from 371 (95% UI: 180–709) to 985 (95% UI: 497–1,531)**.** Additionally YLLs also increased substantially by 140.12%**,** from 16,427 (95% UI: 8,159–28,823) to 39,444 (95% UI: 19,445–56,039) **(Figure 3).** The higher percentage increase in YLDs compared to YLLs underscores the significant and worsening challenge in managing the long-term morbidity of ovarian cancer.

Uterine cancer: a growing disability contribution to the total burden

The uterine cancer DALYs increased by 39.28%**,** from 6,377 (95% UI: 4,088–9,202) in 2000 to 8,882 (95% UI: 5,579–15,240) in 2021, signifying a growing total burden. Moreover, YLDs for uterine cancer rose sharply by 89.82%**,** from 173 (95% UI: 96–282) to 328 (95% UI: 189–616)**.** Although, YLLs increased by 37.87%**,** from 6,204 (95% UI: 3,968–8,931) to 8,554 (95% UI: 5,385–14,586) **(Figure 3).** The YLDs increase outpacing DALYs and YLLs suggests that a greater proportion of the burden is now due to living with disability, emphasizing the need for comprehensive post-treatment care.

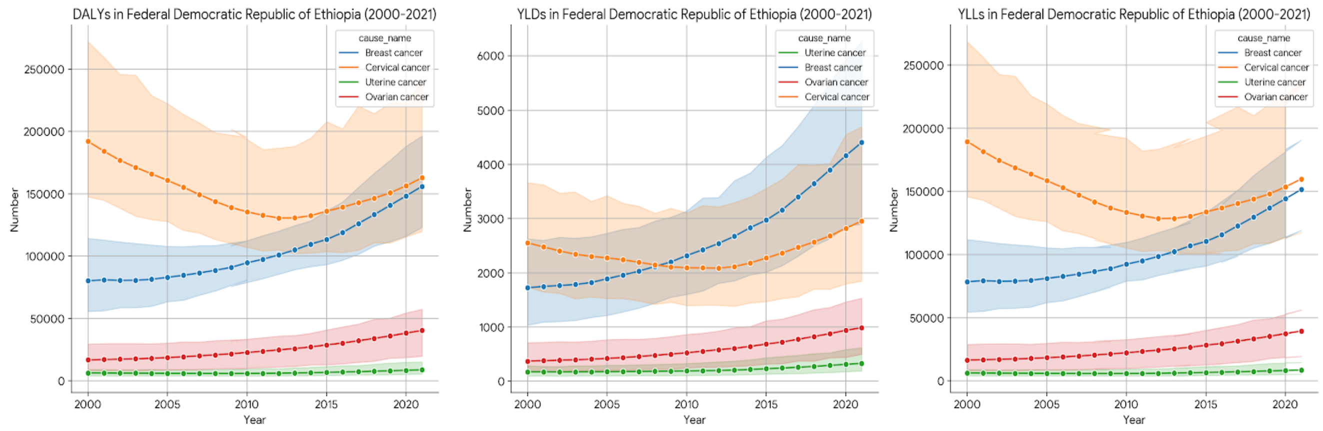

Figure 3: The DALYs, YLDs and YLLs trend of Female breast, cervical, ovarian and uterine cancer in Ethiopia (2000-2021)

Sub-National Burden Dynamics: Regional Nuances and Disparities

In Oromia, breast cancer has seen a significant rise in disease burden, with disability-adjusted life years (DALYs) increasing by 94.19%. Notably, years lived with disability (YLDs) surged by

155.38%, outpacing years of life lost (YLLs). Conversely, cervical cancer exhibited a decrease in DALYs by 7.83%, primarily due to an 8.34% reduction in YLLs, despite a 29.30% increase in YLDs. Amhara has similarly notable cancer burdens. Here, breast cancer DALYs rose by 68.44%, accompanied by a 122.45% increase in YLDs. In contrast, cervical cancer DALYs fell by 32.88%, with YLLs decreasing by 33.21% and YLDs showing a minor decline of 7.36%. Addis Ababa, though smaller in size, reported striking increases in YLDs for breast cancer (184.12%) and uterine cancer (114.05%). These figures may reflect enhanced diagnostic capabilities in urban settings, resulting in more identified cases that include disabilities. Furthermore, cervical cancer DALYs in Addis Ababa also dropped by 32.82%, driven by YLL declines. The Southern Nations, Nationalities, and Peoples (SNNP) region has experienced alarming increases across various cancer metrics, with ovarian cancer DALYs rising by 196.29%, YLLs by 195.67%, and YLDs escalating by 223.64% **(Figure 4).**

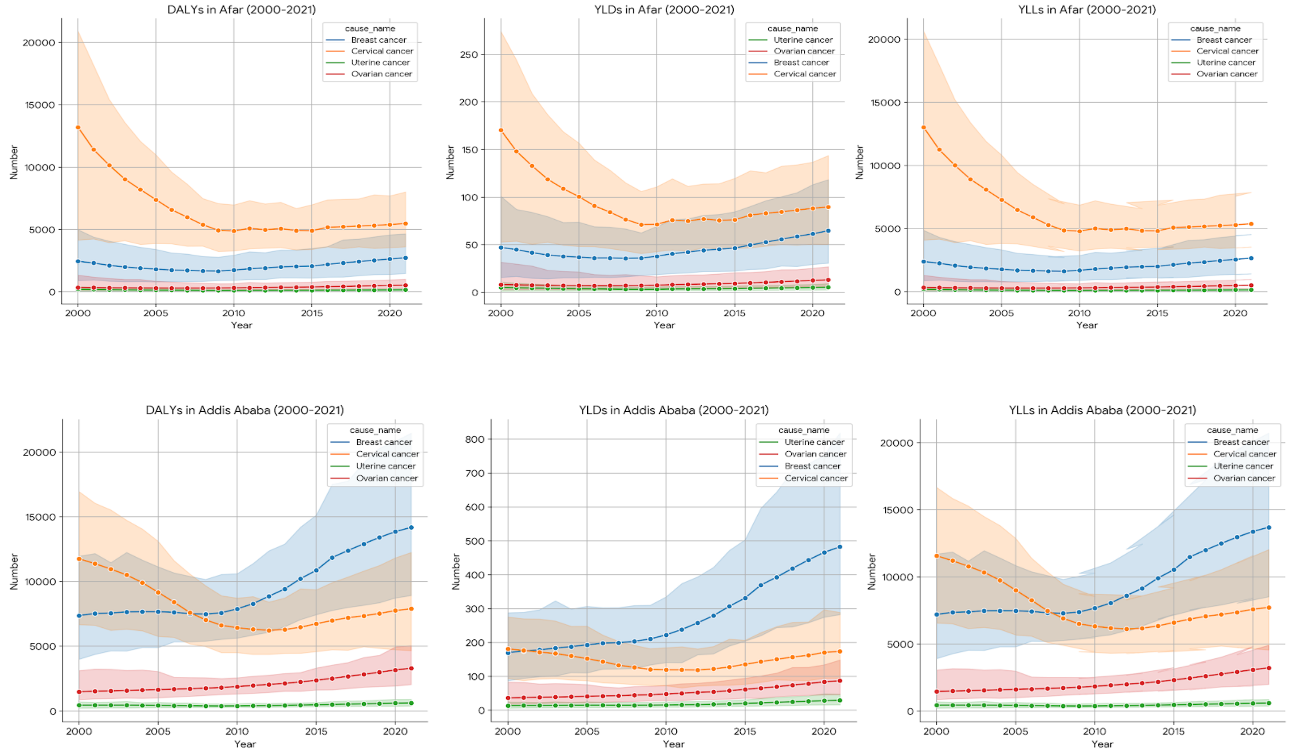

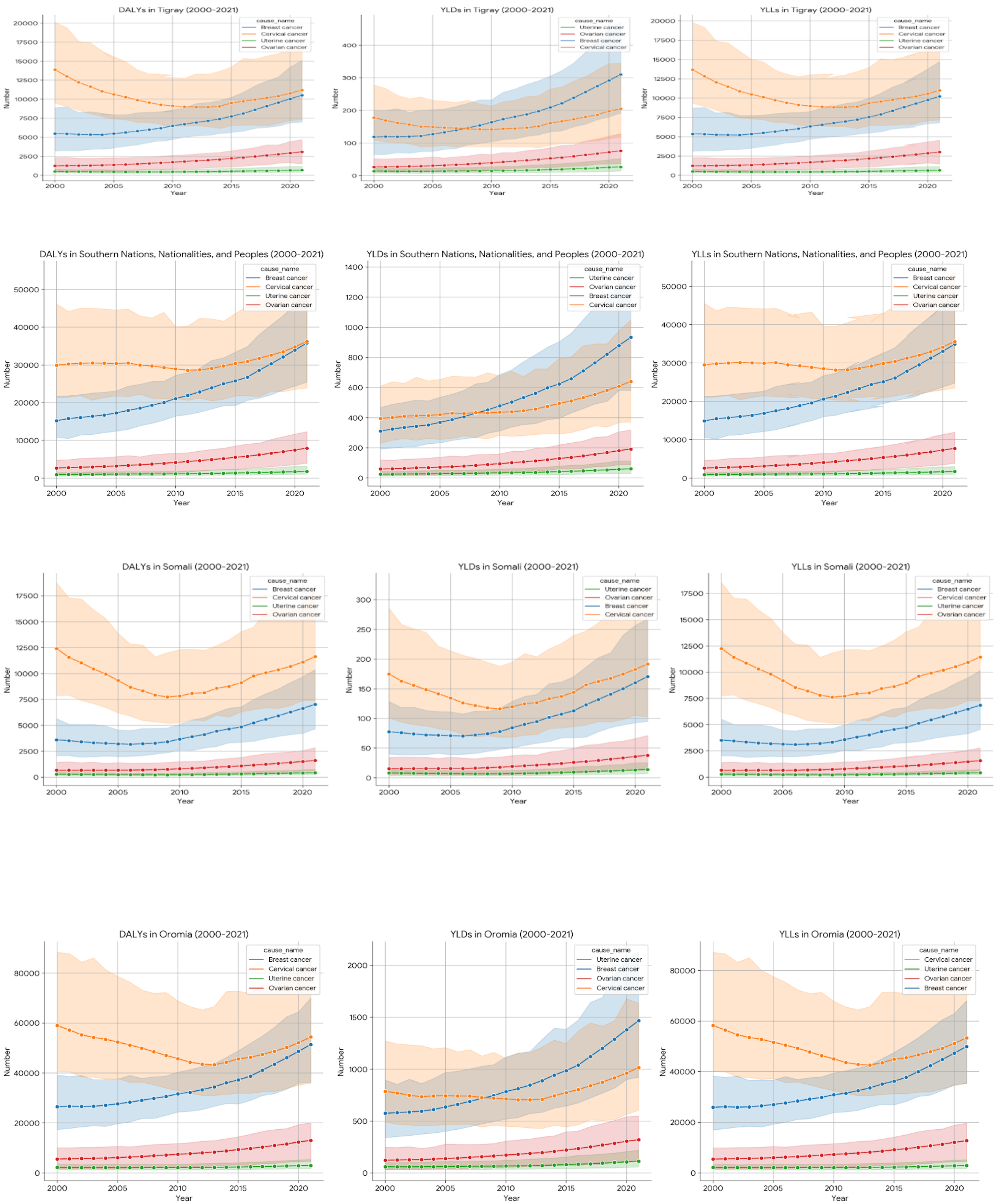
DALYs in Tigray (2000-2021)
YLDs in Tigray (2000-2021)
YLLs in Tigray (2000-2021)
DALYs in Southern Nations, Nationalities, and Peoples (2000-2021)
YLDs in Southern Nations, Nationalities, and Peoples (2000-2021)
YLLs in Southern Nations, Nationalities, and Peoples (2000-2021)
DALYs in Somali (2000-2021)
YLDs in Somali (2000-2021)
YLLs in Somali (2000-2021)
DALYs in Oromia (2000-2021)
YLDs in Oromia (2000-2021)
YLLs in Oromia (2000-2021)
cause_name
Breast cancer
Cervical cancer
Uterine cancer
Ovarian cancer
Number
Year

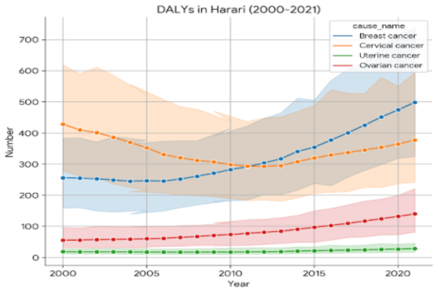
DALYs in Harari (2000-2021)
cause_name
Breast cancer
Cervical cancer
Uterine cancer
Ovarian cancer
Number
Year

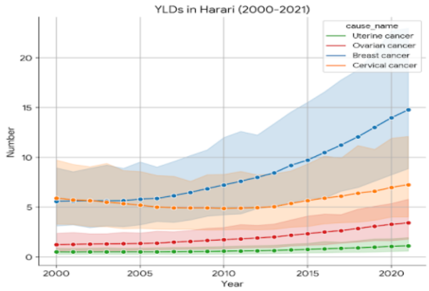
YLDs in Harari (2000-2021)
cause_name
Uterine cancer
Ovarian cancer
Breast cancer
Cervical cancer
Number
Year

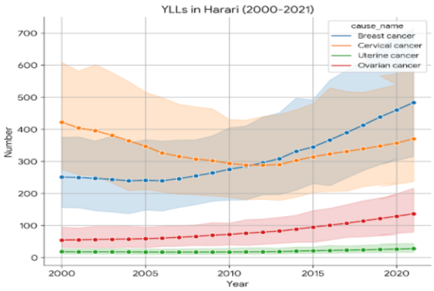
YLLs in Harari (2000-2021)
cause_name
Breast cancer
Cervical cancer
Uterine cancer
Ovarian cancer
Number
Year

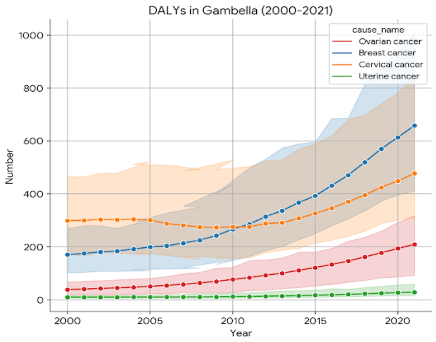
DALYs in Gambella (2000-2021)
cause_name
Ovarian cancer
Breast cancer
Cervical cancer
Uterine cancer
Number
Year

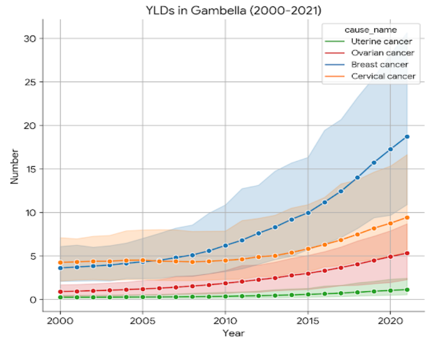
YLDs in Gambella (2000-2021)
cause_name
Uterine cancer
Ovarian cancer
Breast cancer
Cervical cancer
Number
Year

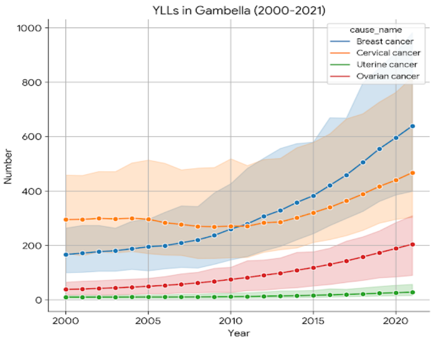
YLLs in Gambella (2000-2021)
cause_name
Breast cancer
Cervical cancer
Uterine cancer
Ovarian cancer
Number
Year

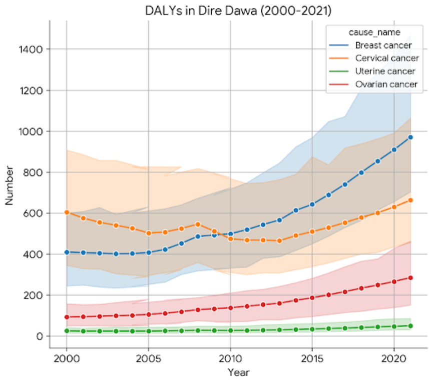
DALYs in Dire Dawa (2000-2021)
cause_name
Breast cancer
Cervical cancer
Uterine cancer
Ovarian cancer
Number
Year

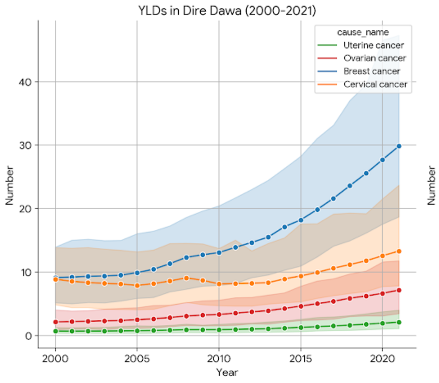
YLDs in Dire Dawa (2000-2021)
cause_name
Uterine cancer
Ovarian cancer
Breast cancer
Cervical cancer
Number
Year

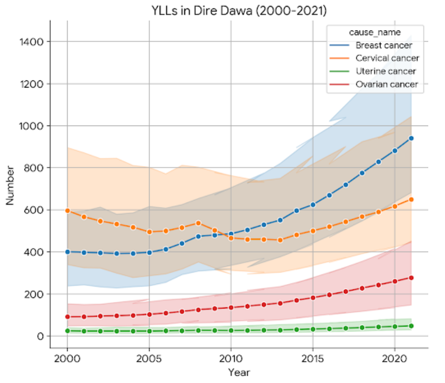
YLLs in Dire Dawa (2000-2021)
cause_name
Breast cancer
Cervical cancer
Uterine cancer
Ovarian cancer
Number
Year

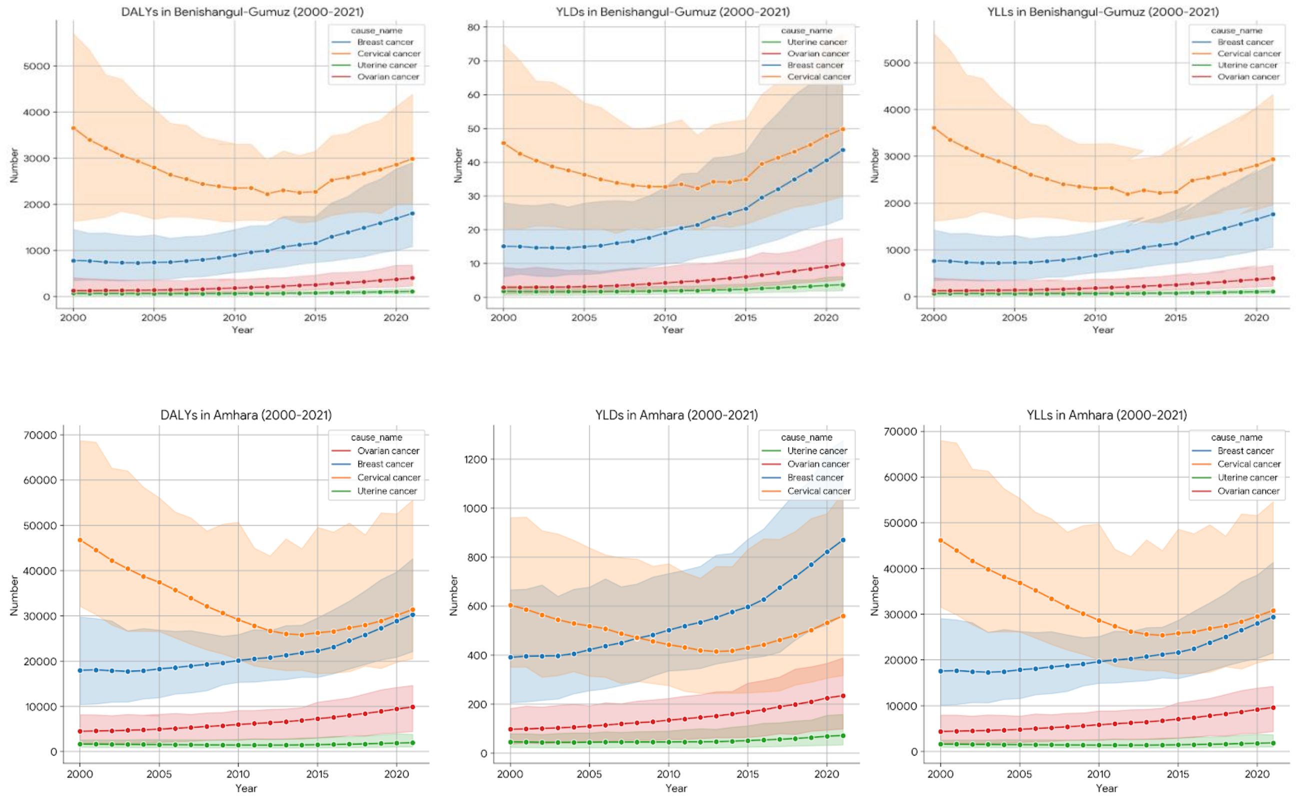

Figure 4: the Sub-national DALYs, YLD, and YLLs trend of Female specific cancer in Ethiopia (2000-2021)

Age-standardized burden of female beast, cervical, ovarian and uterine Cancers in Ethiopia: A Comparative Analysis, 2000 and 2021

The breast cancer burden, quantified in DALYs, remarkably increased in the 75+ age group (29.97% rises) and modestly in the 15-49 age group (7.23% rise) between 2000 and 2021. There was a slight decrease in DALYs in the 50-74 age groups (-0.77%). There was a constant and notable increase in YLDs among all age groups, with the highest percentage changes in the 15-49 years (47.43%) age group and second highest in 75+ years (46.68%). YLLs also presented in the same trends as DALYs, with a notable increase in the 75+ year's age group (29.48%). This reflects an increasing burden of living with breast cancer among younger and older females.

Contrary to the sharp increase in breast cancer, the cervical cancer burden experienced a steep decrease in all age groups and in all three measures. DALYs due to cervical cancer registered steady decreases, with 50-74 age groups having the highest percentage decrease at -54.48%,

followed by the 15-49 age groups at -53.90%. The same steep decreases were registered for YLDs (-43.54% in 50-74 years) and YLLs (e.g., -54.60% in 50-74 years). These results reflect a welcome change in the epidemiological trend of cervical cancer.

Uterus cancers also experienced an overall reduction in DALYs and YLLs across all age groups. The most significant reductions in DALYs and YLLs were in the 15-49 and 50-74 years age groups (approximately -27% to -28%). For YLDs, the changes were minimal, with a slight increase in the 15-49 and 75+ age groups and a slight decrease in the 50-74 age groups. The burden of ovarian cancer rose in every age group and for every measure (DALYs, YLDs, and YLLs). The 15-49 age groups had the largest percent rise in YLDs (58.25%) and DALYs (38.42%). The 75+ age group also experienced steep rises in DALYs (30.15%) and YLLs (30.05%) **(Table1).** Trends indicate an increasing public health issue of ovarian cancer.

Table 1: Age-standardized burden of female beast, cervical, ovarian and uterine Cancers in Ethiopia (2000 and 2021)

Cause	Measure	Age class	ASR (95% UI) in 2000	ASR(95% UI) in 2021	Absolute Change	Annual Percentage Change (%)
Breast cancer	DALYs	15-49 years	283.08 (189.73-411.95)	303.56 (233.13-389.26)	20.47	7.23
		50-74 years	1471.96 (1018.38-2106.47)	1460.69 (1148.49-1854.18)	-11.27	-0.77
		75+ years	1602.75 (1173.46-2119.78)	2083.16 (1697.83-2528.49)	480.41	29.97
	YLDs	15-49 years	5.50 (3.11-8.44)	8.11 (5.19-11.82)	2.61	47.43
		50-74 years	34.56 (21.62-52.62)	43.19 (28.74-60.71)	8.63	24.97
		75+ years	46.19 (31.28-64.36)	67.75 (47.39-88.88)	21.56	46.68
	YLLs	15-49 years	277.58 (186.12-404.44)	295.45 (225.79-378.38)	17.86	6.44
		50-74 years	1437.40 (996.37-2056.99)	1417.51 (1115.02-1802.42)	-19.90	-1.38
		75+ years	1556.56 (1140.69-2056.55)	2015.40 (1643.73-2449.76)	458.84	29.48
Cervical cancer	DALYs	15-49 years	645.59 (494.56-934.61)	297.61 (208.99-459.31)	-347.98	-53.90
		50-74 years	3935.62 (3015.53-5586.80)	1791.67 (1318.57-2525.78)	-2143.95	-54.48
		75+ years	1816.55 (1292.62-2671.46)	1134.13 (846.46-1528.98)	-682.42	-37.57
	YLDs	15-49 years	9.68 (6.31-14.36)	6.58 (3.97-10.60)	-3.10	-32.03
		50-74 years	44.81 (30.33-64.15)	25.30 (16.40-39.77)	-19.51	-43.54
		75+ years	23.24 (14.28-34.59)	15.76 (10.13-23.50)	-7.48	-32.20
	YLLs	15-49 years	635.91 (487.46-920.23)	291.03 (204.86-449.56)	-344.88	-54.23
		50-74 years	3890.81 (2979.37-5532.35)	1766.38 (1300.26-2493.46)	-2124.43	-54.60
		75+ years	1793.31 (1277.20-2643.59)	1118.38 (834.36-1505.45)	-674.93	-37.64

Uterine cancer	DALYs	15-49 years	10.79 (6.51-15.77)	7.78 (4.68-14.66)	-3.01	-27.86
		50-74 years	189.24 (120.53-270.51)	136.96 (85.39-233.96)	-52.27	-27.62
		75+ years	226.02 (140.04-339.96)	194.16 (121.99-300.22)	-31.86	-14.10
	YLDs	15-49 years	0.26 (0.14-0.42)	0.27 (0.14-0.57)	0.01	3.22
		50-74 years	5.41 (3.01-8.87)	5.36 (3.05-10.25)	-0.06	-1.04
		75+ years	5.35 (2.92-8.91)	5.79 (3.34-10.11)	0.44	8.17
	YLLs	15-49 years	10.53 (6.34-15.40)	7.51 (4.51-14.15)	-3.01	-28.64
		50-74 years	183.83 (117.02-261.32)	131.61 (81.82-223.84)	-52.22	-28.41
		75+ years	220.66 (135.99-331.64)	188.37 (117.92-291.14)	-32.29	-14.64
Ovarian cancer	DALYs	15-49 years	42.06 (22.90-74.52)	58.23 (28.55-84.50)	16.16	38.42
		50-74 years	424.40 (189.84-750.27)	523.08 (258.83-752.73)	98.68	23.25
		75+ years	374.11 (131.76-623.87)	486.92 (243.12-681.78)	112.81	30.15
	YLDs	15-49 years	1.11 (0.55-2.17)	1.75 (0.90-2.77)	0.64	58.25
		50-74 years	8.23 (3.55-15.79)	10.78 (5.16-16.73)	2.55	31.02
		75+ years	7.41 (2.45-14.24)	10.01 (4.76-16.29)	2.60	35.07
	YLLs	15-49 years	40.96 (22.36-72.40)	56.48 (27.64-82.02)	15.52	37.89
		50-74 years	416.17 (185.76-736.62)	512.29 (254.12-736.01)	96.13	23.10
		75+ years	366.69 (128.83-610.22)	476.90 (238.13-666.55)	110.21	30.05

Age-standardize prevalence, incidence and mortality rate of breast, cervical, uterine and ovarian cancer in Ethiopia

Prevalence rates analysis:

Breast cancer prevalence rates were consistently the highest in age group 70+, with a notable rise from 561.72 per 100,000 populations in 2000 to 798.86 in 2021. Conversely, the 50–54 years age group had the highest cervical cancer incidence, which fell spectacularly from 384.40 per 100,000 populations in 2000 to 238.15 in 2021. For age group 60–64 years, ovarian cancer prevalence increased, from 55.80 per 100,000 populations in 2000 to 81.27 in 2021. Age group 65–69 years old had the highest uterine cancer prevalence, which increased from 78.12 per 100,000 in 2000 to 96.65 in 2021**(Figure 5).**

Incidence rates analysis:

The highest incidence of breast cancer was in the 70+ age group, which increased from 103.52 per 100,000 populations in 2000 to 144.32 in 2021. In the case of cervical cancer, the highest incidence changed; in 2000, the highest incidence was in the 60−64 age group, whereas in 2021, the highest incidence age group changed to 65−69, showing a huge decrease from 162.41 per 100,000 populations in 2000 to 91.72 in 2021. The highest incidence rates of ovarian cancer

were observed in the 70+ age group, which increased from 22.74 per 100,000 populations in 2000 to 30.23 in 2021. The highest incidence of uterine cancer was also in the 70+ age group, but there was a slight decrease from 16.57 per 100,000 populations in 2000 to 16.53 in 2021**(Figure 5).**

Mortality (Deaths) rates analysis:

Breast cancer death rates were highest among the 70+ year age group, rising from 105.35 per 100,000 populations in 2000 to 135.60 in 2021. In contrast, for deaths due to cervical cancer, the highest age group changed from 65−69 years old in 2000 to 70+ years old in 2021, and the rates declined dramatically from 146.91 per 100,000 populations in 2000 to 84.51 in 2021. Death rates of ovarian cancer were also highest among the 70+ year age group, rising from 23.24 per 100,000 populations in 2000 to 31.24 in 2021. Finally, for uterine cancer, the highest death rate continued to be in the 70+ year age group, falling from 14.86 per 100,000 populations in 2000 to 12.84 in 2021 **(Figure 5).**

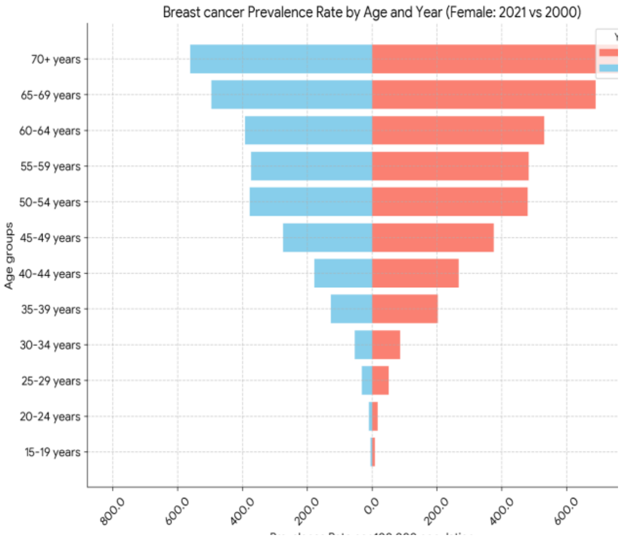
Breast cancer Prevalence Rate by Age and Year (Female: 2021 vs 2000)
Age groups
70+ years
65-69 years
60-64 years
55-59 years
50-54 years
45-49 years
40-44 years
35-39 years
30-34 years
25-29 years
20-24 years
15-19 years
800.0
600.0
400.0
200.0
0.0
200.0
400.0
600.0

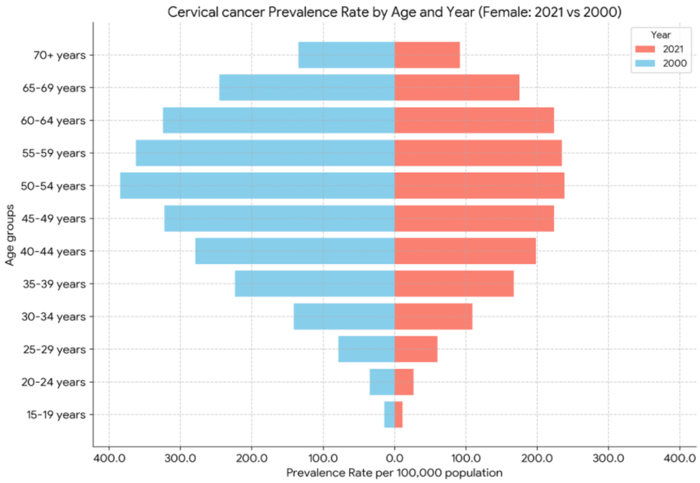
Cervical cancer Prevalence Rate by Age and Year (Female: 2021 vs 2000)
Year
2021
2000
Age groups
70+ years
65-69 years
60-64 years
55-59 years
50-54 years
45-49 years
40-44 years
35-39 years
30-34 years
25-29 years
20-24 years
15-19 years
400.0
300.0
200.0
100.0
0.0
100.0
200.0
300.0
400.0
Prevalence Rate per 100,000 population

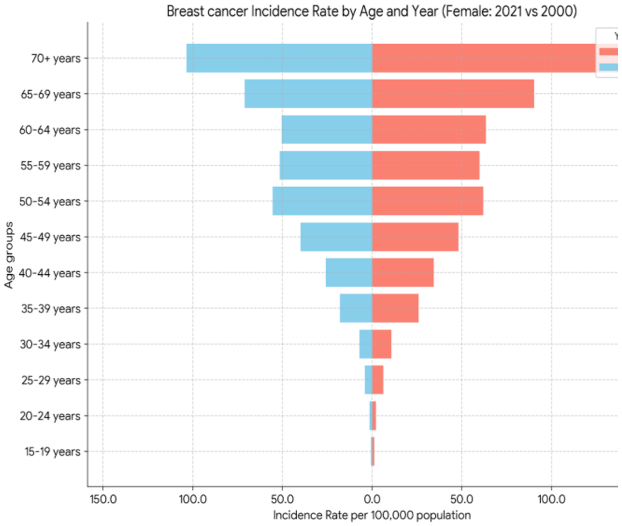
Breast cancer Incidence Rate by Age and Year (Female: 2021 vs 2000)
Age groups
70+ years
65-69 years
60-64 years
55-59 years
50-54 years
45-49 years
40-44 years
35-39 years
30-34 years
25-29 years
20-24 years
15-19 years
150.0
100.0
50.0
0.0
50.0
100.0
Incidence Rate per 100,000 population

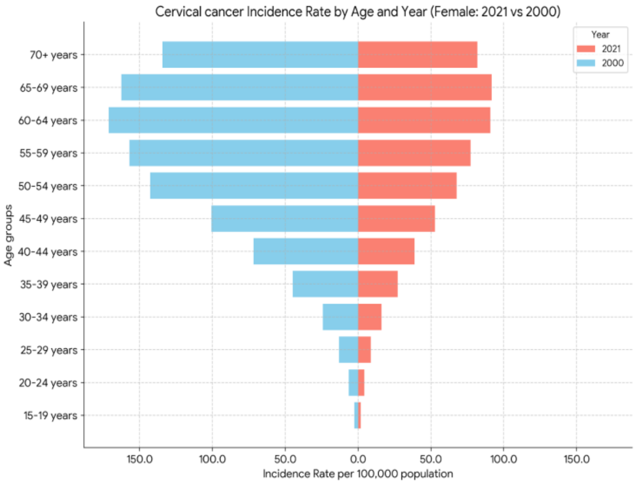
Cervical cancer Incidence Rate by Age and Year (Female: 2021 vs 2000)
Year
2021
2000
Age groups
70+ years
65-69 years
60-64 years
55-59 years
50-54 years
45-49 years
40-44 years
35-39 years
30-34 years
25-29 years
20-24 years
15-19 years
150.0
100.0
50.0
0.0
50.0
100.0
150.0
Incidence Rate per 100,000 population

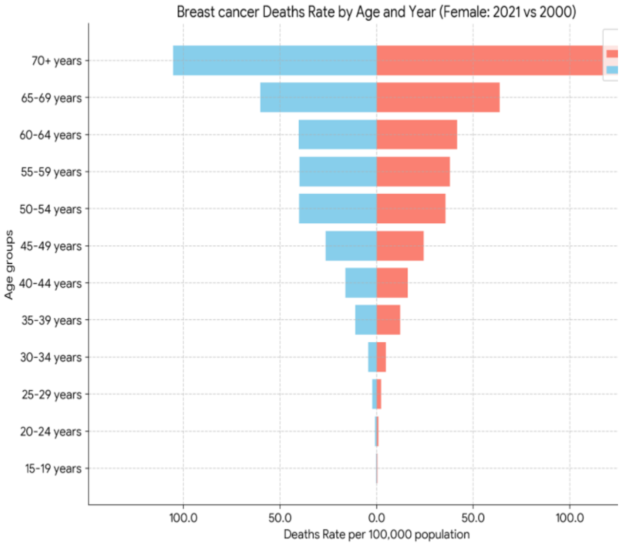
Breast cancer Deaths Rate by Age and Year (Female: 2021 vs 2000)
Age groups
70+ years
65-69 years
60-64 years
55-59 years
50-54 years
45-49 years
40-44 years
35-39 years
30-34 years
25-29 years
20-24 years
15-19 years
100.0
50.0
0.0
50.0
100.0
Deaths Rate per 100,000 population

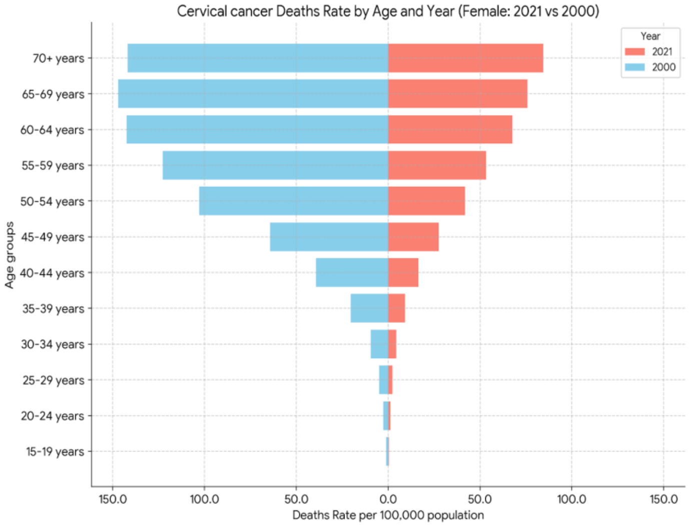
Cervical cancer Deaths Rate by Age and Year (Female: 2021 vs 2000)
Year
2021
2000
Age groups
70+ years
65-69 years
60-64 years
55-59 years
50-54 years
45-49 years
40-44 years
35-39 years
30-34 years
25-29 years
20-24 years
15-19 years
150.0
100.0
50.0
0.0
50.0
100.0
150.0
Deaths Rate per 100,000 population

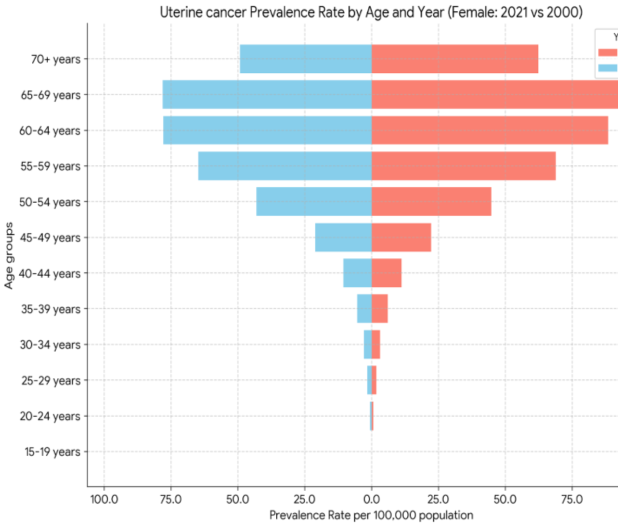
Uterine cancer Prevalence Rate by Age and Year (Female: 2021 vs 2000)
70+ years
65-69 years
60-64 years
55-59 years
50-54 years
45-49 years
40-44 years
35-39 years
30-34 years
25-29 years
20-24 years
15-19 years
Age groups
100.0
75.0
50.0
25.0
0.0
25.0
50.0
75.0
Prevalence Rate per 100,000 population

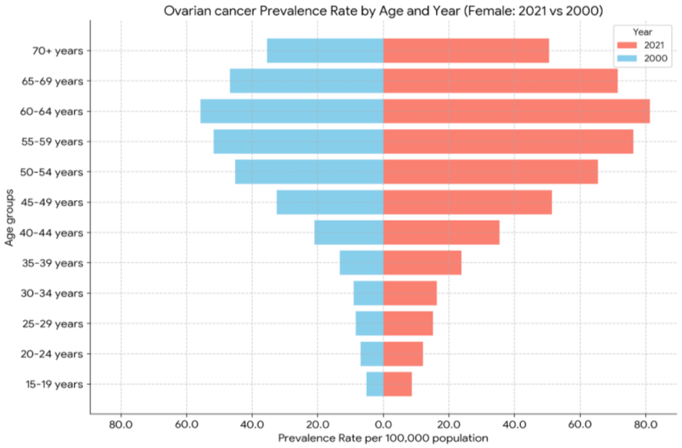
Ovarian cancer Prevalence Rate by Age and Year (Female: 2021 vs 2000)
Year
2021
2000
70+ years
65-69 years
60-64 years
55-59 years
50-54 years
45-49 years
40-44 years
35-39 years
30-34 years
25-29 years
20-24 years
15-19 years
Age groups
80.0
60.0
40.0
20.0
0.0
20.0
40.0
60.0
80.0
Prevalence Rate per 100,000 population

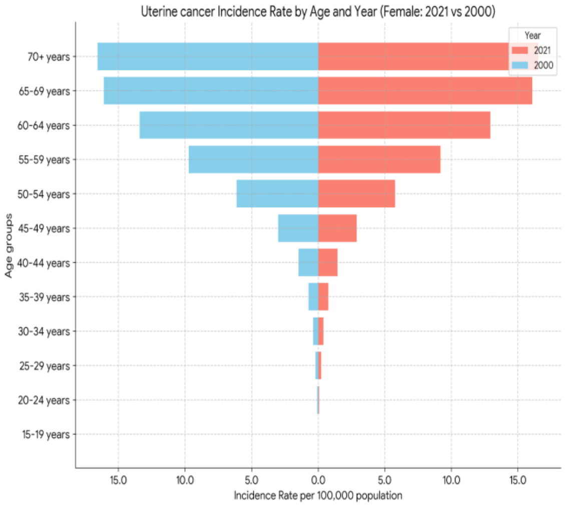
Uterine cancer Incidence Rate by Age and Year (Female: 2021 vs 2000)
Year
2021
2000
70+ years
65-69 years
60-64 years
55-59 years
50-54 years
45-49 years
40-44 years
35-39 years
30-34 years
25-29 years
20-24 years
15-19 years
Age groups
15.0
10.0
5.0
0.0
5.0
10.0
15.0
Incidence Rate per 100,000 population

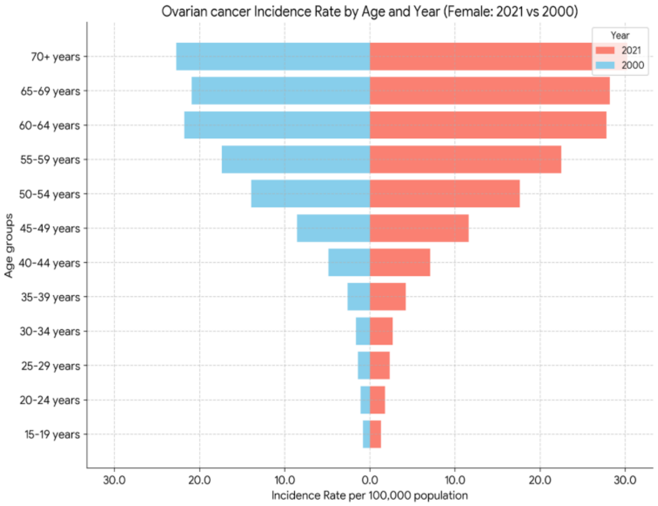
Ovarian cancer Incidence Rate by Age and Year (Female: 2021 vs 2000)
Year
2021
2000
70+ years
65-69 years
60-64 years
55-59 years
50-54 years
45-49 years
40-44 years
35-39 years
30-34 years
25-29 years
20-24 years
15-19 years
Age groups
30.0
20.0
10.0
0.0
10.0
20.0
30.0
Incidence Rate per 100,000 population

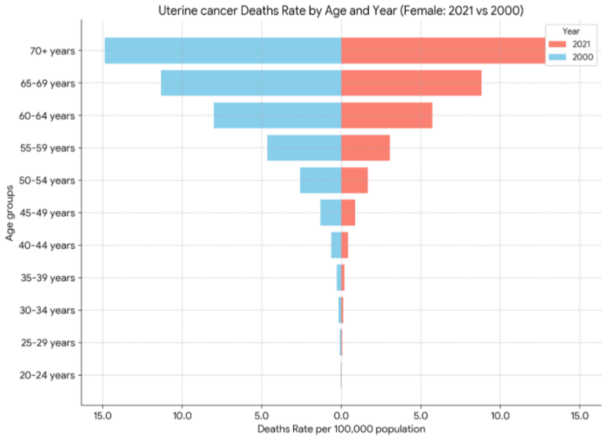
Uterine cancer Deaths Rate by Age and Year (Female: 2021 vs 2000)
Year
2021
2000
70+ years
65-69 years
60-64 years
55-59 years
50-54 years
45-49 years
40-44 years
35-39 years
30-34 years
25-29 years
20-24 years
Age groups
15.0
10.0
5.0
0.0
5.0
10.0
15.0
Deaths Rate per 100,000 population

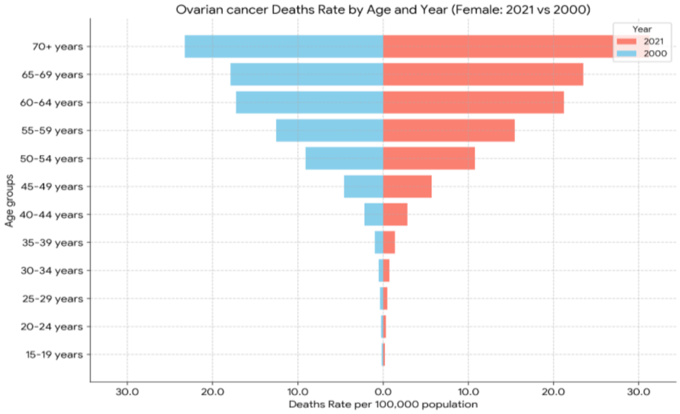
Ovarian cancer Deaths Rate by Age and Year (Female: 2021 vs 2000)
Year
2021
2000
70+ years
65-69 years
60-64 years
55-59 years
50-54 years
45-49 years
40-44 years
35-39 years
30-34 years
25-29 years
20-24 years
15-19 years
Age groups
30.0
20.0
10.0
0.0
10.0
20.0
30.0
Deaths Rate per 100,000 population

Figure 5: Age-standardize prevalence, Incidence and mortality rate of Breast, Cervical, and Uterine and Ovarian cancer in Ethiopia

The National burden prediction for female breast, cervical, uterine and ovarian cancer in Ethiopia to 2040

The ARIMA model projects a continued increasing trend in incidence rates for all analyzed female cancer types in Ethiopia from 2022 to 2040. The projected incidence rates (per 100,000 population) for each cancer type by 2040, along with their 95% uncertainty intervals (UI), which indicate the range within which the true future incidence is likely to fall and provide an important indication of the potential future burden of these diseases.

In 2021**,** the incidence of ovarian cancer was approximately 3.80 per 100,000. The incidence rate is projected to rise to approximately 5.80 (95% UI: 4.45 - 7.15) per 100,000 by 2040. This suggests a notable increase but also indicates a considerable range of possible outcomes. Although the 2021 incidence of cervical cancer was 14.59 per 100,000, its incidence is projected to increase significantly to approximately 21.10 (95% UI: 8.21 - 33.98) per 100,000. This wide interval highlights the higher uncertainty associated with the long-term forecast for this cancer type, despite the clear increasing trend. Breast cancer incidence is also projected to increase from 13.53 per 100,000 in 2021 to 20.66 (95% UI: 13.93-27.39) per 100,000 in 2040, indicating a substantial anticipated increase. For uterine cancer, the incidence in 2021 was approximately 1.24 per 100,000; however, in 2040 it is projected to rise to 1.84 (95% UI: 1.16-2.51) per 100,000, suggesting a more predictable, albeit increasing, trend compared to cervical cancer **(Figure 6).**

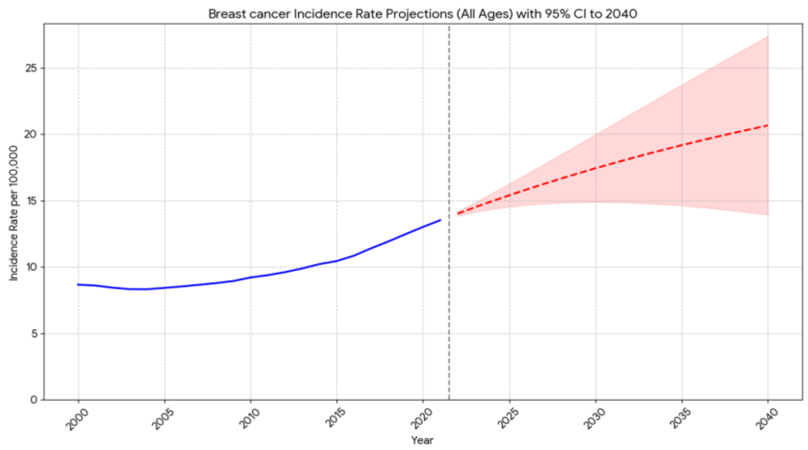
Breast cancer Incidence Rate Projections (All Ages) with 95% CI to 2040
Incidence Rate per 100,000
Year
0
5
10
15
20
25
2000
2005
2010
2015
2020
2025
2030
2035
2040

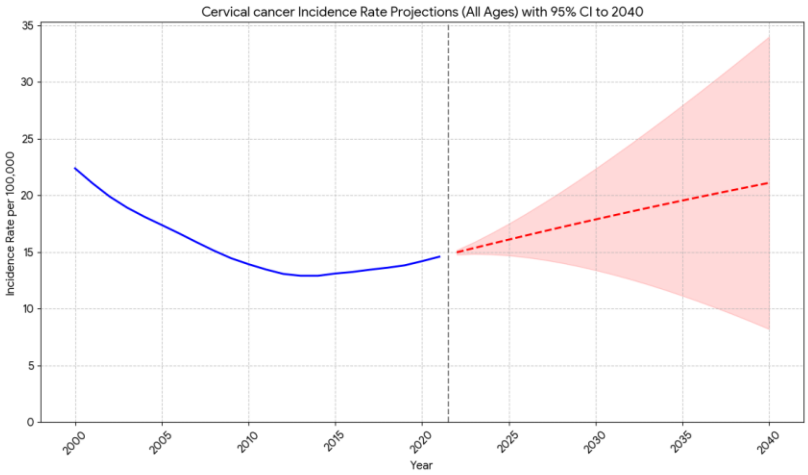
Cervical cancer Incidence Rate Projections (All Ages) with 95% CI to 2040
Incidence Rate per 100,000
Year
0
5
10
15
20
25
30
35
2000
2005
2010
2015
2020
2025
2030
2035
2040

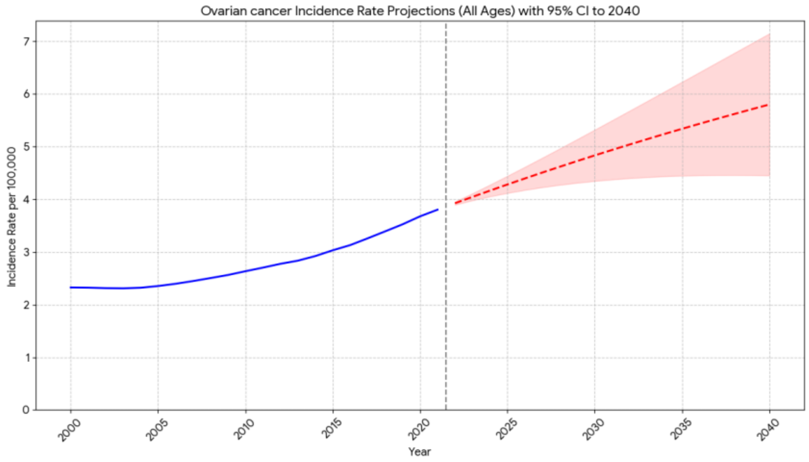
Ovarian cancer Incidence Rate Projections (All Ages) with 95% CI to 2040
Incidence Rate per 100,000
Year
0
1
2
3
4
5
6
7
2000
2005
2010
2015
2020
2025
2030
2035
2040

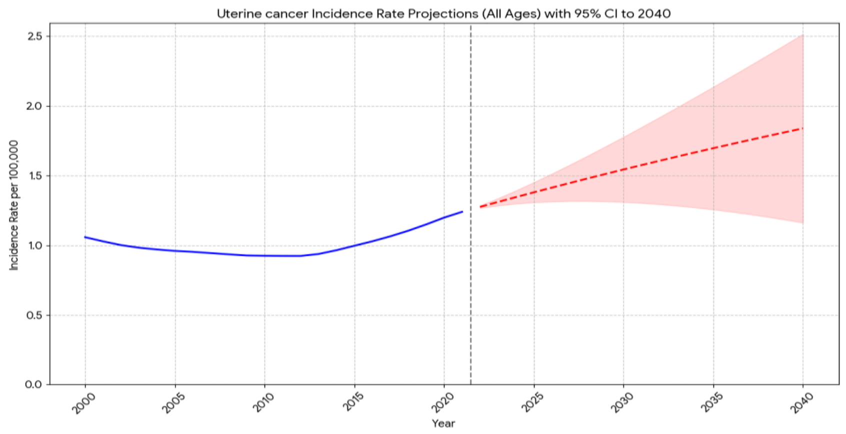

Figure 6: Projections of the Incidence Rates for female specific cancer in Ethiopia from 2022 to 2040

Percentage contribution of risk factors to all-age DALYs of cervical cancer, uterine cancer, and ovarian cancer in 2021

In 2021 a diet high in red meat was the leading risk factors for breast cancer account 11.34% (95% UI: 0.00–24.13) of cases followed by high alcohol use is associated with 2.65% (95% UI: 0.95–4.33) cases. Smoking contributes to 0.18% (95% UI: 0.12–0.25) of breast cancer cases and 1.41% (95% UI: 0.75–2.24) of cervical cancer cases. Second hand smoke exposure is linked to 0.56% (95% UI: −0.13–1.31) of breast cancer cases. High fasting plasma glucose is associated with 1.66% (95% UI: −0.46–3.92) of breast cancer cases, while high body-mass index (BMI) is linked to 0.30% (95% UI: −0.54–1.18) of breast cancer cases, 2.01% (95% UI: 0.03–4.41) of ovarian cancer cases, and 12.53% (95% UI: 9.11–16.68) of uterine cancer cases. Low physical activity accounts for 1.24% (95% UI: 0.24–2.29) of breast cancer cases. Notably, unsafe sex is responsible for 100% (95% UI: 100–100) of cervical cancer cases, highlighting its dominant role in this cancer type.

Discussion

This study critically examines the epidemiological landscape of common female breast, cervical, ovarian and uterine cancer in Ethiopia from 2000-2021, integrating insights from the national and sub-national (regional) trends in prevalence, incidence, mortality, DALYs, YLDs, and YLLs and associated risk factors. Additionally, it includes the projected incidence rate till 2040,

providing comprehensive understanding of the evolving cancer burden and its implications for public health.

At the national level, the statistics reveal a clear trend: the prevalence of cancers among women is increasingly affecting the female population in Ethiopia. Most notable is the finding that breast cancer has seen a dramatic increase, marked by an increase in both the prevalence and the rate of new cases. This finding is consistent with an international trend, where more and more women in countries like Ethiopia are being diagnosed with breast cancer, perhaps due to changes in lifestyle, diet, urbanization, and improvements in disease detection equipment (11-13). The dramatic increase in "Years Lived with Disability" (YLDs) related to breast cancer is particularly germane, as the result show that an increasing number of women are surviving with the condition; however, many struggle with the long-term effects of the disease as well(14). This finding highlights an immediate need for continued, supportive care and services focused on helping women cope with their lives after diagnosis.

The trend of cervical cancer is slightly more complex. Although the incidence and prevalence of cervical cancer are increasing, it is encouraging that there is a decline in the overall burden measured in DALYs. This result was consistent with the global studies specifically in breast cancer(14, 15). This is primarily because YLLs have decreased, indicating that those women who were dying from the disease are not losing lives to it because screening and treatment options are improving. At the same time however, the rise in YLDs serves as a reminder that despite better survival rates, numerous women still face the chronic challenges of living with cervical cancer which underscores the need for palliative care and rehabilitation to enhance these women's daily lives.

Amongst cancers affecting woman's health, ovarian cancer shows truly alarming upward trends. We are witnessing "spectacular" increases in both incidence and prevalence alongside a stunning surge in the burden it places on women's health. Consistent finding were reported in global context (16). What is striking at this stage is also noticing the increased trend for YLD which reflects a very fast escalatory nature amidst severe burden among affected populations. This rapid escalation demands urgent attention, suggesting we need better ways to detect this cancer early and more effective treatments, as it often goes unnoticed until advanced stages.

Alongside other cancers, the increase in YLDs with uterine cancer diagnosis is noticeably sharp. Similarly the global GBD result also showed the consistent increment of uterine cancer especially in aged greater than 55 years old(17, 18). This data showcases rising challenges that women face in managing a health condition—disability—after diagnosis. There is an apparent gap for enhanced recovery support and care to help them recover fully.

Sub-National Dynamics: Understanding Local Realities

Oromia and Amhara regions exhibit greater population density, along with burly female cancer prevalence which indicates notable regional disparities compared to national averages. Therefore, it can be stated that the availability of preventive and treatment services must match the demands of these dense populations. Addis Ababa also exhibits a high concentration of breast and uterine cancers. The pronounced rises in YLDs here may suggest that more healthcare and diagnostic facilities available in urban areas are uncovering cases that are being diagnosed at an early stage. Although this is positive in terms of uncovering cases, it also illustrates the hidden burden of living with the illness. The reduction in cervical cancer DALY's amongst women from Addis Ababa further implies that there is greater or faster aim urban advantage often cited for geographically targeted accessible prevention and screening initiatives. The situation in SNNP Region is particularly concerning as all measures signal a near twofold increase in ovarian cancers. Such rapid increases demand immediate and focused interventions to understand why this is happening and to put effective control measures in place. A crucial point is highlighted by these regional variations: there is no one approach that works for Ethiopia as a whole. To ensure that assistance reaches the areas where it is most needed, cancer control programs must be customized to the particular difficulties and circumstances of each local community.

Age Standardized Burden: Who is most affected?

Studying trend of cancer by age provides critical insights into which groups are most vulnerable. For breast cancer, the increasing DALYs and YLDs across various age groups, especially among younger women (15-49) and older women (75+), means that women of all ages are increasingly living with the disease and its impact. This calls for age-appropriate screening guidelines, compassionate support services, and awareness campaigns that recognize the unique life stages and challenges faced by younger women (like balancing cancer with family or career) and older women (who may have other health conditions). In a remarkable and promising shift, the impact

of cervical cancer has lessened significantly among all age groups. The notable decline in DALYs and YLLs among women aged 15 - 49 and 50 -74 indicates that public health initiatives, potentially human papilloma virus (HPV) vaccination campaigns along with screening boosts, are truly saving lives and decreasing the burden of this disease(19). This trend is inspiring as it highlights the benefits of targeted preventive measures coupled with early diagnostics.

Compared to this progress, the situation remains bleak for ovarian cancer, which is rising across all age groups. Younger working women (15-49 years) display the most pronounced increase in YLDs and DALYs associated with particular disability burdens. There is a striking need to focus on developing timely detection systems along with better treatment strategies for this aggressive form of cancer targeting younger populations(20). While uterine cancer has seen overall reductions in its burden, a slight increase in YLDs in some age groups reminds us that comprehensive post-treatment care remains essential.

According to the risk factor analysis result, a diet high in red meat, high alcohol use, smoking, second hand smoke exposure, high fasting plasma glucose, high body-mass index, low physical activity were the identified risk factors for breast cancer(21, 22). While unsafe sex and smoking were the leading risk factors for cervical cancer(23). High BMI were the primary risk factors for ovarian and uterine cancer(24, 25). These findings underscore the varying contributions of modifiable risk factors across different cancers, with some exhibiting stronger associations than others.

Projection Implications

The results of projection uniformly indicate a rising burden of female cancers in Ethiopia over the next two decades. Breast and ovarian cancer are expected to rise substantially. Uterine cancer, on the other hand, exhibits a more consistent but nonetheless raising its trend. Despite the encouraging past reductions in DALYs and YLLs, cervical cancer incidence is projected to increase significantly by 2040. The consistent upward trend across all four cancer types suggests that, if historical patterns persist, the demand for cancer diagnosis, treatment, and palliative care services will significantly increase.

This study, similar to other GBD analyses, has limitations stemming from data integrity and quality, particularly in low socioeconomic development regions including Ethiopia, which can

lead to potential underestimations, misdiagnoses, and data heterogeneity. Reliance on simulation techniques in data-scarce areas compromises accuracy, and projections based solely on historical trends may not fully capture dynamic disease changes or the evolving influence of unanalyzed or emerging risk factors. Additionally, the study's scope was limited to female breast, cervical, uterine, and ovarian cancers, excluding other female cancer types and specific tumor pathological features, and the risk factors considered were confined to those within the GBD database, potentially influencing the comprehensiveness of the findings and hindering precise prevention and control efforts.

Conclusion and recommendations

Ethiopia is facing with a dynamic and increasing burden of female cancers that is characterized by a substantial increase in breast and ovarian cancer incidence and disability in the past two decayed and projected increase in all female cancer types including cervical cancer by 2040. Even though the mortality from cervical cancer has declined, the total burden of women living with cancer-related disability underscores vital unmet needs in supportive care. It is clear that addressing this multifaceted challenge requires a comprehensive national cancer control strategy that is contextualized regionally, with a heightened emphasis on prevention and early detection and expanded capacity for diagnosis and treatment, comprehensive palliative care, and ongoing investment in policy and research to protect women's health in Ethiopia.

Declarations

Data availability

The data used in this study accessed from to the GBD 2021 official website is available at https://ghdx.healthdata.org/gbd-2021.

Consent for publication: All authors have read and approved the final version of the manuscript for publication.

Competing interests: All authors declare no competing interests related to the publication of this article.

Funding: None

Authors' Contributions: MAA: Conceptualization, data curation, formal analysis, methodology, software, visualization, writing – original draft, writing – review & editing. **TEH:** Conceptualization, formal analysis, methodology, software, writing – original draft, writing –

review & editing. **TAK:** Conceptualization, formal analysis, methodology, software, writing – original draft, writing – review & editing. **ADG:** formal analysis, methodology, writing – original draft, writing – review & editing. **BMW:** data curation, formal analysis, methodology, writing – original draft, writing – review & editing. **YAD:** formal analysis, methodology, writing – original draft, writing – review & editing. **ABZ:** data curation, formal analysis, methodology, writing – original draft, writing – review & editing.

References

1. Bray F, Laversanne M, Sung H, Ferlay J, Siegel RL, Soerjomataram I, et al. Global cancer statistics 2022: GLOBOCAN estimates of incidence and mortality worldwide for 36 cancers in 185 countries. 2024;74(3):229-63.
2. World Health Organization (WHO). Fact Sheet on Cancer:. February 3, 2025.
3. Bray F, Soerjomataram IJC. The changing global burden of cancer: transitions in human development and implications for cancer prevention and control. 2015;3(1):23-44.
4. Morhason-Bello IO, Odedina F, Rebbeck TR, Harford J, Dangou J-M, Denny L, et al. Challenges and opportunities in cancer control in Africa: a perspective from the African Organisation for Research and Training in Cancer. 2013;14(4):e142-e51.
5. Awedew AF, Berheto TM, Dheresa M, Tadesse S, Hailmariam A, Tollera G, et al. The Burden of Non-Communicable Diseases and Its Implications for Sustainable Development Goals Across Regions in Ethiopia. 2023;37(2).
6. Assefa MJTEJoHD. Profile of Cancer in Addis Ababa: From the only Population Based Cancer Registry in Ethiopia (2012-2021). 2023;37(4).
7. Gebretsadik A, Bogale N, Dulla DJBo. Descriptive epidemiology of gynaecological cancers in southern Ethiopia: retrospective cross-sectional review. 2022;12(12):e062633.
8. Endale H. The Socio-economic Impact of Cervical Cancer on Patients in Ethiopia: Evidence from Tikur Anbessa Specialized Hospital (TASH) 2021.
9. Mamo G, Worku A, Lemma S, Demas TJHEOROA. Cost of illness of breast cancer patients on chemotherapy in Addis Ababa Public Hospitals, the case of Tikur Anbessa specialized teaching hospital-cross-sectional types of study. 2017;3(4):1-5.
10. Haileselassie W, Mulugeta T, Tigeneh W, Kaba M, Labisso WLJJocp. The situation of cancer treatment in Ethiopia: challenges and opportunities. 2019;24(1):33.

11. World Cancer Research Fund. Breast cancer statistics. Retrieved from: https://www.wcrf.org/preventing-cancer/cancer-statistics/breast-cancer-statistics/.

12. Kim J, Harper A, McCormack V, Sung H, Houssami N, Morgan E, et al. Global patterns and trends in breast cancer incidence and mortality across 185 countries. 2025:1-9.

13. Ndubuisi NEJITJoHCO, Provision,, Financing. Noncommunicable diseases prevention in low-and middle-income countries: an overview of health in all policies (HiAP). 2021;58:0046958020927885.

14. Global BJJo. Global, regional, and national cancer incidence, mortality, years of life lost, years lived with disability, and disability-adjusted life-years for 32 cancer groups, 1990 to 2015: a systematic analysis for the global burden of disease study. 2017.

15. World Health Organization. Global strategy to accelerate the elimination of cervical cancer as a public health problem. Retrieved from https://www.who.int/initiatives/global-strategy-to-accelerate-the-elimination-of-cervical-cancer-as-a-public-health-problem.

16. Ren Y, Xu R, Su L. Global, Regional, and National Burden of Ovarian Cancer in Women Aged 45+ from 1990 to 2021 and Projections for 2050: A Systematic Analysis Based on the 2021 Global Burden of Disease Study. 2025.

17. Feng T, Li W, Wang Q, Yang J, Shen FJJoH, Population, Nutrition. Global, regional, and national burden of uterine cancer among women aged 50 years and older from 1990 to 2021: a systematic analysis for the global burden of disease study 2021. 2025;44(1):1-16.

18. Gao S, Wang J, Li Z, Wang T, Wang JJIJoWsH. Global Trends in Incidence and Mortality Rates of Endometrial Cancer Among Individuals Aged 55 years and Above From 1990 to 2021: An Analysis of the Global Burden of Disease. 2025:651-62.

19. Zhang M, Chen J, Cui M, Jia J, Zhao M, Zhou D, Zhu L, Luo L. Analysis of the global burden of cervical cancer in young women aged 15–44 years old. European Journal of Public Health. 2024 Aug;34(4):839-46.

20. Hong MK, Ding DC. Early diagnosis of ovarian cancer: A comprehensive review of the advances, challenges, and future directions. Diagnostics. 2025 Feb 7;15(4):406.

21. Mekonen H, Negesse A, Endalifer ML, Molla G, Aneley Z. Dietary factors associated with breast cancer among women in Ethiopia: a systematic review and meta-analysis of case–control studies. Frontiers in Nutrition. 2025 Feb 17;12:1499634.

22. World Cancer Research Fund / American Institute for Cancer Research. Continuous Update Project Expert Report 2018. Breast Cancer. Retrieved from https://www.wcrf.org/diet-activity-and-cancer/cancer-types/breast-cancer/.

23. American Cancer Society. Risk Factors for Cervical Cancer. Retrieved from https://www.cancer.org/cancer/types/cervical-cancer/causes-risks-prevention/risk-factors.html

24. American Cancer Society. Endometrial Cancer Risk Factors. Retrieved from https://www.cancer.org/cancer/types/endometrial-cancer/causes-risks-prevention/risk-factors.html.

25. American Cancer Society. Ovarian Cancer Risk Factors. Retrieved from https://www.cancer.org/cancer/types/ovarian-cancer/causes-risks-prevention/risk-factors.html.
