# Supplementary material for: The rising burden of female cancer in Ethiopia (2000–2021) and projections to 2040: Insights from the global burden of disease study
Source: PLoS One. 2025 Oct 7;20(10):e0333787. doi: 10.1371/journal.pone.0333787 (PMC12503343; doi:10.1371/journal.pone.0333787)
Supplement: Attachment — Submitted filename: 1-s2.0-S2666577825000875-main.pdf [file pone.0333787.s003.pdf]

Burden and risk factors of cervical cancer in Vietnam from 1990 to 2021 and forecasting to 2050—a systematic analysis from global burden disease

Bao Huy Le, MD; Thao-Ngan Nguyen Pham, MD; Huong-Dung Thi Nguyen, MD; Tri Cuong Phan, MD; Han My Nguyen Le; Thien Tan Tri Tai Truyen, MD; Tam Duc Lam, MD, PhD

BACKGROUND: Cervical cancer continues to be a significant global health challenge, with Vietnam facing similar concerns. Although there has been some progress in lowering its incidence and mortality rates, a comprehensive understanding of long-term trends and the factors driving these changes remains limited.

OBJECTIVE: This study aimed to analyze the burden and risk factors of cervical cancer in Vietnam over a 3-decade period, from 1990 to 2021 with projection to 2050.

STUDY DESIGN: Our study used data from the Global Burden of Disease (GBD) 2021 estimates, developed by the Institute for Health Metrics and Evaluation (IHME) at the University of Washington. This dataset includes metrics such as incidence, prevalence, mortality, and disability-adjusted life years (DALYs), all presented as age-standardized rates (ASRs) per 100,000 individuals. We analyzed trends from 1990 to 2021 and projections for 2022 to 2050 using the Joinpoint regression model. Additionally, we examined ASRs for death and DALYs attributed to cervical cancer-related risk factors.

RESULTS: In 2021, cervical cancer accounted for approximately 4369 deaths (95% UI: 3345−5713) in Vietnam, with ASRs for incidence, prevalence, and mortality at 16.50 per 100,000 (95% UI: 12.49−21.83), 88.11 per 100,000 (95% UI: 65.32−116.75), and 7.66 per 100,000 (95% UI: 5.90−9.94), respectively. From 1990 to 2021, trends in incidence, DALYs, and mortality followed 3 phases: a decrease in incidence from 1990 to 1992 (APC: −2.42%, 95% CI: −3.56 to −0.85), an increase from 1992 to 1996 (APC: 2.69%, 95% CI: 2.09−3.65), and a subsequent decrease from 1996 to 2008 (APC: -0.68%, 95% CI: −0.86 to −0.57), with no significant change from 2008 to 2021. Projections from 2022 to 2050 suggest an all-age DALYs rate of 147.55 (95% UI: 105.14−198.31) in 2050, with an expected rise until 2039 followed by a decline, reflecting an annual percent change of −0.2% (95% CI: −0.07 to −0.55). Major risk factors for cervical cancer in Vietnam include smoking and unsafe sexual practices, with associated age-standardized mortality rates of 0.31 (95% UI: 0.16−0.53) and 7.66 (95% CI: 5.89 −9.80) per year, respectively.

CONCLUSION: The study reveals that, despite some progress in reducing the incidence, mortality, and DALYs associated with cervical cancer in Vietnam over the past 3 decades, the overall burden remains substantial. This study underscores the impact of key risk factors, including smoking and unsafe sex. Future research is needed to evaluate effective interventions and healthcare policies that could reduce the burden of cervical cancer and improve women's health outcomes in Vietnam.

Key words: cervical cancer, epidemiologic study, burden, Vietnam, risk factors, forecasting

From the Pham Ngoc Thach University of Medicine, Ho Chi Minh City, Vietnam (B.H. Le); University of Medicine Phan and Pharmacy, Ho Chi Minh City, Vietnam (Pham); Faculty of Medicine, Nam Can Tho University, Can Tho, Vietnam (Nguyen, H.M.N. Le, and Truyen); Can Tho University of Medicine and Pharmacy, Can Tho, Vietnam (Lam)

The authors did not receive any funding affecting the study conduction, manuscript writing, or publication.

The authors report no conflict of interest.

Data availability: All data used in the manuscript analysis can be accessed on the IHME website. Further inquiries can be requested directly to the corresponding author.

Ethical consideration Institutional Review Board approval and patient consent were waived as this study utilized data from the GBD study, which is publicly available for non-commercial use on the IHME website, in accordance with the IHME free-of-charge non-commercial user agreement.

B.H.L. and T-N.N.P contributed equally to this work.

Corresponding author: Tam Duc Lam, MD, PhD. ldtam@ctump.edu.vn

2666-5778/$36.00
© 2025 The Authors. Published by Elsevier Inc. This is an open access article under the CC BY-NC-ND license (http://creativecommons.org/licenses/by-nc-nd/4.0/)
http://dx.doi.org/10.1016/j.xagr.2025.100526

AJOG Global Reports at a Glance

Why was this study conducted?

- Cervical cancer is a challenging burden in Vietnam.
- Comprehensive understanding of the current burden of cervical cancer, its long-term trends, and future projections, along with the associated risk factors in Vietnam is limited.

Key findings

- In 2021, cervical cancer was responsible for the deaths of approximately 4369 people in Vietnam. The age-standardized rate of incidence, prevalence, and mortality were 16.50, 88.11, and 7.66 per 100,000.
- From 1990 to 2021, we observed a decline in the overall incidence rate and mortality.
- Unsafe sex and smoking are the primary risk factor for cervical cancer.

What does this study add to what is known?

- Our research uncovered the burden of cervical cancer in Vietnam in more than 30 years with projection to 2050.
- We identified important risk factors and its trend over long period.

Introduction

Cervical cancer, a gynecologic malignancy primarily driven by high-risk subtypes of human papillomavirus (HPV), continues to be a major public health challenge worldwide.[1] While HPV screening and vaccination are highly effective in preventing cervical cancer, developing countries without national prevention programs, continue to struggle with a substantial burden of this condition,[2] leading to the global uneven distribution of cervical cancer, with around 90% of cases occurring in low- and middle-income countries.[3] According to GLOBALCAN 2018, cervical cancer is the fourth most common malignancy among women globally, with approximately 570,000 new cases, an incidence rate of 13.1 per 100,000, a mortality rate of 6.9 per 100,000, and accounting for 5.1% of the total estimated cancer burden in women.[4] In Vietnam, cervical cancer is currently the 7th most common cancer among women, with an incidence rate of 7.1 per 100,000. This condition is projected to rise and potentially become one of the top 5 most common cancers in the next decade.[5] Although there has been evidence of a decline in both incidence and mortality rates between 1999 and 2017, this progress has been hindered by the lack of a nationwide population-based cancer prevention program and a comprehensive national cancer registry.[5,6] Therefore, a comprehensive understanding of the current burden of cervical cancer, its long-term trends, and future projections, along with the associated risk factors, is crucial. This knowledge is essential for constructing a complete picture of the condition, evaluating the effectiveness of national policies and guidelines, and contributing to the existing literature, which primarily consists of studies focused on specific regions or short timeframes.[6] To address this objective, we conducted this study utilizing data from the Global Burden of Disease Study to evaluate the mortality and disability burden of cervical cancer in 2021, analyze its trends from 1990 to 2021, projection to 2050, and examine the influence of key risk factors on mortality associated with this condition.

Methods

Study design and data sources

The Global Burden Disease (GBD) study, conducted by the Institute for Health Metrics and Evaluation and the University of Washington, provides a comprehensive assessment of the burden of 371 diseases and injuries across 204 countries and territories from 1990 to 2021, and covering various age and gender groups.[7–9] The GBD 2021 project estimated the rates, numbers, and percentage changes in incidence, prevalence, cause-specific mortality, years of life lost (YLLs), years lived with disability (YLDs), and disability-adjusted life years (DALYs) across a wide range of diseases and conditions, details of these estimated indices were reported in previous papers.[7,8,10] The GBD 2021 estimates for the national burden of cervical cancer have been updated from the previous GBD 2019 version, incorporating improved data inputs, refined estimation models, and enhanced statistical analysis methods.[8,9] This study is compliant with the Guidelines for Accurate and Transparent Health Estimates Reporting (GATHER) statement.[11] To generate consistent and policy-relevant estimates of the cervical cancer burden, the GBD project utilized the Disease-Model Bayesian Meta-Regression tool (DisMod-MR 2.1). This tool, developed within the framework of the Bayesian Priors, Regularization, and Trimming (MR-BRT) model, intergrates all available morbidity and mortality data while incorporating established epidemiological relationships and spatial correlations. The methodology underlying the study design, data acquisition, and statistical estimation procedures has been comprehensively documented in previously published GBD reports.[8–10] Data on cervical cancer incidence, prevalence, mortality, and disability-adjusted life years (DALYs) in Vietnam, as well as deaths and DALYs attributable to related risk factors, were extracted using the GBD Results Tool—an interactive online platform that provides access to the comprehensive datasets generated by the GBD 2021 study (available at https://vizhub.healthdata.org/gbd-results/). For this study, we utilized datasets from 2 GBD estimates: cause of death (CoD) and risk factors, covering 23 age groups (from birth to 95 years and older) by sex, location, and year.[7–9] Our analysis focused specifically on the burden and risk factors for cervical cancer in Vietnam from 1990 to 2021, across 17 age groups, starting from 15 years and progressing in 5-year increments up to 95 years and older. Data from Southeast Asia (SEA) and

global metrics were included for comparative analysis.

Cervical cancer and metrics definition

According to the GBD study, cervical cancer data was primarily sourced from vital registration systems, verbal autopsy records, and cancer registries, with case determination based on specific ICD codes. The codes used included ICD-10 (C53-C53.9, D06-D06.9, D26.0, 180-180.9) and ICD-9 (219.0, 233.1, 622.1-622.2, 622.7). The input data underwent processing through the Mortality-to-Incidence Ratios (MIR) model and, subsequently, the CODEm model to produce estimated data. The detailed methodology was discussed elsewhere.[12,13]

The mortality and disability burden of cervical cancer in Vietnam was projected using mixed-effect regression modeling. This approach incorporated the Socio-Demographic Index, temporal factors, cause-specific covariates, and risk factors, following the methodology established by Foreman et al.,[14] Detailed methods have been described in other publications.[15]

Risk factors assessment

The GBD 2021 risk factor estimates provide data on 88 risk factors associated with 155 outcomes, enabling detailed analysis of risk-outcome pairs. These estimates were derived from primary data sources, including randomized controlled trials, prospective observational studies, pooled cohort studies, and case-control studies, which were used to establish relative risks for specific risk-outcome relationships. The systematic review methods employed in this process are described in detail in a previous publication.[9]

The GBD 2021 study organizes all risk factors into a 4-level hierarchical structure. Level 1 represents the broadest categories, including environmental/occupational, behavioral, and metabolic risk factors, while Level 4 provides the most detailed, grassroots classifications. In this study, we examine the influence of various risk factors on deaths and DALYs related to cervical cancer, beginning with Level 1 categories, and progressing through Levels 2, 3, and 4.[9] Consequently, 2 major risk factors—smoking and unsafe sex—were identified as significantly contributing to cervical cancer-related deaths and DALYs. These factors were included in our analysis and assessed using methods consistent with those described in previous studies.[16] In the GBD study, unsafe sex is defined as the risk of disease resulting from sexually transmitted infections, while data on smoking exposure were obtained from individual-level microdata and population-based survey reports.[9,16] The burden of cervical cancer attributable to these risk factors was estimated by calculating total DALYs and applying the corresponding population attributable fraction (PAF). The detailed modeling strategy and methodology for PAF calculation have been described elsewhere.[9]

Statistical analysis

In our study on the burden of cervical cancer in Vietnam in 2021, we reported the incidence, prevalence, mortality, YLDs, YLLs, and DALYs as age-standardized rates (ASRs), calculated based on the age structure of a standard population and expressed per 100,000 people, along with 95% uncertainty intervals (UI). The formula for calculating the ASR is: $ASR = \frac{\sum_{i=1}^{N} a_i w_i}{\sum_{i=1}^{N} w_i}$ where the age-specific rate in the i-th age group is denoted as a_i, and w_i represents the number of individuals (or the weight) in the same age group within the GBD 2021 standard population. The total number of age groups is denoted as N. The 95% UIs were defined as the 2.5th and 97.5th percentiles of the ordered 1000 draws.[17] To analyze the trend in ASR cervical cancer burden from 1990 to 2021, the Joinpoint Regression Model was used. A log transformation was applied, and the optimal number of joinpoints was identified using a permutation test with 4499 permutations. For each segment (joinpoint), we calculated the annual percent change (APC) with 95% confidence intervals (CI). A significant increase or decrease in prevalence, mortality, and DALYs rates was identified if $P < .05$; otherwise, the rates were considered stable. Over the entire period, we computed the weighted average of the annual percent changes (AAPC) along with their 95% CI. The burden of cervical cancer was forecasted for the period from 2022 to 2050 and presented as the all-age DALYs rate. For the analysis of risk factors, we examined ASRs of death and DALYs related to cervical cancer attributed to Level 1 and level 4 risk factors in similar approach. This analysis was conducted using the Joinpoint Regression Program (version 5.2.0, June 2024; Statistical Methodology and Applications Branch, National Cancer Institute).[18]

Results

Disease burden of cervical cancer in Vietnam in 2021

In 2021, cervical cancer was responsible for the deaths of approximately 4369 people (95% UI: 3345−5713) in Vietnam. The ASR of incidence, prevalence, and mortality were 16.50 per 100,000 (95% UI: 12.49−21.83), 88.11 per 100,000 (95% UI: 65.32−116.75), and 7.66 per 100,000 (95% UI: 5.90−9.94), respectively. These parameters were comparable to those observed in SEA and globally (Table 1). Additionally, cervical cancer resulted in a loss of 225.49 years per 100,000 populations (95% UI: 169.84−299.04) due to premature death, combined with 7.24 years per 100,000 population living with disability (95% UI: 4.59−10.97), leading to a total DALYs of 232.72 years per 100,000 (95% UI: 175.02−307.00). In Vietnam, the incidence, prevalence, and DALYs rate of cervical cancer began increasing in women over 25 years of age, peaking between 55 and 79, and then declined with age (Figure 1 A−C). Mortality rates generally increased with age, showing only a slight decline after age 85 (Figure 1D).

Trends of disease burden of cervical cancer in Vietnam from 1990 to 2021

From 1990 to 2021, the incidence rate of cervical cancer in Vietnam can be divided into 3 distinct periods. The first period, from 1990 to 1992, saw a decrease in the incidence rate, with an

TABLE 1

Burden of cervical cancer in Vietnam, Southeast Asia, and globally: age-standardized and number-standardized rates in 1990 and 2021

	1990								2021							
	Incidence		Prevalence		Death		DALYs		Incidence		Prevalence		Death		DALYs	
	Number of case (95% UI)	ASR (95% UI)	Number of case (95% UI)	ASR (95% UI)	Number of death (95% UI)	ASR (95% UI)	Number of DALYs (95% UI)	ASR (95% UI)	Number of case (95% UI)	ASR (95% UI)	Number of case (95% UI)	ASR (95% UI)	Number of death (95% UI)	ASR (95% UI)	Number of DALYs (95% UI)	ASR (95% UI)
Vietnam	4167.11 (3173.09 – 5426.10)	16.73 (12.75 – 21.96)	19113.25 (14193.86 -25958.32)	73.42 (54.74 – 100.50)	2294.23 (1774.50 – 2993.43)	9.63 (7.43 – 12.55)	78920.02 (60069.63 - 104251.38)	318.88 (243.11 – 421.51)	9655.20 (7258.60 – 12830.44)	16.50 (12.49 – 21.83)	52419.54 (38754.24 – 69853.14)	88.11 (65.32 – 116.75)	4369.00 (3345.03 – 5713.47)	7.66 (5.90 – 9.94)	136620.76 (102668.25-181500.95)	232.72 (175.02 – 307.00)
SEA	30364.63 (26327.24 – 34797.64)	18.06 (15.61 – 20.64)	136406.53 (117703.55 -156375.15)	74.22 (64.01 – 84.49)	16463.61 (14232.48 – 18813.30)	10.70 (9.27 – 12.18)	613110.37 (526601.48-699789.93)	362.63 (313.26 – 413.29)	58017.08 (49190.88 - 67747.07)	15.17 (12.91 – 17.65)	294147.24 (248120.21-344082.86)	75.11 (63.34 – 87.74)	27513.07 (23.668.86 - 31860.21)	7.45 (6.43 – 8.59)	931500.99 (800256.95-1086328.05)	241.92 (207.75 – 281.44)
Global	409548.49 (383207.24-438505.58)	18.11 (16.94 – 19.40)	1826378.57 (1726563.53-1929080.47)	78.15 (73.89 – 82.51)	211483.79 (195.724.27-229841.01)	9.68 (8.97 – 10.51)	7416286.99 (6841378.34 -8071399.68)	330.11 (304.67 – 359.10)	667426.40 (613030.09-726422.07)	15.32 (14.08 – 16.68)	3384543.86 (3108398.92-3697258.25)	79.30 (72.81 – 86.58)	296667.24 (272058.6 - 321905.72)	6.62 (6.07 – 7.18)	9911653.09 (9053316.79-10798305.83)	226.28 (206.51 – 246.86)

ASR, Age-standardized rate per 100,000 population; SEA, South East Asia; DALYs, disability-adjusted life years; 95% UI: 95% uncertainty interval.

Le. Burden and risk factors of cervical cancer in Vietnam from 1990 to 2021 and forecasting to 2050—a systematic analysis from global burden disease. AJOG Glob Rep 2025.

APC of −2.42% per year (95% CI: −3.56 to −0.85). The second period, from 1992 to 1996, experienced an increase in the incidence rate, with an APC of 2.69% per year (95% CI: 2.09 −3.65). The third period, from 1996 to 2008, observed a decrease, with an APC of −0.68% per year (95% CI: −0.86 to −0.57). In the most recent period, from 2008 to 2021, no statistically significant change in the incidence rate was observed (Figure 2 A). Similarly, the prevalence rate showed comparable trends from 1990 to 2007, followed by a significant increase from 2007 to 2021, with an APC of 1.04% (95% CI: 0.95 −1.15) (Figure 2B). Lastly, the trends in death rates and DALYs followed similar 3-phase changes (Figure 2C and D). The first phase, from 1990 to 1992, showed a decrease in death and DALY rates, with APCs of -3.04% (95% CI: −4.56 to −1.28) and -3.34% (95% CI: −4.62 to −1.71), respectively. The second phase, from 1992 to 1996, saw an increase in death and DALYs rates, with APCs of 2.30% (95% CI: 1.63−3.51) and 1.52% (95% CI: 1.89−2.89), respectively. The final phase, from 1996 to 2021, observed a significant decrease in death and DALYs rates, with APCs of -1.00% (95% CI: -1.05 to -0.95) and −1.51% and −0.94%, respectively. Cervical cancer in Vietnam is projected to have an all-age DALYs rate of 147.55 (95% UI: 105.14−198.31) by 2050. The trend is expected to rise from 2000 to 2039, followed by a decline toward 2050, with an annual percent change (APC) of −0.2 (95% CI: −0.07 to −0.55) (Figure 3).

Risk factors of cervical cancer in Vietnam and its changes

The most significant risk factors for cervical cancer in Vietnam were identified as smoking and unsafe sex, with age-standardized mortality rates associated with these risks being 0.31 (95% UI: 0.16−0.53) and 7.66 (95% CI: 5.89 −9.80) per year, respectively. The impact of smoking and unsafe sex on cervical cancer mortality and disability in Vietnam in 2021 was comparable to that in the SEA region and globally (Table 2). From 1990 to 2021, both risk

FIGURE 1

Burden of cervical cancer in Vietnam in 2021 across different age groups. Incidence, prevalence, and disability-adjusted life years (DALYs) (1A—C) showed a similar pattern, with rates increasing in middle age, peaking between 55 and 65 years, and then declining in the older population. In contrast, the death rate (1D) exhibited a consistent increase with advancing age.

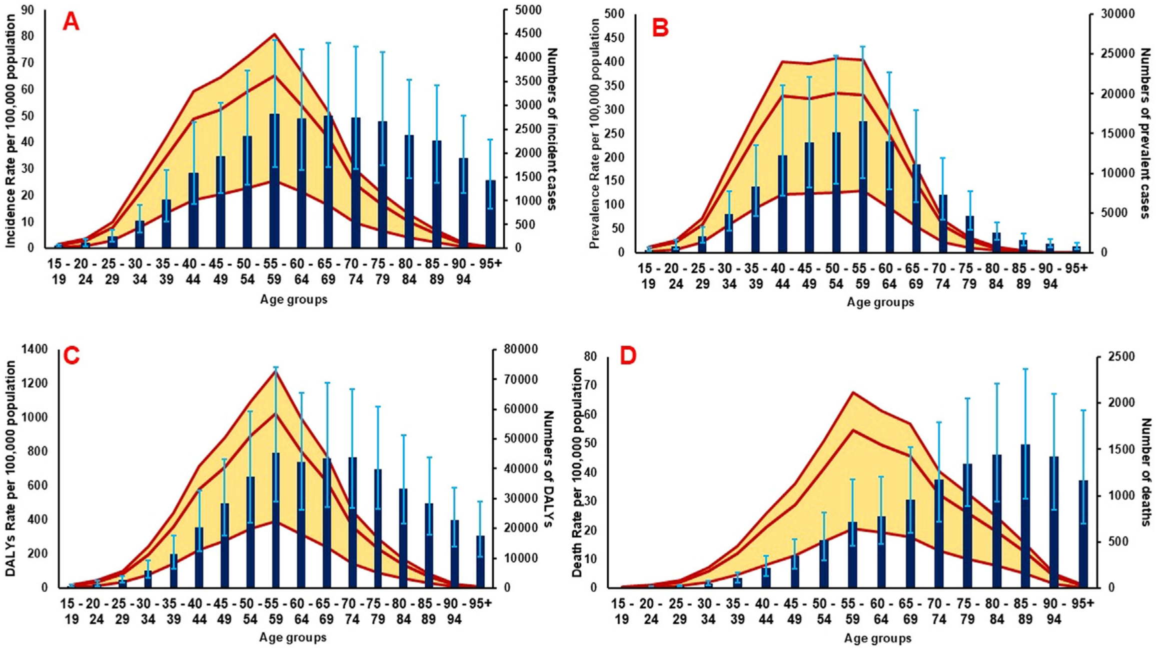

Le. Burden and risk factors of cervical cancer in Vietnam from 1990 to 2021 and forecasting to 2050—a systematic analysis from global burden disease. AJOG Glob Rep 2025.

FIGURE 2

Trends of burden of cervical cancer in Vietnam from 1990 to 2021. The age-standardized incidence, disability-adjusted life years DALYs, and death rates (2A, 2C, 2D) exhibited a decreasing trend from 1990 to 2021, whereas prevalence (2B) showed an increasing trend over the same period.

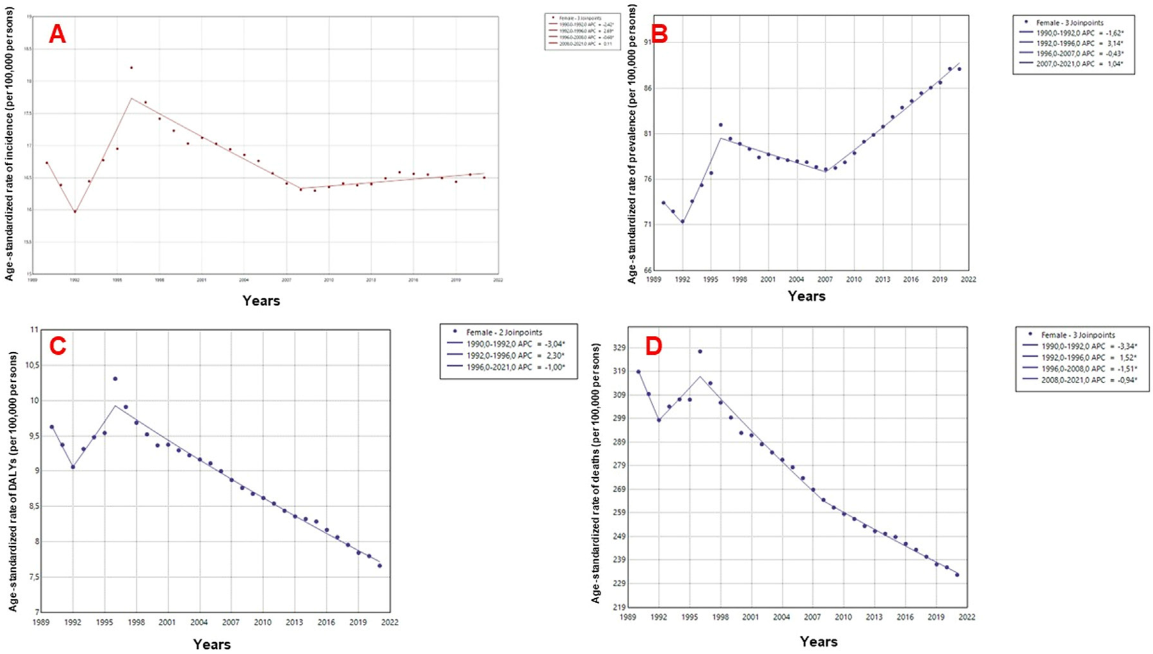

Le. Burden and risk factors of cervical cancer in Vietnam from 1990 to 2021 and forecasting to 2050—a systematic analysis from global burden disease. AJOG Glob Rep 2025.

FIGURE 3

Projection of trend of cervical cancer in Vietnam from 2022 to 2050. The all-age disability-adjusted life years trend increased from 2000 to 2021, is projected to continue rising steadily until 2039, and then is expected to decline by 2050.

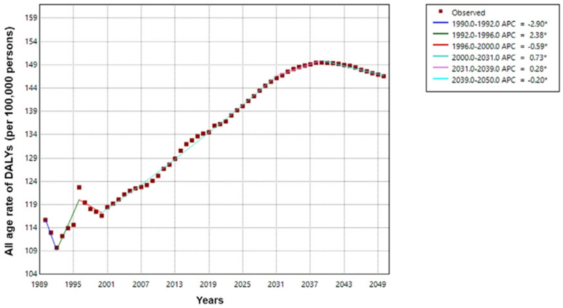

Le. Burden and risk factors of cervical cancer in Vietnam from 1990 to 2021 and forecasting to 2050—a systematic analysis from global burden disease. AJOG Glob Rep 2025.

factors showed a decreasing trend, with an APPC of −0.71 (95% CI: −0.80 to −0.66) for smoking and −2.37 (95% CI: −2.43 to −2.28) for unsafe sex.

Discussion

Based on data from GBD 2021, our study revealed that the current disease burden of cervical cancer in Vietnam is comparable to the SEA region and the global average. This condition affects nearly the entire adult population, starting from age 25 and peaking with age. From 1990 to 2021, we observed a decline in the overall incidence rate and mortality. However, we observed an increase in all-age DALYs from 1990 to 2021, with a continued rise projected through 2050. Regarding risk factors for cervical cancer in Vietnam, smoking and unsafe sex continue to play a significant role in contributing to the disease burden, necessitating more aggressive interventions and targeted healthcare policies.

The high disease burden of cervical cancer in Vietnam might be explained by many reasons. Firstly, according to a national survey and recent studies, only 25%−40% of Vietnamese women aged 30-35 have ever been screened for cervical cancer.[19,20] The main reasons for not undergoing screening include a lack of awareness about the importance of screening, limited knowledge about the screening test, misconceptions that the procedure is painful, feelings of embarrassment, and inconvenience due to the absence of nearby screening centers.[20] Secondly, nationwide understanding of the importance of HPV vaccination is limited, and access to vaccination services remains restricted. The Vietnam Sustainable Development Goals on Children and Women (SDGCW) 2020−2021 report indicated that the HPV vaccination rate stands at just 12%, with a disparity between urban and rural areas (15.6% in urban areas versus 9.2% in rural areas).[21] Unlike other Southeast Asian nations, Vietnam had not yet implemented a national HPV vaccination program. The absence of government subsidies and relative high HPV vaccine costs (25%−40% of monthly income) in Vietnam have severely limited access, resulting in low vaccination rates. This disparity is especially prominent among the poorest women, with a vaccination rate of only 2.1%, compared to 19.8% among the wealthiest women.[21] Thirdly, socioeconomic factors particularly low education levels may contribute to a greater likelihood of engaging in risky sexual behaviors and delays in accessing medical screening services, thereby increasing susceptibility to cervical cancer by 41% and 20%.[22] Finally, the Vietnam Ministry of Health recommends that cancer surgeries be performed at provincial or national hospitals. However, only 37 of the 63 provincial hospitals nationwide are equipped to provide cancer care services. The number of healthcare staff with adequate knowledge and skills for cancer treatment is insufficient, resulting in the current healthcare system being able to meet only 30% to 40% of the demand for cancer services in Vietnam due to limited resources.

The trends of cervical cancer in Vietnam had undergone significant shifts over time. The rise in the cervical cancer burden before 1996 is notably linked to

TABLE 2

Age-standardized risk factor related death and DALYs rates of cervical cancer in Vietnam, SEA, and Global in 2021

	Vietnam		SEA		Global	
	Death (95% UI)	DALYs (95% UI)	Death (95% UI)	DALYs (95% UI)	Death (95% UI)	DALYs (95% UI)
Smoking	0.31 (0.16−0.53)	8.66 (4.52−14.01)	0.45 (0.27−0.70)	13.16 (7.85−20.41)	0.5 (0.3−0.74)	16.24 (9.81−23.75)
Unsafe sex	7.66 (5.89−9.80)	232.74 (175.66−303.30)	7.45 (6.42−8.57)	241.88 (207.14−280.87)	6.62 (6.06−7.17)	226.23 (206.41−246.58)

SEA, Southeast Asia; DALYs, disability-adjusted life years; 95% UI, 95% uncertainty interval.

Le. Burden and risk factors of cervical cancer in Vietnam from 1990 to 2021 and forecasting to 2050—a systematic analysis from global burden disease. AJOG Glob Rep 2025.

the wartime conditions in Vietnam. During the Second Indochina War, from 1955 to 1975, access to cervical cancer screening and vaccination services was extremely limited, leading to a subsequent rise in incidence and prevalence 15 to 20 years later, particularly between 1990 and 1995. Furthermore, prior studies have revealed a strong association between military service and invasive cervical cancer, with women whose husbands served in combat roles in South Vietnam facing a cervical cancer risk 2.6 to 3.9 times higher than those whose husbands were civilians.[23] This phenomenon may be partially explained by previous studies, which have shown that women experiencing higher levels of chronic stress face a significantly increased risk of persistent HPV infection. This is attributed to the suppression of cell-mediated immunity —an essential component in combating HPV—caused by chronic psychological stress.[24,25] Since 1996, cervical cancer rates and related mortality in Vietnam have declined due to improved screening and management, including the introduction of Pap cytology through the Vietnam-American Prevention Project.[26] In 2002, Vietnam introduced the National Cancer Control Program with the goal of improving early cancer detection and began implementing it in 2008. Although a nationwide cervical cancer screening program has not yet been implemented, the National Cancer Control Program has supported several pilot screening projects in broader regions, expanding accessibility and reaching a larger population. These efforts, combined with the establishment of additional cancer registries have likely contributed to a slight increase in reported incidence rates due to improved detection.

Our study showed that smoking and unprotected sexual intercourse were reported to be 2 major risk factors for cervical cancer, which is in line with previous findings.[27] HPV is the most common sexually transmitted infection, typically cleared by the immune system, and both men and women have a 50% lifetime risk of contracting it.[28] In Vietnam, due to cultural specificity, sexuality education was considered as a sensitive topic and discussions about safe sexual practices were often limited and not sufficiently addressed in sexuality education both at school and at home. This resulted in a significant number of individuals engaging in unprotected sexual intercourse, raising the likelihood of contracting sexually transmitted HPV infections. Meanwhile, smoking was a confirmed HPV cofactor for the development of cervical precancer and cancer because not only smoking inhibited the immune response to HPV but also smoking carcinogens exposed to carcinogenic HPV-infected cells and caused DNA damage.[29,30] In Vietnam, the majority of smokers were men, however, smoking caused significant damage to women's health due to passive smoking. A meta-analysis by Tran Quang Duc et al.,[31] which examined studies on the prevalence of secondhand smoke exposure in Vietnam from 2010 to 2019, found that the random-effects pooled prevalence of secondhand smoking was around 55% and this prevalence increased over the course of the study period. A recent study conducted in May 2024 found that the prevalence of passive smoking exposure at home in Vietnam remained relatively high among the adult population, particularly among women.[32]

Strength and limitation

There are limitations to consider when interpreting our study. Data quality from Vietnam may vary over the years due to limited public health infrastructure, and unsafe sex rates might be overestimated because of the GBD methodology. Additionally, the analysis spans from 1990 to 2021, a period of fluctuating resource availability and data reliability, which could impact consistency and accuracy, particularly regarding misdiagnosis risks.

Despite these limitations, our study has several strengths. To our knowledge, this is the first investigation in Vietnam on the disease burden of cervical cancer, incorporating timeline analysis from 1990 to 2021 with projections to 2050. The study's longitudinal design provides valuable insights into trends in cervical cancer burden and associated risk factors. Moreover, the high-quality data from the GBD study, a trusted public health resource, enhances the validity of our findings.

Conclusion

The study reveals that, despite some progress in reducing cervical cancer incidence, mortality, and DALYs in Vietnam over the past 3 decades, the overall burden remains high. Future studies and policies are warranted to overcome barriers including financial constraints, limited access of HPV vaccination, and low awareness, while also addressing risk factors like unsafe sex and smoking. ■

CRediT authorship contribution statement

Bao Huy Le: Writing – original draft, Methodology, Conceptualization. **Thao-Ngan Nguyen Pham:** Writing – original draft, Methodology, Conceptualization. **Huong-Dung Thi Nguyen:** Writing – original draft. **Tri Cuong Phan:** Visualization, Software, Methodology, Formal analysis, Data curation. **Han My Nguyen Le:** Software, Formal analysis, Data curation. **Thien Tan Tri Tai Truyen:** Writing – review & editing, Validation, Supervision, Project administration, Methodology, Formal analysis, Conceptualization. **Tam Duc Lam:** Writing – review & editing, Supervision, Project administration, Methodology. ■

Acknowledgments

We express our deepest thanks and appreciation to the GBD2021 team at the IHME at the University of Washington for making the database available.

REFERENCES

1. Crosbie EJ, Einstein MH, Franceschi S, Kitchener HC. Human papillomavirus and cervical cancer. Lancet 2013;382(9895):889–99.
2. Sankaranarayanan R, Budukh AM, Rajkumar R. Effective screening programmes for cervical cancer in low- and middle-income developing countries. Bull World Health Organ 2001;79(10):954–62.
3. Cohen PA, Jhingran A, Oaknin A, Denny L. Cervical cancer. Lancet 2019;393(10167):169–82.

4. Bray F, Ferlay J, Soerjomataram I, Siegel RL, Torre LA, Jemal A. Global cancer statistics 2018: GLOBOCAN estimates of incidence and mortality worldwide for 36 cancers in 185 countries. CA Cancer J Clin 2018;68(6): 394–424.
5. Nguyen SM, Deppen S, Nguyen GH, Pham DX, Bui TD, Tran TV. Projecting cancer incidence for 2025 in the 2 largest populated cities in Vietnam. Cancer Control 2019;26 (1):1073274819865274.
6. Thi Nguyen DN, Simms K, Vu Nguyen HQ, et al. The burden of cervical cancer in Vietnam: Synthesis of the evidence. Cancer Epidemiol 2019;59:83–103.
7. Ferrari AJ, Santomauro DF, Aali A, et al. Global incidence, prevalence, years lived with disability (YLDs), disability-adjusted life-years (DALYs), and healthy life expectancy (HALE) for 371 diseases and injuries in 204 countries and territories and 811 subnational locations, 1990−2021: a systematic analysis for the Global Burden of Disease Study 2021. The Lancet 2024;403(10440):2133–61.
8. Naghavi M, Ong KL, Aali A, et al. Global burden of 288 causes of death and life expectancy decomposition in 204 countries and territories and 811 subnational locations, 1990−2021: a systematic analysis for the Global Burden of Disease Study 2021. The Lancet 2024;403 (10440):2100–32.
9. GBD 2021 Risk Factors Collaborators. Global burden and strength of evidence for 88 risk factors in 204 countries and 811 subnational locations, 1990-2021: a systematic analysis for the Global Burden of Disease Study 2021. Lancet 2024;403(10440):2162–203.
10. GBD 2021 Suicide Collaborators. Global, regional, and national burden of suicide, 1990-2021: a systematic analysis for the Global Burden of Disease Study 2021. Lancet Public Health 2025;10(3):e189–202.
11. Stevens GA, Alkema L, Black RE, Boerma JT, Collins GS, Ezzati M, et al. Guidelines for accurate and transparent health estimates reporting: the GATHER statement. Lancet 2016;388(10062):e19–23.
12. GBD 2021 Diseases and Injuries Collaborators. Global incidence, prevalence, years lived with disability (YLDs), disability-adjusted life-years (DALYs), and healthy life expectancy (HALE) for 371 diseases and injuries in 204 countries and territories and 811 subnational locations, 1990-2021: a systematic analysis for the Global Burden of Disease Study 2021. Lancet 2024;403(10440):2133–61.
13. GBD 2021 Causes of Death Collaborators. Global burden of 288 causes of death and life expectancy decomposition in 204 countries and territories and 811 subnational locations, 1990-2021: a systematic analysis for the Global Burden of Disease Study 2021. Lancet 2024;403(10440):2100–32.
14. Foreman KJ, Marquez N, Dolgert A, et al. Forecasting life expectancy, years of life lost, and all-cause and cause-specific mortality for 250 causes of death: reference and alternative scenarios for 2016-40 for 195 countries and territories. Lancet 2018;392(10159):2052–90.
15. Vollset SE, Ababneh HS, Abate YH, et al. Burden of disease scenarios for 204 countries and territories, 2022−2050: a forecasting analysis for the Global Burden of Disease Study 2021. The Lancet 2024;403(10440):2204–56.
16. Guan S-Y, Zheng J-X, Feng X-Y, et al. Global burden due to modifiable risk factors for autoimmune diseases, 1990−2021: Temporal trends and socio-demographic inequalities. Autoimmun Rev 2024;23(12):103674.
17. Zhang S-X, Miao F-Y, Yang J, et al. Global, regional, and national burden of HIV-negative tuberculosis, 1990−2021: findings from the Global Burden of Disease Study 2021. Infectious Diseases of Poverty 2024;13(1):60.
18. Joinpoint program: Surveillance Research Program, National Cancer Institute (2025). Joinpoint Regression Software, Version 5.4.0 - April 2025. https://surveillance.cancer.gov/joinpoint
19. Pham T, Bui L, Kim G, Hoang D, Tran T, Hoang M. Cancers in Vietnam-burden and control efforts: a narrative scoping review. Cancer Cntrl. 2019;26(1):1073274819863802.
20. Phung MT, An PL, Vinh NN, et al. A comparative study on behavior, awareness and belief about cervical cancer among rural and urban women in Vietnam. PLOS Glob Public Health 2023;3(6):e0001817.
21. UNICEF GSOa. Survey measuring Viet Nam Sustainable Development Goal indicators on Children and Women 2020-2021, Survey Findings Report. 2021.
22. Benard VB, Johnson CJ, Thompson TD, et al. Examining the association between socioeconomic status and potential human papillomavirus-associated cancers. Cancer 2008;113 (S10):2910–8.
23. Huynh ML, Raab SS, Suba EJ. Association between war and cervical cancer among Vietnamese women. Int J Cancer 2004;110 (5):775–7.
24. Coker AL, Bond S, Madeleine MM, Luchok K, Pirisi L. Psychosocial stress and cervical neoplasia risk. Biopsychos Sci Med 2003;65 (4):644–51.
25. Fang CY, Miller SM, Bovbjerg DH, et al. Perceived stress is associated with impaired T-cell response to HPV16 in women with cervical dysplasia. Ann Behav Med 2008;35 (1):87–96.
26. Suba EJ, Raab SS. Lessons learned from successful Papanicolaou cytology cervical cancer prevention in the Socialist Republic of Vietnam. Diagn Cytopathol 2012;40(4):355–66.
27. Zhang S, Xu H, Zhang L, Qiao Y. Cervical cancer: epidemiology, risk factors and screening. Chin J Cancer Res 2020;32(6): 720–8.
28. Brianti P, De Flammineis E, Mercuri SR. Review of HPV-related diseases and cancers. New Microbiol 2017;40(2):80–5.
29. McIntyre-Seltman K, Castle PE, Guido R, Schiffman M, Wheeler CM. Smoking is a risk factor for cervical intraepithelial neoplasia grade 3 among oncogenic human papillomavirus DNA-positive women with equivocal or mildly abnormal cytology. Cancer Epidemiol Biomarkers Prev 2005;14(5):1165–70.
30. Alam S, Conway MJ, Chen HS, Meyers C. The cigarette smoke carcinogen benzo[a]pyrene enhances human papillomavirus synthesis. J Virol 2008;82(2):1053–8.
31. Duc TQ, Anh LTK, Chi VTQ, Huong NTT, Quang PN. Second-hand smoking prevalence in vietnamese population aged 15 and older: a systematic review and meta-analysis. Subst Abuse 2022;16:11782218221086653.
32. Huong LTT, Tuyet Hanh TT, Hoang LT, et al. Secondhand smoke and socio-demographic associated factors: results from the Vietnam Population-Based Provincial Global Adult Tobacco Survey in 2022, 17. Tobacco Use Insights; 2024.
