# Supplementary material for: The rising burden of female cancer in Ethiopia (2000–2021) and projections to 2040: Insights from the global burden of disease study
Source: PLoS One. 2025 Oct 7;20(10):e0333787. doi: 10.1371/journal.pone.0333787 (PMC12503343; doi:10.1371/journal.pone.0333787)
Supplement: Attachment — Submitted filename: Reviwer comments 9.8.2025.docx [file pone.0333787.s004.docx]

The manuscript titled **“The Rising Burden of Female Cancers in Ethiopia: A 21-years National and Sub national Analysis of Breast, Cervical, Ovarian and Uterine Cancers (2000–2021) with Projections to 2040 – Findings from the Global Burden of Disease Study”** addresses a major public health issue in Ethiopia using nationally representative data from the Global Burden of Disease (GBD) 2021, which aligns with PLOS ONE’s focus on broad, impactful research. However, the manuscript **requires major revisions** before it can be considered for publication in peer-reviewed journal.

1- **Title & Abstract**

**Strengths**

- Descriptive and specifies scope (national & sub-national, 2000–2021, projections to 2040).
- Keywords match MeSH/public health terms.

**Issues & Recommendations**

- **Title length**: PLOS ONE prefers concise titles without unnecessary punctuation or overly long clauses.
  **Suggestion:** “Rising Burden of Female Cancers in Ethiopia (2000–2021) and Projections to 2040: Insights from the Global Burden of Disease Study”
- **Abstract clarity**:
  - Sentences are long; condense for easier reading.
  - Avoid subjective terms like “spectacular rise”; use neutral academic language.
  - Add a **brief limitations statement** in the abstract (PLOS encourages transparency).
- **Keywords**: Use terms consistent with PubMed indexing (e.g., “epidemiology” instead of “trend”).

2- **Introduction**

**Strengths**

- Provides context on global cancer burden and Ethiopian health transition.
- Identifies data gaps and justifies use of GBD data.

**Issues & Recommendations**

- **Redundancy**: Some sentences repeat ideas about the double burden of disease.
- **Flow**: The shift from global to Ethiopia-specific context could be more seamless.
- **Novelty**: Explicitly state what is **new** about this study compared to prior Ethiopian cancer burden research — e.g., “first sub-national projections to 2040” or “first GBD-based multi-cancer age-stratified burden analysis.”

**3. Methods**

**Strengths**

- Clearly states GBD data source, metrics, ARIMA model use, and sub-national breakdown.
- Identifies statistical software.

**Issues & Recommendations**

- **PLOS ONE requires reproducibility**: Include *exact search terms*, date of data download, and any specific GBD variables used.
- **Ethics**: Although GBD data are publicly available, explicitly state that *no human subjects were directly involved and IRB approval was not required*.

**4. Results**

**Strengths**

- Comprehensive coverage (national, sub-national, age-specific, risk factors, projections).

**Issues & Recommendations**

- **Too descriptive**: Several sections restate numeric values already in tables/figures — condense narrative and highlight *patterns* rather than every figure.
- **Typos**: “beast cancer” in figure captions — must be corrected.
- **Statistical interpretation**: Include p-values or confidence intervals where relevant to indicate statistical significance (even for descriptive trends).

**5- Discussion**

**Strengths**

- Interprets trends in context of global literature.
- Identifies policy implications for Ethiopia.

**Issues & Recommendations**

- **Overlapping with Results**: Some paragraphs repeat numerical findings — focus more on *interpretation*, *comparisons*, and *implications*.
- **Policy recommendations**: Expand on actionable steps, e.g., “Introduce HPV self-sampling,” “Implement national ovarian cancer screening pilots.”
- **Limitations**: Strengthen by discussing:
  - GBD model assumptions.
  - Underreporting from cancer registries.
  - Possible changes in diagnostic capacity affecting trends.
- **Novelty & contribution**: Explicitly state what this study adds to Ethiopian and African cancer literature.

**6. Conclusion**

**Strengths**

- Summarizes key findings and implications.

**Issues & Recommendations**

- Avoid restating all results — focus on **main take-home message** and **priority actions**.
- Add a sentence linking findings to Sustainable Development Goals (SDG 3.4 on NCD mortality reduction), which PLOS often highlights in global health work.

**7. References**

**Issues & Recommendations**

- **Formatting**: PLOS ONE uses *numbered Vancouver style* — ensure uniformity.
- **Completeness**: Several references lack volume/issue/page or DOI.
- **Recency**: Update with the latest 2024–2025 publications on African cancer epidemiology to strengthen the literature base.

**8. Figures & Tables**

**Issues & Recommendations**

- Correct all typos in captions.
- Include legends that explain acronyms (DALYs, YLDs, YLLs).
- Consider summarizing large numeric tables into **supplementary material** to streamline the main text.
