# Supplementary material for: The rising burden of female cancer in Ethiopia (2000–2021) and projections to 2040: Insights from the global burden of disease study
Source: PLoS One. 2025 Oct 7;20(10):e0333787. doi: 10.1371/journal.pone.0333787 (PMC12503343; doi:10.1371/journal.pone.0333787)
Supplement: Attachment — Submitted filename: Response to reviewer.docx [file pone.0333787.s006.docx]

**Point-by-point response**

**Subject: submission of revised manuscript**

**Manuscript ID:** PONE-D-25-35361

**Title:** The Rising Burden of Female Cancer in Ethiopia **(**2000–2021**)** and Projections to 2040: Insights from the Global Burden of Disease Study

**To:** **PLOS ONE**

**Dear editor/reviewers.**

Dear, all

We sincerely thank you for your detailed, insightful feedback and constructive comments on our manuscript. Your suggestions have been invaluable in enhancing the substance and content of our work.

We have carefully considered each comment and question rose by the each reviewer and editor has provided point-by-point responses, which are detailed on the following pages. Additionally, we have incorporated these changes into the revised manuscript. The manuscript has been reviewed by language professionals, and we have ensured compliance with the journal's guidelines.

**Reviewer #1**

The manuscript was presented and written in Standard English with no major errors which caused difficulties to understand, however I recognized some minor grammar mistakes that should be reviewed and corrected. All the grammar errors were marked and recommendations were noted in the reviewed version attached. Moreover, the manuscript mostly mentioned and discussed about screening along with early detection of the disorders and lacked discussion regarding primary prevention such as intervention to prevent risk factors of the diseases. Primary prevention should be mentioned more in the manuscript.

Please refer to this article for reference: <https://doi.org/10.1016/j.xagr.2025.100526>

**Response:**

We thank the reviewer **#1** for the careful reading and insightful feedback on our manuscript. We appreciate the positive note regarding the overall clarity and use of Standard English. We have addressed the minor grammar errors as marked in the reviewed version to ensure improved accuracy and readability throughout the manuscript.

Regarding the comment on primary prevention, we agree that emphasizing interventions targeting modifiable risk factors is essential. Accordingly, we have expanded the discussion section to incorporate more comprehensive coverage of primary prevention strategies, including public health interventions aimed at reducing exposure to key risk factors associated with female cancers in Ethiopia such as smoking cessation, reduction of high alcohol consumption, promotion of healthy diet and physical activity, and awareness campaigns addressing unsafe sexual practices linked to cervical cancer.

This addition complements our existing discussion on screening and early detection, offering a more holistic view of cancer control approaches necessary for effective reduction in female cancer burden in Ethiopia. We believe these revisions significantly strengthen the manuscript’s relevance and utility for policymakers and public health practitioners.

Thank you again for your valuable comments.

**Reviewer #2**

This study analyzed data from the Global Burden of Disease (GBD) 2021 to highlight the 21-year trends and future projections (up to 2040) of four primary female cancers—breast, cervical, ovarian, and uterine—in Ethiopia. It presents comprehensive insights into national and sub-national trends across incidence, prevalence, mortality, Years Lived with Disability (YLDs), Years of Life Lost (YLLs), and Disability-Adjusted Life Years (DALYs). Key findings indicate significant increases in breast and ovarian cancer burdens, while cervical cancer, despite reduced mortality, shows a rise in associated disability.

This study demonstrates notable strengths. The study provides a comprehensive national and sub-national analysis using large-scale GBD 2021 data, supported by long-term trends and ARIMA modeling for reliable projections. The consistent rise in all four cancer types indicates a likely increase in demand for diagnosis, treatment, and palliative care if current patterns continue. However, this study shares common GBD limitations, including heterogeneity data quality in low socioeconomic development regions and reliance on simulations, which may affect accuracy. Moreover, the analysis is based on population-level data, lacking individual-level information and case-type differentiation.

**Response to Reviewer**

Dear Reviewer **#**2, Thank you for your valuable feedback. We agree with your assessment regarding the use of population-level data. As you correctly noted, our analysis is based on GBD data, which, by its nature, is at the population level. This limitation, which we have now explicitly stated in our revised manuscript, means we were unable to analyze individual-level information and differentiate specific case types.

While this does constrain the scope of our analysis to broad trends rather than individual risk factors, the GBD methodology was the most appropriate approach for our study's objective. It allowed us to provide a standardized, comprehensive, and longitudinal overview of the rising burden of female cancers in Ethiopia, which is a crucial first step in a region with very limited cancer data. Your point has been well-taken, and we believe the clarification in the revised manuscript strengthens the transparency of our study's methodology and limitations.

**Comments**

1. Overall

1.1. The manuscript should be thoroughly proofread to ensure correct grammar, spelling, academic language, and adherence to formatting in accordance with the author's guidelines.
1.2. Abbreviations should be defined in full upon their first appearance in the manuscript and should not be redefined in subsequent sections.

**Response**

Dear Reviewer, Thank you for your valuable feedback. We have addressed both of your comments. First, we conduct a thorough proofreading of the manuscript to ensure correct grammar, spelling, academic language, and adherence to the specified author guidelines for formatting. Second, we will ensure that all abbreviations are defined in full upon their first appearance in the manuscript and are not redefined in subsequent sections. We appreciate you bringing these points to our attention, as they will significantly improve the quality and clarity of our manuscript.

**Comments:**
2. Abstract
2.1. The abstract should not exceed 300 words.
2.2. The Methods section of the abstract should be carefully reviewed to ensure clarity, semantic completeness, and proper capitalization of all proper nouns.

**Response:**

Dear Reviewer, Thank you for your feedback regarding the Methods section of our abstract. We have carefully reviewed and revised the section to address your comments.

**Comment**

2.3. The Results and Discussion sections should be reviewed and organized in a clear and consistent manner to enhance readability, with recommendations presented according to each disease group.

**Response**

Thank you again for your valuable feedback. The results and discussion sections have been reorganized and now present the findings and recommendations by each disease group to improve clarity and readability.

**Comments**

3. Introduction
   3.1. Typos should be carefully checked, and sentences should be written clearly, comprehensively, and coherently to enhance clarity and readability.
   3.2. The Introduction should provide a description of each sub-national region analyzed, along with a clear rationale for their selection—indicating whether they are representative of the broader region or the national population. If applicable, the characteristics and their influence on disease burden should be clearly outlined.

**Response:**

We appreciate the reviewer’s advice regarding language clarity and typos. We have carefully proofread the entire manuscript and revised sentences to ensure they are clear, comprehensive, and coherent, significantly improving readability throughout the text.

Regarding the Introduction, we have included all sub-national regions and city administrations of Ethiopia during the period from 2000 to 2021 in our analysis. We explicitly state that all these regions were included to provide a comprehensive national picture rather than a selected sample. Additionally, we briefly outline important regional characteristics (e.g., population density, urbanization, healthcare access) that may influence the cancer burden, providing clear rationale for their inclusion. This contextual information enhances understanding of regional disparities in female cancer burden within Ethiopia.

**Comment**

4. Methods
4.1. The Methods section should be supplemented with appropriate references and citations for the methodologies applied in the study.

**Response**

Thank you for this feedback. We have reviewed the Methods section and have now added appropriate references and citations for all methodologies used in the study. The revised manuscript reflects these changes and is ready for your review.

**Comment**

4.2. The final paragraph beginning with “This method aims to lay the foundation...” functions as a conclusion and should be moved to the Discussion section rather than remaining in the Methods.
**Response**

We have moved the paragraph beginning with "This method aims to lay the foundation..." from the Methods section to the Discussion section as suggested. The revised manuscript is ready for your review.

**Comment**

5. Results
5.1. This section should be revised using varied and clear descriptions to enhance readability and logical flow. Abbreviations previously defined may be used where appropriate. Furthermore, this part should focus solely on presenting the results without interpretation. The authors' comments or explanations at the end of each subsection should be moved to the Discussion section.

**Response**

Thanks for the feedback. We have revised the Results section to improve readability and logical flow using varied and clear descriptions. We have also moved all interpretations and explanations to the Discussion section, ensuring the Results section focuses solely on presenting the data. The revised manuscript is ready for your review.

**Comment**

5.2. In the subsection “The national prevalence, incidence and death Trends of Female breast, cervical, ovarian and uterine cancer in Ethiopia (2000-2021)”, the mortality is not addressed and should be included. While cervical cancer shows an overall increase in prevalence and incidence, one segment demonstrates a decline—this exception should be clearly identified and discussed.

**Response**

Thank you for your feedback. We have revised the subsection to include the mortality trends. We have also explicitly identified and discussed the specific segment of cervical cancer that shows a decline in prevalence and incidence, as you suggested. The updated manuscript is ready for your review.
5.3. In the subsection “The national prevalence, incidence and death Trends of Female breast, cervical, ovarian and uterine cancer in Ethiopia (2000-2021)”, the sentence “Lastly, uterine cancer incidence nearly double…” is unclear or inappropriate and should be reviewed and revised for accuracy and clarity.

**Response:**

Thank you for your feedback. We have reviewed the sentence.

5.4. The subsection “Sub-National prevalence, Incidence and mortality trends of beast, cervical, ovarian and uterine cancer in Ethiopia (2000-2021)” should place greater emphasis on describing mortality patterns, rather than focusing primarily on prevalence and incidence.

**Response:**

Thank you for the feedback. We have revised the subsection to include and focus on mortality patterns. The revised manuscript is ready for your review.

**Comment**

5.5. The subsection “Sub-National prevalence, Incidence and mortality trends of beast, cervical, ovarian and uterine cancer in Ethiopia (2000-2021)”, part “Cervical cancer”: Regional variations in trends should be further elaborated. Although cervical cancer incidence and prevalence increased and mortality declined overall, some regions deviated from this pattern. These discrepancies should be clearly described and analyzed.

**Response:**

Thank you for your feedback. We have revised the subsection to further elaborate on regional variations. As you suggested, we have clearly described the specific regions that deviated from the overall national trend of increasing incidence and prevalence and declining mortality in discussion part. The revised manuscript is ready for your review.

**Comments**

5.6. The subsection “Sub-National prevalence, Incidence and mortality trends of beast, cervical, ovarian and uterine cancer in Ethiopia (2000-2021)”, part “Ovarian cancer”: Even though incidence, prevalence, and mortality increased across regions, the extent of these increases varied notably. The results should emphasize these regional differences to better understand the disparities.

**Response:**

Thank you for your valuable feedback. We have revised the manuscript to incorporate a more detailed analysis of the sub-national trends for ovarian cancer, emphasizing the regional disparities as you suggested. We believe these revisions significantly strengthen the manuscript.

**Comment**
5.7. In the subsection “Deconstructing the Total Health Loss: Disability-Adjusted Life Years, Years Lived with Disability, and Years of Life Lost for Female Breast, Cervical, Ovarian, and Uterine Cancers in Ethiopia (2000–2021)”, the title “Cervical cancer: a complex picture of decreasing mortality but rising disability” is not yet supported by a clear and accurate description of the corresponding data trends. While the title suggests rising disability, the opening sentence of the paragraph appears inconsistent with the reported data. Specifically, from approximately 2000 to 2021, DALYs decreased sharply, YLDs increased slightly, and YLLs declined marginally.

**Response:**

Thank you for your valuable feedback. We have carefully reviewed your comment and revised the manuscript accordingly. The new title for the subsection is "Cervical cancer: decreasing mortality alongside modest rise in disability," which we believe more accurately reflects the data trends. The revised paragraph now provides a clear and consistent description of the data, highlighting the decrease in total DALYs and YLLs while acknowledging the modest rise in YLDs. We are confident that these changes address your concerns and provide a more precise representation of our findings.

**Comment**
5.8. The subsection titled “Age-standardized prevalence, incidence, and mortality rate of breast, cervical, uterine, and ovarian cancer in Ethiopia” should be placed before the subsection “Age-standardized burden of female breast, cervical, ovarian, and uterine cancers in Ethiopia: A Comparative Analysis, 2000 and 2021” to ensure a more logical and coherent flow of information.
**Response:**

Thank you for the feedback. We have reordered the manuscript sections as you suggested improving the logical flow.

**Comment**
6. Discussion

6.1. The Discussion section should be reviewed and rewritten in an academic style, ensuring that interpretive or clarifying comments—currently placed in the Results section—are appropriately relocated. Revisions should also reflect any updates to the Results section, with careful attention to spelling and grammar accuracy. Furthermore, the Discussion section should include more in-depth explanations and supporting arguments to better interpret and contextualize the results.

6.2 The third paragraph beginning with “The trend of cervical cancer is slightly more complex…” the sentences: “This result was consistent with the global studies specifically in breast cancer (14, 15). This is primarily because YLLs have decreased, indicating that those women who were dying from the disease are not losing lives to it because screening and treatment options are improving.” This statement is confusing and should be carefully reviewed and revised for clarity, accuracy, and logical consistency.

**Response:**

Thank you for your comment. We have revised the paragraph to clarify that the decline in DALYs is due to reduced YLLs from better screening and treatment, while the increase in YLDs reflects ongoing disability, highlighting the need for supportive care.

**Comment:**
6.3. The subsection titled “Sub-National Dynamics: Understanding Local Realities” should emphasize the key disease patterns in each region, providing detailed explanations for these trends. It should also discuss the underlying regional characteristics and how they reflect broader national challenges and health system issues.

**Response:**

Thank you for your comment. We have enhanced the “Sub-National Dynamics” section to clearly highlight key disease patterns and provide explanations based on regional population density, healthcare access, and risk factors. We also discuss how these regional differences reflect broader national health system challenges and emphasize the need for tailored, region-specific cancer control strategies.

**Comment**
6.4. The final paragraph beginning with “According to the risk factor analysis result…” in the subsection “Age-Standardized Burden: Who is Most Affected?” could be improved by briefly outlining the underlying mechanisms through which the identified risk factors influence disease burden. Additionally, it should clarify how these factors may be modified through targeted interventions.

**Response:**

Thank you for the helpful suggestion. We have revised the risk factors section to briefly explain how key factors—such as diet, smoking, obesity, and unsafe sex—contribute biologically to female cancers in Ethiopia. We also highlighted modifiable lifestyle factors and interventions like HPV vaccination and screening that can reduce cancer burden. These changes improve clarity and link epidemiological data to actionable prevention strategies.

**Reviewer #3**

The manuscript explores increasing burden of female cancers in Ethiopia. The manuscript is technically sound and well written. In My opinion authors should make following amendment in their manuscript. Authors should make a comparative study about increasing burden of female cancers around the Globe initially, in the introduction section and then proceed towards Ethiopia.

**Response**

We sincerely thank the reviewer **#3** for the constructive suggestion to include a comparative overview of the increasing burden of female cancers globally in the Introduction section before focusing on Ethiopia.

In response, we have revised the Introduction to begin with a brief summary of global trends in female cancers, highlighting incidence and mortality patterns worldwide and in different regions. This comparative context sets the stage for a focused discussion on the specific situation in Ethiopia, emphasizing how the country fits within these broader global trends. We believe this addition strengthens the manuscript by providing readers with a clearer understanding of the issue’s global relevance and Ethiopia’s position in the worldwide cancer landscape.

Thank you for your valuable input, which has enhanced the clarity and impact of our work.

**Reviewer #4**

The manuscript titled **“The Rising Burden of Female Cancers in Ethiopia: A 21-years National and Sub national Analysis of Breast, Cervical, Ovarian and Uterine Cancers (2000–2021) with Projections to 2040 – Findings from the Global Burden of Disease Study”** addresses a major public health issue in Ethiopia using nationally representative data from the Global Burden of Disease (GBD) 2021, which aligns with PLOS ONE’s focus on broad, impactful research. However, the manuscript **requires major revisions** before it can be considered for publication in peer-reviewed journal.

**Comments**

1- **Title & Abstract**

**Strengths**

- Descriptive and specifies scope (national & sub-national, 2000–2021, projections to 2040).
- Keywords match MeSH/public health terms.

**Issues & Recommendations**

- **Title length**: PLOS ONE prefers concise titles without unnecessary punctuation or overly long clauses.
  **Suggestion:** “Rising Burden of Female Cancers in Ethiopia (2000–2021) and Projections to 2040: Insights from the Global Burden of Disease Study”
- **Abstract clarity**:
  - Sentences are long; condense for easier reading.
  - Avoid subjective terms like “spectacular rise”; use neutral academic language.
  - Add a **brief limitations statement** in the abstract (PLOS encourages transparency).
- **Keywords**: Use terms consistent with PubMed indexing (e.g., “epidemiology” instead of “trend”).

**Response**: We thank the reviewer 4 for these valuable suggestions. We have revised the manuscript to address each point:

- **Title:** The title has been shortened to "Rising Burden of Female Cancers in Ethiopia (2000–2021) and Projections to 2040: Insights from the Global Burden of Disease Study."
- **Abstract:** The abstract has been rewritten for conciseness, removing subjective terms and using neutral language. We have also added a brief statement on the study's limitations.
- **Keywords:** The keywords have been reviewed and updated to be consistent with common indexing terms.

**Comments**

2- **Introduction**

**Strengths**

- Provides context on global cancer burden and Ethiopian health transition.
- Identifies data gaps and justifies use of GBD data.

**Issues & Recommendations**

- **Redundancy**: Some sentences repeat ideas about the double burden of disease.
- **Flow**: The shift from global to Ethiopia-specific context could be more seamless.
- **Novelty**: Explicitly state what is **new** about this study compared to prior Ethiopian cancer burden research — e.g., “first sub-national projections to 2040” or “first GBD-based multi-cancer age-stratified burden analysis.”

**Response**:

We appreciate the reviewer's detailed feedback. We have revised the manuscript to address these points. Specifically, we have removed redundant sentences to improve conciseness and have refined the narrative to ensure a smoother transition from the global context to the specific situation in Ethiopia. Furthermore, we have explicitly stated our study's unique contributions in the introduction, clarifying its novelty as the first GBD-based analysis to provide sub-national projections to 2040 for female cancers in the country.

**Comments**

**3. Methods**

**Strengths**

- Clearly states GBD data source, metrics, ARIMA model use, and sub-national breakdown.
- Identifies statistical software.

**Issues & Recommendations**

- **PLOS ONE requires reproducibility**: Include *exact search terms*, date of data download, and any specific GBD variables used.
- **Ethics**: Although GBD data are publicly available, explicitly state that *no human subjects were directly involved and IRB approval was not required*.

**Response**:

We thank the reviewer for their valuable feedback. We have revised the manuscript to address both points. In the methods section, we have added a statement ensuring full reproducibility by including the specific search parameters, data variables, and the date of data retrieval from the Global Burden of Disease (GBD) database. Furthermore, we have added a sentence clarifying that because the study used publicly available data and did not involve human subjects, institutional review board (IRB) approval was not required.

**Comments**

**4. Results**

**Strengths**

- Comprehensive coverage (national, sub-national, age-specific, risk factors, projections).

**Issues & Recommendations**

- **Too descriptive**: Several sections restate numeric values already in tables/figures — condense narrative and highlight *patterns* rather than every figure.
- **Typos**: “beast cancer” in figure captions — must be corrected.
- **Statistical interpretation**: Include p-values or confidence intervals where relevant to indicate statistical significance (even for descriptive trends).

**Response**:

We appreciate the reviewer's detailed feedback. We have revised the manuscript to address these points. Specifically, we have condensed some narrative to avoid repeating numerical values already presented in the figures and tables. We have also carefully proofread and corrected all typographical errors. Furthermore, we have ensured that the 95% uncertainty intervals (UIs) are consistently included to provide a clear indication of statistical interpretation.

**Comments**

**5- Discussion**

**Strengths**

- Interprets trends in context of global literature.
- Identifies policy implications for Ethiopia.

**Issues & Recommendations**

- **Overlapping with Results**: Some paragraphs repeat numerical findings — focus more on *interpretation*, *comparisons*, and *implications*.
- **Policy recommendations**: Expand on actionable steps, e.g., “Introduce HPV self-sampling,” “Implement national ovarian cancer screening pilots.”
- **Limitations**: Strengthen by discussing:
  - GBD model assumptions.
  - Underreporting from cancer registries.
  - Possible changes in diagnostic capacity affecting trends.
- **Novelty & contribution**: Explicitly state what this study adds to Ethiopian and African cancer literature.

**Response**:

We thank the reviewer for their valuable feedback. We have revised the manuscript to address all of the points raised, which we believe have significantly strengthened the paper. We have condensed the narrative to avoid repeating numerical findings and now focus on interpretation, comparisons, and implications. We have also expanded our policy recommendations to include more specific and actionable steps. The limitations section has been strengthened by discussing GBD model assumptions, underreporting, and changes in diagnostic capacity. Finally, we have explicitly stated the study's unique contribution to the Ethiopian and African cancer literature in the introduction, clarifying its novelty as the first GBD-based analysis to provide sub-national projections to 2040 for female cancers in the country.

**Comments**

**6. Conclusion**

**Strengths**

- Summarizes key findings and implications.

**Issues & Recommendations**

- Avoid restating all results — focus on **main take-home message** and **priority actions**.
- Add a sentence linking findings to Sustainable Development Goals (SDG 3.4 on NCD mortality reduction), which PLOS often highlights in global health work.

**Response:**

We appreciate the reviewer's comprehensive and constructive feedback. We have revised the manuscript to address all of the points raised, which we believe have significantly strengthened the paper. We have rewritten the conclusion to be more concise, focusing on the main take-home messages and priority actions. Furthermore, we have added a sentence linking our findings to Sustainable Development Goal (SDG) 3.4, highlighting the relevance of our work in reducing premature mortality from non-communicable diseases. We are confident that these revisions have improved the overall quality of the manuscript and thank the reviewer for their valuable guidance.

**Comments**

**7. References**

**Issues & Recommendations**

- **Formatting**: PLOS ONE uses *numbered Vancouver style* — ensure uniformity.
- **Completeness**: Several references lack volume/issue/page or DOI.
- **Recency**: Update with the latest 2024–2025 publications on African cancer epidemiology to strengthen the literature base.

**Response**:

Thank you for your valuable feedback. We have addressed all of your points regarding the reference list. Additionally, we have updated all references to include complete information such as volume, issue, page numbers, and DOIs. We have also incorporated recent publications to strengthen the literature base and provide the most up-to-date context for our findings.

**Comments**

**8. Figures & Tables**

**Issues & Recommendations**

- Correct all typos in captions.
- Include legends that explain acronyms (DALYs, YLDs, and YLLs).
- Consider summarizing large numeric tables into **supplementary material** to streamline the main text.

**Response**:

Thank you for your valuable feedback. We have addressed all of your points regarding the manuscript's figures and tables. We have carefully corrected all typos in the captions, including the specific error you identified. We have also added clear legends to all figures to define acronyms such as DALYs, YLDs, and YLLs

**We sincerely thank our reviewers and editors for their constructive feedback and careful consideration, which have greatly enhanced the quality and clarity of our manuscript.**
