# Supplementary material for: The rising burden of female cancer in Ethiopia (2000–2021) and projections to 2040: Insights from the global burden of disease study
Source: PLoS One. 2025 Oct 7;20(10):e0333787. doi: 10.1371/journal.pone.0333787 (PMC12503343; doi:10.1371/journal.pone.0333787)
Supplement: Attachment — Submitted filename: Point by point response R2.docx [file pone.0333787.s007.docx]

**Point by point response**

**Manuscript ID**: PONE-D-25-35361R1

**Title:** The Rising Burden of Female Cancer in Ethiopia (2000–2021) and Projections to 2040: Insights from the Global Burden of Disease Study

Dear Dr. Thien Tan Tri Tai Truyen,

Thank you for the opportunity to revise our manuscript. We are grateful to you and the reviewers for the constructive feedback. We have carefully addressed all the comments raised during the review process. We have also included a marked-up copy of the manuscript showing the changes.

Please find our point-by-point response to the reviewer comments below.

**Reviewer #1, Reviewer #3, and Academic Editor Comments**

**Comment**: The manuscript has been reviewed and found to have adequately addressed all comments. It is now acceptable for publication with a minor revision.

**Response**: We sincerely thank the reviewers and the editor for their positive feedback. We are pleased that the previous revisions have successfully addressed their concerns and that the manuscript is now in a state of readiness for publication, pending one final change.

**Reviewer #2 Comments**

**Comment**: The abstract still exceeds the 300-word limit and must be shortened to comply with *PLOS ONE's* guidelines.

**Response**: We appreciate the reviewer for highlighting this critical point. We apologize for the oversight in our previous revision. We have now meticulously revised the abstract to ensure it **adheres to the 300-word limit** as required by the journal's guidelines. The revised abstract concisely presents the study's key findings while maintaining all essential information.

We believe these revisions have significantly improved the manuscript and that it now fully meets the publication criteria of *PLOS ONE*. We look forward to your positive response.

Sincerely,

Molalign Aligaz Adisu and Co-authors
